# Aza Analogs of the TRPML1 Inhibitor Estradiol Methyl Ether (EDME)

**DOI:** 10.3390/molecules28217428

**Published:** 2023-11-04

**Authors:** Philipp Rühl, Franz Bracher

**Affiliations:** Department of Pharmacy, Center for Drug Research, Ludwig-Maximilians University, 80539 Munich, Germany; philipp.ruehl@cup.lmu.de

**Keywords:** cation channels, TRPML1, bioisostere, steroids, estrogens, ring cleavage, protective groups, pyridines, pyrimidines, cyclization

## Abstract

Estradiol methyl ether (**EDME**) has recently been described by us as a very potent and subtype-specific inhibitor of the lysosomal cation channel TRPML1. Following the principle of bioisosteres, we worked out efficient synthetic approaches to ring-A aza-analogs of EDME, namely a methoxypyridine and a methoxypyrimidine analog. Both target compounds were obtained in good overall yields in six and eight steps starting from 19-nortestosterone via the oxidative cleavage of ring A followed over several intermediates and with the use of well-selected protective groups by re-cyclization to provide the desired hetero-analogs. The methoxypyridine analog largely retained its TRPML1-inhibitory activity, whereas the methoxypyrimidine analog significantly lost activity.

## 1. Introduction

TRPML1 is one of three members (TRPML1-3) of the TRPML cation channels group, a subfamily within the transient receptor potential (TRP) superfamily. As a non-selective lysosomal channel permeable to Ca^2+^, Na^+^, Fe^2+^, Zn^2+^ and other cations, it plays an important role in multiple physiological processes but also in several human diseases. A mutation with loss of function of TRPML1 causes Mucolipidosis Type IV, a neurodegenerative lysosomal storage disorder [1]. Furthermore, TRPML1 has gained interest as it is associated to be involved in various processes in different cancers, e.g., melanoma [2] and non-small lung cancer [3], and its influence on cardiovascular [4] and neurodegenerative diseases has been discussed [5].

Therefore, obtaining access to inhibitors and activators for this target as pharmacological tools or even as possible future therapeutic options is of great interest. 

Phosphatidylinositol 3,5-bisphosphate (PI(3,5)P2), a major constituent of the lysosomal membrane, has been described as an endogenous activator of all TRPML channels, while phosphatidylinositol 4-5-bisphosphate (PI(4,5)P2), which is mainly found in the plasma membrane, has been identified as endogenous inhibitor of TRPML1 and TRPML3 [6]. Due to their structural characteristics (polarity), these two molecules are not suitable as pharmacological tools as they cannot permeate cell membranes.

As a consequence, several low-molecular activators and inhibitors of TRPML1 with suitable pharmacokinetic properties have been developed in recent years. While MK6-83 [7]*,* SF-51 and ML-SA1 [8] are examples of unselective TRPML activators, the only known selective activator of TRPML1 is ML1-SA1 [9]. Despite the evident need for potent TRPML1 inhibitors, the number of these is still limited.

**Figure 1:**
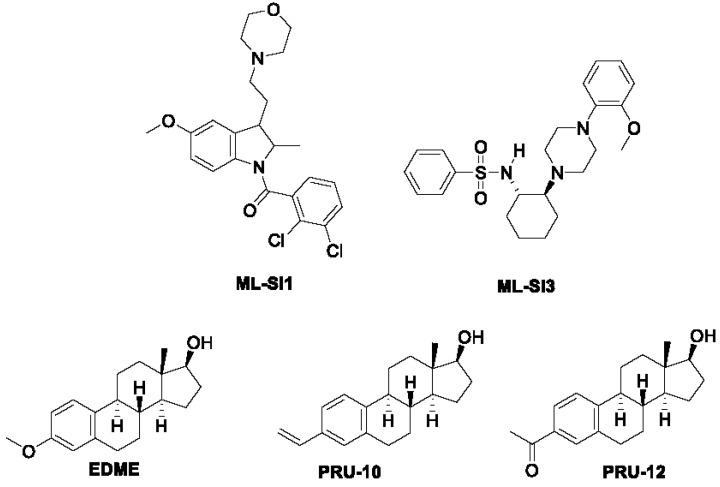
Structures of published TRPML inhibitors.

While the indoline derivative **ML-SI1** and the 1,2-diaminocyclohexane derivative **ML-SI3** are known examples in the literature for isoform-unselective inhibitors [10,11,12], we described the steroidal compound estradiol methyl ether (**EDME**) as the first highly potent (IC_50_ 0.6 μM) and subtype-selective TRPML1 inhibitor in our previous work (Figure 1) [13]. This inhibitor was identified by random screening of 2,430 compounds on hTRPML1ΔNC-YFP, a plasma membrane variant of wild-type TRPML1. Subsequently, we tested other pharmacologically relevant steroidal compounds and found that natural and synthetic steroids lacking aromaticity in ring A (the typical structure motif of estrogens) are virtually inactive (cholesterol, phytosterols, glucocorticoids, mineralocorticoids, antiestrogens, antiandrogens, 5α-reductase inhibitors). In the class of estrogens, the native hormone 17β-estradiol showed significantly reduced inhibition (IC_50_ 5.3 μM) and only mestranol, a congener of EDME bearing an additional ethinyl group at C-17, showed considerable activity. Inversion of the configuration of the 17α-hydroxy group eliminated inhibitory activity in all cases. Finally, we synthesized ten modified versions of EDME, most of which have a replacement of the methoxy group at C-3 in common with a lipophilic residue. Out of these, the 3-vinylestrane **PRU-10** (IC_50_ 0.41 μM) and the 3-acetyl derivative **PRU-12** (IC_50_ 0.28 μM) (Figure 1) showed stronger TRPML1 inhibition, an improved selectivity profile compared to **EDME**, and reduced estrogenic activity.

Based on this preliminary evidence on structure–activity relationships, we focussed on additional modifications of ring A of **EDME**. Due to our own positive experience with the synthesis of aza analogs of steroidal lead structures for improving or modulating biological activities [14,15], we decided to investigate a pyridine-type 4-aza analog (**1**) and a pyrimidine-type 2,4-diaza analog (**2**) of **EDME**.

Following the well-established principle of “bioisosteres” [16], single functional groups in a bioactive molecule can be replaced by other, more or less similar groups in order to extend or improve potency, enhance selectivity, alter the physicochemical properties or metabolism, or improve pharmacokinetics or toxicity. The bioisosteric replacement of phenyl rings can be performed in a classical manner with the introduction of neutral aromatic rings (thiophene, furan) or azaarenes (pyridine, pyrimidine, pyrazine) [17], further “nonclassical” biosisosteres (acetylene, bridged aliphatic ring systems) have been developed [18]. The azaarene bioisosteres have gained significant interest since they can introduce basic properties as well as H-bond acceptor and/or H-bond donor properties and thus improve (or reduce) the interaction with the target protein.

## 2. Results and Discussion

### 2.1. Chemistry

As we intended to synthesize both target compounds in an enantiomerically pure form, we selected a “chiral pool” approach [19] for our syntheses. Estradiol or **EDME** were not suitable starting materials for this approach due to the lack of feasible synthetic methods for the conversion of phenols/phenol ethers into pyridines and pyrimidines. The same holds for non-estrogenic sterols bearing a methyl group (C-19) at C-10 since this residue would prevent aromatization of ring A. For our purposes, 19-nortestosterone (nandrolone; **3**) was identified as the best precursor for a couple of reasons: this (commercially available and affordable) homochiral compound already has the required configurations at the stereocenters in rings C and D, its ring A is a cyclohexenone that can be cleaved by oxidation, and, last but not least, there is no methyl group at C-10. The published oxidative degradation of 19-nortestosterone (**3**) under the cleavage of the C-4,C-5 bond and decarboxylation yields a ketocarboxylic acid of type **A** [20]. The formal integration of ammonia and oxidative aromatization should provide a ring A pyridone, which was then to be *O*-methylated to provide the desired 4-aza analog **1** of **EDME**. 

**Figure 2:**
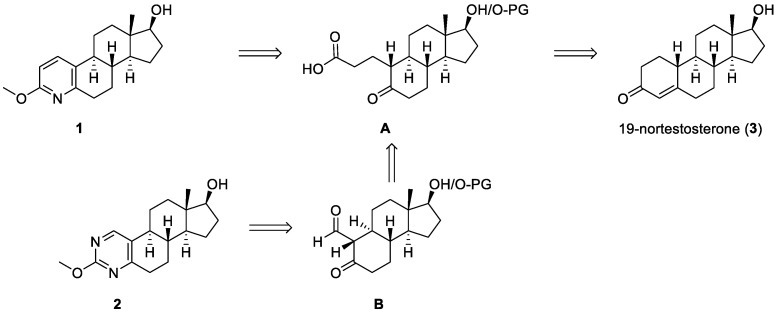
Retrosynthesis of the target compounds **1** and **2** (PG—protective group).

Oxidative degradation of the propionate side chain in **A** [20] would provide a ketoaldehyde of type **B**, which, upon treatment with *O*-methylisourea, should provide the methoxypyrimidine target compound **2**. In both series, temporary protection of the 17-OH group had to be considered (Figure 2).

#### 2.1.1. 4-Aza Analog of EDME

**Scheme 1:**
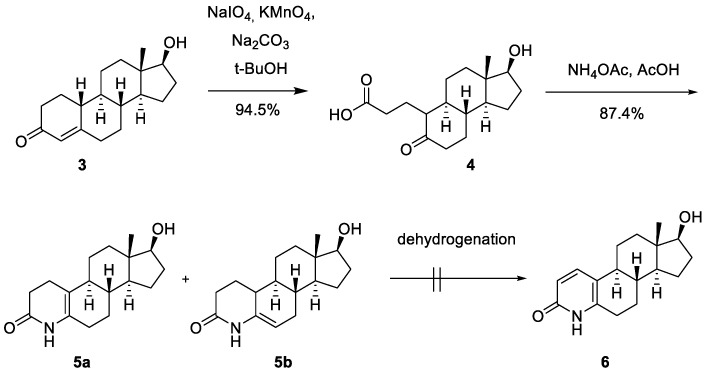
First attempt for the synthesis of the 4-aza analog **1** of EDME.

Our chiral pool approach started with the oxidative cleavage of ring A of 19-nortestosterone (**3**) to provide ketocarboxylic acid **4**. While Holt et al. [20] described an ozonolysis protocol with a yield of 50%, we obtained **4** in a yield of 94.5% by using NaIO_4_/KMnO_4_ as the oxidant, a method established for a related degradation of a 19-methyl steroid in the course of the synthesis of the drug finasteride [21]. Subsequent treatment with ammonium acetate in acetic acid under reflux [22] resulted in ring closure to two poorly separable unsaturated lactams, **5a** with a Δ5,10- and **5b** with a Δ5,6 double bond (yield: 87.4%, ratio **5a**:**5b**: 15:85). Unfortunately, we could not find a suitable oxidant for direct dehydrogenation of these lactams to the ring A pyridone **6** (Scheme 1). Most likely, the unprotected 17-OH group interfered with the examined oxidants (DDQ, iodine-based reagents, and others). As a consequence, we examined protective groups for 17-OH. 

Our first attempts to utilize MOM protection of the starting material 19-nortestosterone (**3**) failed early due to problems with introducing this protective group. The following experiments using TBDMS protection gave promising results in the early steps (for details, see Supporting Information) but failed due to the instability of the TBDMS ether as soon as experiments were performed under acidic conditions (no details shown).

Finally, we turned to benzyl protection of 17-OH. Surprisingly, standard protocols for the protection of this secondary alcohol under alkaline (NaH/benzyl halides) or acidic conditions [23] (benzyl trichloroacetimidate/TFA) failed to provide acceptable yields. However, Dudley’s protocol [24] utilizing 2-benzyloxy-1-methylpyridinium triflate/MgO gave the desired benzyl ether **7** in 82.1% yield. Following the general strategy described above, oxidative ring A degradation with NaIO_4_/KMnO_4_ yielded ketocarboxylic acid **8** in a 99.2% yield. Subsequent treatment with ammonium acetate in acetic acid under reflux resulted in ring closure to two (still poorly separable) unsaturated lactams, **9a** with a Δ5,10- and **9b** with a Δ5,6 double bond (73.4% yield of the mixture, ratio **9a**:**9b**: 15:85).

With this mixture of isomers in hand, we again investigated numerous reagents used in previous publications to dehydrogenate dihydropyridines. These included treatment with MnO_2_ [25] (result: no conversion), Pb(OAc)_4_ [26] (result: decomposition), air oxidation [27] (result: no conversion), and treatment with KMnO_4_ (result: decomposition).

As all of these experiments failed, we attempted formal dehydrogenation via halogenation at the methylene group next to the lactam carbonyl, followed by dehydrohalogenation utilizing published reagents from the pyridone and related fields (SO_2_Cl_2_ [28], iodotrimethylsilane [29], CuBr_2_ [30]). Neither of these reagents gave noteworthy amounts of the dehydrogenation product. Finally, treating **9a**/**9b** with a combination of reagents (CuBr_2_, LiBr, 1,3-dimethoxybenzene, trifluoromethanesulfonic acid in acetonitrile) that was originally used for a cyclohexenone-to-phenol conversion in 19-norandrost-4-en-3-ones [31] gave the desired pyridone **10** in 33.8% yield. This product was accompanied by small amounts (7.8%) of the 17-*O*-deprotected pyridone **6** (see Scheme 1).

**Scheme 2:**
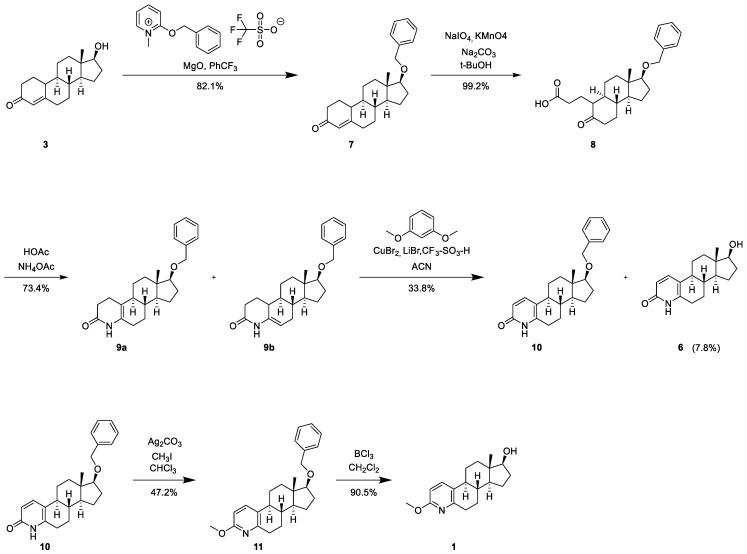
Successful approach to the 4-aza analog **1** utilizing benzyl protection at 17-OH.

*O*-Methylation of **10** to provide the methoxypyridine derivative **11** was achieved in 47.2% yield using iodomethane/Ag_2_CO_3_ [32]. As the final step, the benzyl protective group at 17-OH had to be removed without affecting the methoxypyridine unit. This step turned out to be more difficult than expected. Under standard *O*-debenzylation conditions (hydrogenolysis under Pd catalysis), no conversion was achieved; an alternative Pd-catalyzed method using Et_3_SiH as the reductant [33] failed as well. A published method for the selective cleavage of benzyl ethers utilizing CrCl_2_/LiI [34] surprisingly led to the cleavage of the methyl ether at the pyridine ring and left the benzyl ether untouched. Pyridone **10** (the precursor of **11**) was obtained in a 90% yield. Finally, the desired *O*-debenzylation was achieved by means of BCl_3_ [35]. The carbinol **1** was obtained in a 90.5% yield, and the methoxy group at C-3 was not affected (Scheme 2).

#### 2.1.2. 2,4-Diaza (pyrimidine) Analog of **EDME**

As mentioned above (Figure 2), the methoxypyrimidine motif of the target compound **2** should be built up by cyclocondensation of a ketoaldehyde of type **B** with *O*-methylisourea. This approach has, in principle, been published before in a French patent claimed by Roussel Uclaf in 1967 [36]; however, this route started with a fully synthetic precursor [37] of undefined stereochemistry (most likely racemic), and neither full experimental details nor acceptable spectroscopic data on the characterization of intermediates and the final product were presented.

**Scheme 3:**
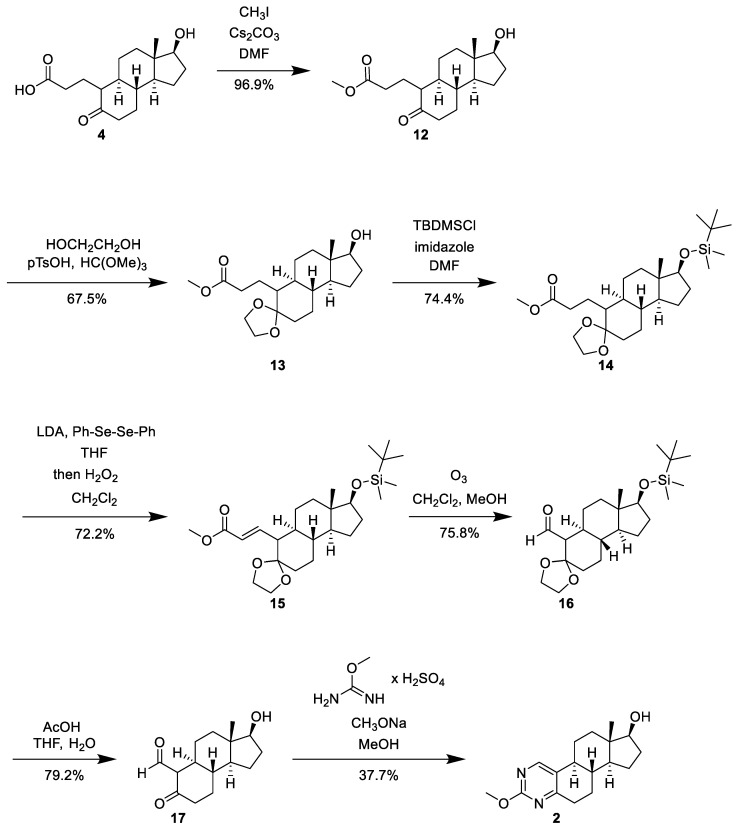
Synthesis of the 2,4-diaza analog **2** of **EDME**.

Our chiral pool approach started once again with 19-nortestosterone (**3**). For this new purpose, the ketocarboxylic acid **4** obtained by oxidative cleavage of ring A (Scheme 1) needed to be degraded further in order to convert the propionate side-chain into a formyl group (see Figure 2) following, in general, a poorly detailed protocol published by Holt et al. [20]. For this purpose, ketocarboxylic acid **4** was first converted into its methyl ester **12** via a higher-yielding protocol utilizing iodomethane/Cs_2_CO_3_ (96.9% yield), followed by conversion of the keto group into the dioxolane **13** (67.5% yield). Next, and distinct from the Holt protocol, the 17-OH group was protected by conversion into the TBDS ether **14** (74.4% yield), in order to circumvent interference of the acidic 17-OH group with the strong base LDA required for the following step. Then, the methyl propionate side chain was converted into the α,β-unsaturated ester **15** in 72.2% yield by a selenation-selenoxide elimination protocol including treatment with LDA/diphenyldiselenide and oxidation with H_2_O_2_ [38], followed by spontaneous elimination. Two-carbon degradation was then performed by ozonolysis followed by work-up with dimethyl sulfide to provide the aldehyde **16** in 75.8% yield. The treatment of **16** with acetic acid in THF-water resulted in simultaneous deprotection of the dioxolane and the TBDS ether to provide the ketoaldehyde **17** in 79.2% yield. Finally, treatment with *O*-methylisourea gave the target methoxypyrimidine **2** in 37.7% yield (Scheme 3).

### 2.2. Biological Testing

The two target compounds, pyridine analog **1** and pyrimidine analog **2**, as well as the inadvertently obtained pyridone analog **6** were submitted to our previously described [13] test for inhibition of TRPML1 on hTRPML1ΔNC-YFP, a plasma membrane variant of wild-type TRPML1 by means of a fluorimetric Ca^2+^ influx assay. The results are shown in Table 1.

Compared to EDME, the 4-aza analog **1** showed slightly reduced TRPML1-inhibitory activity (factor <2 less potent), the 2,4-diaza analog **2**; however, it is only a very weak inhibitor, and the pyridone analog 6 is virtually inactive.

## 3. Materials and Methods

### 3.1. Chemistry

All NMR spectra (^1^H, ^13^C, DEPT, H-H-COSY, HSQC, HMBC) were recorded at 23 °C on an Avance III 400 MHz Bruker BioSpin or Avance III 500 MHz Bruker BioSpin instrument (Bruker, Billerica, MA, USA) unless otherwise specified. Chemical shifts *δ* are stated in parts per million (ppm) and are calibrated using residual protic solvents as an internal reference for proton (CD_2_Cl_2_: *δ* = 5.32 ppm, MeOD *δ* = 3.31 ppm, DMSO: *δ* = 2.50 ppm) and for carbon the central carbon resonance of the solvent (CD_2_Cl_2_: *δ* = 53.84 ppm, MeOD *δ* = 49.00 ppm, DMSO: *δ* = 39.52 ppm). Multiplicity is defined as s—singlet, d—doublet, t—triplet, q—quartet, and m—multiplet. NMR spectra were analyzed with the NMR software MestReNova, version 12.0.1-20560 (Mestrelab Research S.L., Santiago de Compostela, Spain). Numbering of the carbon atoms in seco-steroids: For the sake of comparability, we kept using the numbering the single carbon atoms had in the intact steroids, since, in the following, the seco-steroidal intermediates were cyclized to the azasteroids later. High-resolution mass spectra were performed by the LMU Mass Spectrometry Service applying a Thermo Finnigan MAT 95 (Thermo Fisher Scientific, Waltham, MA, USA) or Joel MStation Sektorfeld instrument (Peabody, MA, USA) at a core temperature of 250 °C and 70 eV for EI or a Thermo Finnigan LTQ FT Ultra Fourier Transform Ion Cyclotron Resonance device (Thermo Fisher Scientific, Waltham, MA, USA) at 250 °C for ESI. IR spectra were recorded on a Perkin Elmer FT-IR Paragon 1000 instrument (Perkin Elmer, Hong Kong, China) as neat materials. The absorption bands were reported in wave number (cm^−1^) with ATR PRO450-S. Melting points were determined by the open tube capillary method on a Büchi melting point B-540 apparatus and are uncorrected. The HPLC purities were determined using an HP Agilent 1100 HPLC (Agilent, Santa Clara, CA, USA) with a diode array detector at 210 nm and an Agilent Poroshell column (120 EC-C18; 3.0 × 100 mm; 2.7 micron) with acetonitrile/water as eluent. Values for specific rotation (α) were measured at 23 °C at a wavelength of λ = 589 nm (Na-D-line) using a Perkin Elmer 241 Polarimeter instrument (Perkin Elmer, Hong Kong, China). All samples were dissolved in dichloromethane (layer thickness *l* = 10 cm, concentration c = 0.1 mg/100 mL). All chemicals used were of analytical grade. Isohexane, ethyl acetate and methylene chloride were purified by distillation. All reactions were monitored by thin-layer chromatography (TLC) using pre-coated plastic sheets, POLYGRAM^®^ SIL G/UV254 from Macherey-Nagel (Düren, Germany). Flash column chromatography was performed on Merck silica gel Si 60 (0.015–0.040 mm). Ozonolysis was performed on an Ozonova Type OG700-10WC (Jeske Ozontechnik, Ruderserg, Germany).

*3-((3S,3aS*,*5aS*,*6R*,*9aR*,*9bS)-3-Hydroxy-3a-methyl-7-oxododecahydro-1H-cyclopenta[a]naphthalen-6-yl)propanoic acid* (**4**): To a solution of 19-nortestosterone (**3**; 3.01 g, 10.9 mmol, 1.00 eq) in 60 mL of *tert*-butanol were added 19.5 mL of a saturated aqueous Na_2_CO_3_ solution. The mixture was heated at reflux, and a solution of NaIO_4_ (23.4 g, 110 mmol, 10.00 eq) and KMnO_4_ (0.130 g, 0.821 mmol, 7.50 mol%) in water (66 mL), preheated to 80 °C, was added via a dropping funnel over a time period of 30 min. After cooling, the reaction mixture was filtered, and the filter cake was washed with 10 mL of water. The filtrate was acidified with 6M HCI to pH 2 and then extracted with dichloromethane (4 × 20 mL). The organic phase was washed with water (20 mL) and dried over anhydrous sodium sulfate. After filtration and removal of the solvent, the crude product was purified by flash column chromatography (isohexane/ethyl acetate 1:1) to yield a colorless oil (3.08 g, 10.4 mmol, 95.4%). ^1^H NMR (400 MHz, DMSO-*d*_6_) *δ*/ppm = 11.95 (s, 1H, COOH), 4.48 (d, *J* = 4.8 Hz, 1H, OH), 3.46 (td, *J* = 8,5 Hz, 4,7 Hz, 1H, 17-H), 2.42 (m, 1H, 6-H_a_), 2.29 (ddd, *J* = 11.2 Hz, 7.8 Hz, 2.5 Hz, 1H, 10-H), 2.21 (m, 1H, 6-H_b_), 2.17 (m, 1H, 2-H_a_), 2.09 (m, 1H, 2-H_b_), 1.88 (m, 1H, 7-H_a_), 1.75 (m, 1H, 1-H_a_), 1.72 (m, 1H, 12-H_a_), 1.69 (m, 1H, 15-H_a_), 1.63 (m, 1H, 1-H_b_), 1.57 (m, 1H, 8-H), 1.52 (m, 1H, 11-H_a_), 1.36 (m, 1H, 16-H_a_), 1.30 (m, 1H, 15-H_b_), 1.24 (m, 1H, 11-H_b_ or 16-H_b_), 1.22 (m, 1H, 11-H_b_ or 16-H_b_), 1.15 (m, 1H, 7-H_b_), 1.04 (m, 1H, 9-H), 1.00 (m, 1H, 12-H_b_), 0.95 (m, 1H, 14-H), 0.72 (s, 3H, 18-H) ^13^C NMR (101 MHz, DMSO-*d*_6_) *δ*/ppm = 211.41 (C-5), 174.6 (C-3), 79.84 (C-17), 52.85 (C-10), 49.08 (C-14), 47.50 (C-9), 42.69 (C-13), 41.19 (C-6), 40.19 (C-8), 36.16 (C-12), 30.96 (C-2), 30.77 (C-7), 29.73 (C-16), 26.59 (C-15), 22.96 (C-11), 20.57 (C-1), 11.25 (C-18) IR (ATR): ν_max_/cm^−1^ = 2921, 2359, 1698, 1636, 1455, 1385, 1261, 1127, 1055, 805, 696 HRMS (EI): *m*/*z* = [M^•+^] calculated for C_17_H_26_O_4_^•+^: 294.1826; found: 294.1825.

*(4bS,6aS,7S,9aS,9bR)-7-Hydroxy-6a-methyl-1,3,4,4a,4b,5,6,6a,7,8,9,9a,9b,10-tetradecahydro-2H-indeno[5,4-f]quinolin-2-one* (**5a**) and *(4bS,6aS,7S,9aS,9bR)-7-hydroxy-6a-methyl-1,3,4,4b,5,6,6a,7,8,9,9a,9b,10,11-tetradecahydro-2H-indeno[5,4-f]quinolin-2-one* (**5b**): A mixture of compound **4** (0.795 g, 2.70 mmol, 1.00 eq) and ammonium acetate (0.728 g, 9.45 mmol, 3.50 eq) in glacial acetic acid (20 mL) was stirred and heated at reflux for 4 h. After cooling, the mixture was concentrated under reduced pressure and the residue was poured into water. The precipitate was collected by filtration, washed with water (10 mL) and dissolved in dichloromethane (20 mL). The resulting solution was washed with NaOH (1M, 3 × 10 mL), water (10 mL) and brine (10 mL), filtered over a hydrophobic filter, and concentrated in vacuo. The crude product was purified by flash column chromatography (isohexane/ethyl acetate 3:1) to yield 0.650 g (2.36 mmol, 87.4%) of a mixture of lactams **5a** and **5b** (ratio **5a**/**5b**: 15:85) as a beige solid.

**5a**: m.p.: 209 °C ^1^H NMR (500 MHz, CD_2_Cl_2_) *δ*/ppm = 6.88 (s, 1H, NH), 3.65 (t, *J* = 8.5 Hz, 1H, 17-H), 2.38 (m, 2H, 2-H), 2.32 (m, 1H, 1-H_a_), 2.20 (m, 1H, 1-H_b_), 2.15 (m, 1H, 6-H_a_), 2.04 (m, 1H, 16-H_a_), 1.95 (m, 1H, 6-H_b_), 1.87 (m, 1H, 11-H_a_ or 15-H_a_), 1.81 (dt, *J* = 12.3 Hz, 3.1 Hz, 1H, 12-H_a_), 1.75 (m, 1H, 7-H_a_), 1.71 (m, 1H, 9-H), 1.60 (m, 1H, 11-H_a_ or 15-H_a_), 1.43 (m, 1H, 16-H_b_), 1.34 (m, 1H, 8-H or 11-H_b_ or 15-H_b_), 1.31 (m, 1H, 8-H or 11-H_b_ or 15-H_b_), 1.26 (m, 1H, 11-H_b_ or 15-H_b_), 1.22 (m, 1H, 7-H_b_), 1.17 (m, 1H, 12-H_b_), 1.11 (m, 1H, 14-H_b_), 0.75 (s, 3H, 18-H) ^13^C NMR (126 MHz, CD_2_Cl_2_) *δ*/ppm = 171.03 (C-3), 128.63 (C-5), 112.88 (C-10), 81.99 (C-17), 49.70 (C-14), 44.31 (C-9), 44.00 (C-13), 39.50 (C-8), 37.16 (C-12), 31.11 (C-2), 30.92 (C-16), 27.34 (C-6), 26.33 (C-7), 25.68 (C-11 or C-15), 23.30 (C-11 or C-15), 22.21 (C-1), 11.46 (C-18) IR (ATR): ν_max_/cm^−1^ = 3465, 2913, 2868, 1683, 1668, 1542, 1507, 1473, 1456, 1388, 1319, 1284, 1224, 1186, 1133, 1055, 1027, 894, 842 HRMS (EI): *m*/*z* = [M^•+^] calculated for C_17_H_25_NO_2_^•+^: 275.1880; found: 275.1880.

**5b**: m.p.: 218 °C ^1^H NMR (500 MHz, CD_2_Cl_2_) *δ*/ppm = 7.34 (s, 1H, NH), 4.86 (dt, *J* = 5.1 Hz, 2.3 Hz, 1H, 6-H), 3.63 (t, *J* = 8.6 Hz, 1H, 17-H), 2.47 (m, 1H, 2-H_a_), 2.37 (m, 1H, 2-H_b_), 2.10 (m, 1H, 7-H_a_), 2.05 (m, 1H, 16-H_a_), 2.02 (m, 1H, 10-H), 1.92 (m, 1H, 1-H_a_), 1.81 (dt, *J* = 12.6 Hz, 3.4 Hz, 1H, 12-H_a_), 1.65 (m, 1H, 11-H_a_), 1.60 (m, 1H, 15-H_a_), 1.46 (m, 1H, 7-H_b_ or 11-H_b_), 1.43 (m, 1H, 16-H_b_), 1.40 (m, 1H, 8-H), 1.31 (m, 1H, 1-H_b_), 1.29 (m, 1H, 15-H_b_), 1.26 (m, 1H, 7-H_b_ or 11-H_b_), 1.15 (m, 1H, 12-H_b_), 1.04 (m, 1H, 14-H), 1.00 (m, 1H, 9-H), 0.76 (s, 3H, 18-H) ^13^C NMR (126 MHz, CD_2_Cl_2_) *δ*/ppm = 169.77 (C-3), 136.46 (C-5), 102.48 (C-6), 82.05 (C-17), 50.71 (C-14), 43.79 (C-9), 43.35 (C-13), 39.98 (C-10), 37.08 (C-8), 36.66 (C-12), 32.36 (C-2), 30.78 (C-16), 29.18 (C-11), 26.60 (C-1), 25.29 (C-7), 23.47 (C-15), 11.10 (C-18) IR (ATR): ν_max_/cm^−1^ = 2920, 2308, 1636, 1558, 1541, 1507, 1457, 1386, 1055, 735 HRMS (EI): *m*/*z* = [M^•+^] calculated for C_17_H_25_NO_2_^•+^: 275.1880; found: 275.1880.

*(8R,9S,13S,14S,17S)-17-(Benzyloxy)-13-methyl-1,2,6,7,8,9,10,11,12,13,14,15,16,17-tetradecahydro-3H-cyclopenta[a]phenanthren-3-one* (**7**): 19-Nortestosterone (**3**; 2.47 g, 9.00 mmol, 1.00 eq), 2-benzyloxy-1-methylpyridinium triflate (6.29 g, 18.0 mmol, 2.00 eq) and magnesium oxide (vacuum-dried, 0.744 g, 18.0 mmol, 2.00 eq) were combined in a round bottom flask. Benzotrifluoride (20 mL) was added, and the resulting suspension was heated under stirring at 83 °C for 24 h. After cooling to room temperature, the reaction mixture was diluted with dichloromethane (20 mL) and filtered through Celite. After evaporation of the solvent, the crude product was purified by flash column chromatography (isohexane/ethyl acetate 10:1) to yield 2.69 g (7.39 mmol, 82.1%) of compound **7** as a white solid. m.p.: 176 °C ^1^H NMR (400 MHz, DMSO-*d*_6_) *δ*/ppm = 7.31 (m, 4H, benzyl aromatic ortho and meta Hs), 7.26 (m, 1H, benzyl aromatic para H), 5.72 (s, 1H, 4-H), 4.50 (s, 2H, benzyl CH_2_), 3.41 (t, *J* = 8.2 Hz, 1H, 17-H), 2.42 (m, 6-H_a_), 2.26 (m, 6-H_b_), 2.22 (m, 1H, 1-H_a_), 2.20 (m, 1H, 2-H_a_), 2.16 (m, 1H, 10-H), 1.97 (m, 1H, 16-H_a_), 1.87 (dt, *J* = 12.2 Hz, 3.2 Hz, 1H, 2-H_b_), 1.78 (m, 1H, 15-H_a_), 1.73 (m, 1H, 7-H_a_), 1.55 (m, 1H, 11-H_a_), 1.48 (m, 1H, 16-H_b_), 1.43 (m, 1H, 12-H_a_), 1.37 (m, 1H, 1-H_b_ or 15-H_b_), 1.30 (m, 1H, 8-H), 1.26 (m, 1H, 11-H_b_), 1.21 (m, 1H, 1-H_b_ or 15-H_b_), 1.12 (m, 1H, 12-H_b_), 1.01 (m, 1H, 14-H), 0.95 (m, 1H, 7-H_b_), 0.81 (s, 3H, 18-H), 0.77 (m, 1H, 9-H) ^13^C NMR (101 MHz, DMSO-*d*_6_) *δ*/ppm = 198.41 (C-3), 166.76 (C-5), 139.18 (benzyl, qu_a_ternary carbon), 128.15 (benzyl, aromatic para), 127.15 (4C, benzyl aromatic ortho and meta), 123.75 (C-4), 87.59 (C-17), 70.74 (benzyl CH_2_), 49.27 (C-14), 48.95 (C-9), 42.69 (C-13), 41.67 (C-10), 37.09 (C-12), 36.16 (C-2), 34.60 (C-6), 30.28 (C-7), 27.49 (C-16), 26.13 (C-1 or C-15), 25.64 (C-1 or C-15), 22.81 (C-11), 11.68 (C-18). IR (ATR): ν_max_/cm^−1^ = 2927, 2870, 2350, 2307, 1717, 1653, 1558, 1541, 1507, 1489, 1473, 1456, 1388, 1339, 1067 HRMS (EI): *m*/*z* = [M^•+^] calculated for C_25_H_32_O_2_^•+^ 364.2397; found: 364.2396.

*3-((3S*,*3aS*,*5aS*,*9aR*,*9bS)-3-(Benzyloxy)-3a-methyl-7-oxododecahydro-1H-cyclopenta[a]naphthalen-6-yl)propanoic acid* (**8**): To a solution of compound **7** (2.63 g, 7.20 mmol, 1.00 eq) in 75 mL of *tert*-butanol were added 13.5 mL of a saturated aqueous Na_2_CO_3_ solution. The mixture was heated at reflux, and a solution of NaIO_4_ (15.4 g, 72.0 mmol, 10.00 eq) and KMnO_4_ (85.3 mg, 0.540 mmol, 7.50 mol%) in water (45 mL), preheated to 80 °C, was added via a dropping funnel over a time period of 30 min. After cooling, the reaction mixture was filtered, and the filter cake was washed with 10 mL of water. The filtrate was acidified with 6M HCl to pH 2 and then extracted with dichloromethane (4 × 20 mL). The organic phase was washed with water (20 mL) and dried over anhydrous sodium sulfate. After filtration and removal of the solvent, the crude product was purified by flash column chromatography (isohexane/ethyl acetate 3:1) to yield compound **8** as a colorless oil (2.86 g, 7.44 mmol, 99.2%) ^1^H NMR (500 MHz, CD_2_Cl_2_) *δ*/ppm = 7.33 (2s, 4H, benzyl aromatic ortho and meta Hs) 7.26 (hept, *J* = 3.8 Hz, 1H, benzyl aromatic para H), 4.51 (s, 2H, benzyl CH_2_), 3.45 (td, *J* = 7.5 Hz, 6.8 Hz, 2.5 Hz, 1H, 17-H), 2.41 (m, 1H, 2-H_a_), 2.37 (m, 1H, 6-H_a_), 2.35 (m, 1H, 6-H_b_), 2.30 (m, 1H, 2-H_b_), 2.26 (m, 1H, 7-H_a_), 2.23 (m, 1H, 10-H), 2.05 (m, 1H, 16-H_a_), 2.01 (m, 1H, 7-H_b_), 1.96 (m, 1H, 12-H_a_), 1.91 (m, 1H, 1-H_a_), 1.80 (m, 1H, 15-H_a_), 1.77 (m, 1H, 1-H_b_), 1.62 (m, 1H, 11-H_a_), 1.59 (m, 1H, 8-H), 1.55 (m, 1H, 16-H_b_), 1.39 (m, 1H, 15-H_b_), 1.35 (m, 1H, 11-H_b_), 1.20 (m, 1H, 12-H_b_), 1.08 (m, 1H, 9-H), 1.03 (m, 1H, 14-H), 0.90 (s, 3H, 18-H) ^13^C NMR (126 MHz, CD_2_Cl_2_) *δ*/ppm = 212.32 (C-5), 178.14 (C-3), 139.84 (benzyl, quaternary carbon), 128.57 (benzyl, aromatic para), 127.74 (4C, benzyl aromatic ortho and meta), 88.66 (C-17), 72.00 (benzyl CH_2_), 54.17 (C-10), 50.17 (C-14), 48.71 (C-9), 43.59 (C-13), 42.21 (C-6), 40.72 (C-8), 37.81 (C-12), 31.76 (C-7), 31.34 (C-2), 28.17 (C-16), 27.51 (C-15), 23.64 (C-11), 21.08 (C-1), 11.98 (C-18). IR (ATR): ν_max_/cm^−1^ = 2927, 2871, 2349, 2307, 1868, 1705, 1653, 1558, 1541, 1521, 1507, 1497, 1456, 1418, 1362, 1279, 869 HRMS (EI): *m*/*z* = [M^•+^] calculated for C_24_H_32_O_4_^•+^: 384.2295; found: 384.2294.

*(4bS,6aS,7S,9aS,9bR)-7-(Benzyloxy)-6a-methyl-1,3,4,4a,4b,5,6,6a,7,8,9,9a,9b,10-tetradecahydro-2H-indeno[5,4-f]quinolin-2-one* (**9a**) and *(4bS,6aS,7S,9aS,9bR)-7-(benzyloxy)-6a-methyl-1,3,4,4b,5,6,6a,7,8,9,9a,9b,10,11-tetradecahydro-2H-indeno[5,4-f]quinolin-2-one* (**9b**): A mixture of compound **8** (2.54 g, 6.60 mmol, 1.00 eq) and ammonium acetate (1.78 g, 23.1 mmol, 3.50 eq) in glacial acetic acid (60 mL) was stirred and heated at reflux for 4 h. After cooling, it was concentrated under reduced pressure and the remaining residue was poured into water. The precipitate was filtered, washed with water (20 mL) and dissolved in dichloromethane (40 mL). The resulting solution was washed with NaOH (1M, 3 × 20 mL), water (20 mL) and brine (20 mL), filtered over a hydrophobic filter and concentrated in vacuo. The crude product was purified by flash column chromatography (isohexane/ethyl acetate 5:1) to provide a total of 1.77 g (4.85 mmol, 73.4%) of fractions containing compounds **9a**/**9b** (ratio **9a**:**9b**: ca. 15:85) as beige solids (pure **9a**: 0.150 g, 0.420 mmol, 6.3%, pure **9b**: 0.870 g, 2.37 mmol, 35.9%, mixed fraction: 0.750 g, 2.05 mmol, 31.1%; ratio **9a**:**9b**: ca. 15:85).

**9a**: m.p.: 207 °C ^1^H NMR (400 MHz, CD_2_Cl_2_) *δ*/ppm = 7.32 (m, 4H, benzyl aromatic ortho and meta Hs), 7.26 (m, 1H, benzyl aromatic para H), 6.69 (s, 1H, NH), 4.51 (s, 2H, benzyl CH_2_), 3.47 (m, 1H, 17-H), 2.39 (m, 2H, 2-H), 2.32 (m, 1H, 1-H_a_), 2.20 (m, 1H, 1-H_b_), 2.14 (m, 1H, 6-H_a_), 2.04 (m, 1H, 16-H_a_), 1.98 (m, 1H, 12-H_a_), 1.92 (m, 1H, 6-H_b_), 1.85 (m, 1H, 7-H_a_), 1.75 (m, 1H, 11-H_a_), 1.69 (m, 1H, 9-H), 1.63 (m, 1H, 15-H_a_), 1.55 (m, 1H, 16-H_b_), 1.34 (m, 1H, 15-H_b_), 1.30 (m, 1H, 8-H), 1.26 (m, 1H, 12-H_b_), 1.24 (m, 1H, 11-H_b_), 1.20 (m, 1H, 7-H_b_), 1.14 (m, 1H, 14-H), 0.84 (s, 3H, 18-H) ^13^C NMR (101 MHz, CD_2_Cl_2_) *δ*/ppm = 170.75 (C-3), 139.95 (benzyl, quaternary carbon), 128.61 (C-5), 128.56 (2C, benzyl aromatic ortho or meta), 127.72 (2C, benzyl aromatic ortho or meta), 127.60 (benzyl, aromatic para), 112.82 (C-10), 88.88 (C-17), 72.00 (benzyl CH_2_), 49.88 (C-14), 44.31 (C-9), 44.18 (C-13), 39.29 (C-8), 38.36 (C-12), 31.17 (C-2), 28.39 (C-16), 27.38 (C-6), 26.33 (C-11), 25.81 (C-7), 23.32 (C-15), 22.24 (C-1), 12.21 (C-18). IR (ATR): ν_max_/cm^−1^ = 3087, 2925, 2870, 2348, 2307, 1868, 1698, 1558, 1542, 1521, 1507, 1490, 1455, 1387, 1338 HRMS (EI): *m*/*z* = [M^•+^] calculated for C_24_H_31_NO_2_^•+^: 365.2349; found: 365.2354.

**9b**: 207 °C ^1^H NMR (400 MHz, CD_2_Cl_2_) *δ* 7.52 (s, 1H, NH), 7.33 (2s, 4H, benzyl aromatic ortho and meta Hs), 7.26 (ddt, *J* = 5.7 Hz, 3.7 Hz, 2.2 Hz, 1H, benzyl aromatic para H), 4.87 (dt, *J* = 5.1 Hz, 2.3 Hz, 1H, 6-H), 4.52 (s. 2H, benzyl CH_2_), 3.45 (dd, *J* = 8.7 Hz, 7.6 Hz, 1H, 17-H), 2.47 (ddd, *J* = 17.8 Hz, 5.2 Hz, 2.0 Hz, 1H, 2-H_a_), 2.35 (ddd, *J* = 18.0 Hz, 13.0 Hz, 5.9 Hz, 1H, 2-H_b_), 2.12 (m, 1H, 7-H_a_), 2.07 (m, 1H, 7-H_b_), 2.03 (m, 1H, 16-H_a_), 2.00 (m, 1H, 10-H), 1.97 (m, 1H, 12-H_a_), 1.92 (m, 1H, 11-H_a_ or 15-H_a_), 1.58 (m, 1H, 1-H_a_), 1.55 (m, 1H, 16-H_b_), 1.48 (m, 1H, 8-H), 1.42 (m, 1H, 11-H_a_ or 15-H_a_), 1.32 (m, 1H, 1-H_b_), 1.29 (m, 1H, 12-H_b_), 1.25 (m, 1H, 11-H_b_ or 15-H_b_), 1.22 (m, 1H, 11-H_b_ or 15-H_b_), 1.05 (m, 1H, 14-H), 1.01 (m, 1H, 9-H), 0.85 (s, 3H, 18-H) ^13^C NMR (101 MHz, CD_2_Cl_2_) *δ*/ppm = 169.91 (C-3), 139.93 (benzyl, quaternary carbon), 136.45 (C-5), 128.56 (2C, benzyl aromatic ortho or meta), 127.73 (2C, benzyl aromatic ortho or meta), 127.61 (benzyl, aromatic para), 102.56 (C-6), 88.92 (C-17), 72.02 (benzyl CH_2_), 50.89 (C-14), 43.81 (C-9), 43.53 (C-13), 39.97 (C-10), 37.86 (C-12), 36.87 (C-8), 32.35 (C-2), 29.20 (C-7), 28.23 (C-16), 26.70 (C-15), 25.27 (C-11), 23.51 (C-1), 11.85 (C-18). IR (ATR): ν_max_/cm^−1^ = 3195, 3062, 2920, 2872, 1716, 1569, 1355, 1332, 1317, 1190, 1139, 1070, 1045, 843, 800, 737, 695, 647 HRMS (EI): *m*/*z* = [M^•+^] calculated For C_24_H_31_NO_2_^•+^: 365.2349; found: 365.2349.

*(4bS,6aS,7S,9aS,9bR)-7-(Benzyloxy)-6a-methyl-1,4b,5,6,6a,7,8,9,9a,9b,10,11-dodecahydro-2H-indeno[5,4-f]quinolin-2-one* (**10**) and *(4bS,6aS,7S,9aS,9bR)-7-hydroxy-6a-methyl-1,4b,5,6,6a,7,8,9,9a,9b,10,11-dodecahydro-2H-indeno[5,4-f]quinolin-2-one* (**6**): A mixture of compounds **9a**/**9b** (0.256 g, 0.700 mmol, 1.00 eq) was suspended in acetonitrile (2 mL). 1.3-Dimethoxybenzene (0.2 mL), a suspension of copper (II) bromide (87.6 mg, 0.392 mmol, 0.560 eq) and lithium bromide (78.6 mg, 0.896 mmol, 1.28 eq) in acetonitrile (2 mL) and a solution of methanesulfonic acid (23.2 µL, 0.350 mmol, 0.500 eq in 1.2 mL acetonitrile) were added. The resulting mixture was stirred and heated at reflux for 5 h. Then water (5 mL) was added, and the mixture was extracted with dichloromethane (3 × 10 mL). The organic layers were combined, washed with brine (10 mL), and filtered through a hydrophobic filter. After evaporation of the solvent, the crude product was purified by flash column chromatography to yield 86.0 mg (0.237 mmol, 33.8%) of **10** (eluated first with dichloromethane/methanol 100:3) as a beige solid and 14.9 mg (0.0546 mmol, 7.8%) of **6** (eluated second with dichloromethane/methanol 10:1) as a beige solid.

**10:** m.p.: 302 °C ^1^H NMR (400 MHz, CD_2_Cl_2_) *δ*/ppm = 12.78 (br s, 1H, NH), 7.44 (d, *J* = 9.4 Hz, 1H, 1-H), 7.33 (m, 4H, benzyl aromatic ortho and meta Hs), 7.27 (dq, *J* = 7.4, 2.8 Hz, 1H, benzyl aromatic para H), 6.28 (d, *J* = 9.3 Hz, 1H, 2-H), 4.53 (s, 2H, benzyl CH_2_), 3.50 (t, *J* = 8.2 Hz, 1H, 17-H), 2.70 (m, 2H, 6-H), 2.14 (m, 1H, 11-H_a_), 2.08 (m, 1H, 16-H_a_), 2.05 (m, 1H, 12-H_a_), 2.02 (m, 1H, 9-H), 1.89 (m, 1H, 7-H_a_), 1.68 (ddd, *J* = 12.4 Hz, 6.8 Hz, 2.8 Hz, 1H, 15-H_a_), 1.57 (ddd, *J* = 13.4 Hz, 7.8 Hz, 3.0 Hz, 1H, 16-H_b_), 1.43 (m, 1H, 8-H), 1.39 (m, 1H, 11-H_b_), 1.36 (m, 1H, 15-H_b_), 1.34 (m, 1H, 12-H_b_), 1.29 (m, 1H, 7-H_b_), 1.19 (td, *J* = 11.4 Hz, 6.9 Hz, 1H, 14-H), 0.85 (s, 3H, 18-H). ^13^C NMR (101 MHz, CD_2_Cl_2_) *δ*/ppm = 164.69 (C-3), 143.70 (C-5), 140.66 (C-1), 139.91 (benzyl, quaternary carbon), 128.57 (2C, benzyl aromatic ortho or meta), 127.73 (2C, benzyl aromatic ortho or meta), 127.62 (benzyl, aromatic para), 118.06 (C-10), 116.73 (C-2), 88.82 (C-17), 72.02 (benzyl CH_2_), 49.85 (C-14), 43.91 (C-13), 42.35 (C-9), 38.74 (C-8), 38.01 (C-12), 28.30 (C-16), 27.46 (C-6), 26.53 (C-11), 26.07 (C-7), 23.35 (C-15), 12.05 (C-18) IR (ATR): ν_max_/cm^−1^ = 3087, 2933, 2869, 2348, 2307, 1869, 1845, 1716, 1614, 1542, 1522, 1508, 1496, 1456, 1420 HRMS (EI): *m*/*z* = [M^•+^] calculated for C_24_H_29_NO_2_^•+^: 363.2193; found: 363.2192.

**6:** m.p.: 306 °C ^1^H NMR (500 MHz, MeOD-d_4_) *δ*/ppm = 7.61 (d, *J* = 9.4 Hz, 1H, 1-H), 6.36 (d, *J* = 9.3 Hz, 1H, 2-H), 3.66 (m, 1H, 17-H), 2.70 (m, 2H, 6-H), 2.24 (dq, *J* = 12.7 Hz, 3.7 Hz, 1H, 11-H_a_), 2.09 (m, 1H, 9-H), 2.04 (m, 1H, 16-H_a_), 1.97 (m, 1H, 12-H_a_), 1.93 (m, 1H, 7-H_a_), 1.69 (dddd, *J* = 12.3 Hz, 9.6 Hz, 7.1 Hz, 3.3 Hz, 1H, 15-H_a_), 1.53 (dddd, *J* = 13.1 Hz, 11.6 Hz, 8.2 Hz, 3.3 Hz, 1H, 16-H_b_), 1.44 (m, 1H, 8-H), 1.40 (m, 1H, 11-H_b_ or 15-H_b_), 1.38 (m, 1H, 11-H_b_ or 15-H_b_), 1.35 (m, 1H, 7-H_b_), 1.28 (m, 1H, 12-H_b_), 1.22 (m, 1H, 14-H), 0.79 (s, 3H, 18-H) ^13^C NMR (126 MHz, MeOD-d_4_) *δ*/ppm = 165.20 (C-3), 144.55 (C-5), 142.44 (C-1), 120.32 (C-10), 117.12 (C-2), 82.28 (C-17), 50.50 (C-14), 44.51 (C-13), 43.27 (C-9), 39.87 (C-8), 37.72 (C-12), 30.65 (C-16), 27.92 (C-6), 27.13 (C-11), 26.77 (C-7), 23.86 (C-15), 11.70 (C-18) IR (ATR): ν_max_/cm^−1^ = 3399, 2929, 2869, 1651, 1606, 1550, 1507, 1449, 1375, 1338, 1293, 1253, 1196, 1136, 1100, 1081, 1057, 1022, 960 HRMS (EI): *m*/*z* = [M^•+^] calculated for C_17_H_23_NO_2_^•+^: 273.1723; found: 273.1724.

*(4bS*,*6aS*,*7S*,*9aS*,*9bR)-7-(Benzyloxy)-2-methoxy-6a-methyl-4b*,*6,6a*,*7*,*8*,*9*,*9a*,*9b*,*10*,*11-decahydro-5H-indeno[5*,*4-f]quinoline* (**11**): To a solution of compound **10** (83.6 mg, 0.230 mmol, 1.00 eq) in chloroform (3.5 mL), silver carbonate (320 mg, 1.15 mmol, 5.00 eq) and iodomethane (0.859 mL, 13.8 mmol, 60.0 eq) were added and the mixture was stirred for 40 h at ambient temperature under exclusion of light. Thereafter, the mixture was filtered through Celite, which was washed with chloroform (5 mL), and the filtrate was concentrated in vacuo. The crude product was purified by flash column chromatography (isohexane/ethyl acetate 4:1) to yield 41.0 mg (0.109 mmol, 47.2%) of compound **11** as a colorless solid. m.p.: 102 °C ^1^H NMR (500 MHz, CD_2_Cl_2_) *δ*/ppm = 7.48 (d, *J* = 8.5 Hz, 1H, 1-H), 7.34 (m, 4H, benzyl aromatic ortho and meta Hs), 7.26 (ddt, *J* = 8.6 Hz, 5.5 Hz, 2.5 Hz, 1H, benzyl aromatic para H), 6.49 (d, *J* = 8.5 Hz, 1H, 2-H), 4.54 (benzyl CH_2_), 3.84 (OCH_3_), 3.52 (t, *J* = 8.3 Hz, 1H, 17-H), 2.84 (m, 2H, 6-H), 2.23 (m, 1H, 11-H_a_), 2.18 (m, 1H, 9-H), 2.10 (m, 1H, 16-H_a_), 2.06 (m, 1H, 12-H_a_), 1.95 (dtd, *J* = 10.6 Hz, 4.6 Hz, 2.2 Hz, 1H, 7-H_a_), 1.71 (dddd, *J* = 12.4 Hz, 9.7 Hz, 7.0 Hz, 3.3 Hz, 1H, 15-H_a_), 1.59 (dddd, *J* = 13.2 Hz, 11.5 Hz, 7.9 Hz, 3.4 Hz, 1H, 16-H_b_), 1.49 (m, 1H, 11-H_b_), 1.44 (m, 1H, 8-H), 1.41 (m, 1H, 15-H_b_), 1.39 (m, 1H, 7-H_b_), 1.36 (m, 1H, 12-H_b_), 1.22 (m, 1H, 14-H), 0.85 (s, 3H, 18-H) ^13^C NMR (126 MHz, CD_2_Cl_2_) *δ*/ppm = 161.97 (C-3), 154.62 (C-5), 139.96 (benzyl, quaternary carbon), 136.57 (C-1), 128.57 (2C, benzyl aromatic ortho or meta), 128.21 (C-10), 127.74 (2C, benzyl aromatic ortho or meta), 127.61 62 (benzyl, aromatic para), 107.59 (C-2), 88.93 (C-17), 72.02 (benzyl CH_2_), 53.32 (OCH_3_), 50.36 (C-14), 43.81 (C-13), 43.67 (C-9), 38.70 (C-8), 38.17 (C-12), 32.94 (C-6), 28.33 (C-16), 27.42 (C-7), 26.77 (C-11), 23.45 (C-15), 12.00 (C-18). IR (ATR): ν_max_/cm^−1^ = 2928, 2871, 2348, 2306, 1869, 1716, 1698, 1670, 1654, 1596, 1558, 1541, 1507, 1474, 1457, 1419 HRMS (EI): *m*/*z* = [M^•+^] calculated for C_25_H_31_NO_2_^•+^: 377.2349; found: 377.2354.

*(4bS*,*6aS*,*7S*,*9aS*,*9bR)-2-Methoxy-6a-methyl-4b*,*6*,*6a*,*7*,*8*,*9*,*9a*,*9b*,*10*,*11-decahydro-5H-indeno[5*,*4-f]quinolin-7-ol* (**1**): Under a nitrogen atmosphere compound **11** (18.9 mg, 0.0500 mmol, 1.00 eq) was dissolved in dichloromethane (1.0 mL) and cooled to −78 °C. Then, boron trichloride solution (1M in dichloromethane, 0.15 mL, 0.150 mmol, 3.00 eq) was added dropwise, and the resulting solution was allowed to warm to 0 °C and stirred at this temperature for 2 h. Thereafter, the mixture was quenched with methanol (1 mL) and filtered through Celite. After evaporation of the solvent, the crude product was purified by flash column chromatography (isohexane/ethyl acetate 3:1 with 1% triethylamine) to yield 13.0 mg (0.0452 mmol, 90.5%) of compound **1** as a white solid m.p. 157 °C ${\left[\alpha \right]}_{D}^{23}$ = 2.5° (CH_2_Cl_2_) ^1^H NMR (400 MHz, CD_2_Cl_2_) *δ*/ppm = 7.48 (d, *J* = 8.5 Hz, 1H, 1-H), 6.49 (d, *J* = 8.5 Hz, 1H, 2-H), 3.84 (s, 3H, OCH_3_), 3.69 (t, *J* = 8.4 Hz, 1H, 17-H), 2.85 (td, *J* = 7.0 Hz, 5.8 Hz, 2.6 Hz, 2H, 6-H), 2.25 (m, 1H, 11-H_a_), 2.20 (m, 1H, 9-H), 2.08 (m, 1H, 16-H_a_), 1.95 (m, 1H, 7-H_a_), 1.92 (m, 1H, 12-H_a_), 1.71 (m, 1H, 15-H_a_), 1.49 (m, 1H, 11-H_b_), 1.44 (m, 1H, 1H, 16-H_b_), 1.42 (m, 1H, 1H, 8-H), 1.38 (m, 1H, 7-H_b_), 1.34 (m, 1H, 15-H_b_), 1.26 (m, 1H, 12-H_b_), 1.19 (m, 1H, 14-H), 0.76 (s, 3H, 18-H) ^13^C NMR (101 MHz, CD_2_Cl_2_) *δ*/ppm = 161.98 (C-3), 154.63 (C-5), 136.59 (C-1), 128.20 (C-10), 107.59 (C-2), 82.08 (C-17), 53.35 (OCH_3_), 50.20 (C-14), 43.68 (C-13), 43.65 (C-9), 38.93 (C-8), 37.00 (C-12), 32.93 (C-6), 30.92 (C-16), 27.43 (C-7), 26.67 (C-11), 23.42 (C-15), 11.25 (C-18). IR (ATR): ν_max_/cm^−1^ = 2928, 2870, 2349, 2307, 1715, 1654, 1596, 1542, 1507, 1475, 1420, 1385, 1309, 1286, 1257, 1309, 1257, 1080 HRMS (EI): *m*/*z* = [M^•+^] calculated for C_18_H_25_NO_2_^•+^: 287.1880; found: 287.1888 Purity (HPLC, acetonitrile/water 70:30): >95% (λ = 210 nm), >95% (λ = 254 nm).

*Methyl 3-((3S*,*3aS*,*5aS*,*9aR*,*9bS)-3-Hydroxy-3a-methyl-7-oxododecahydro-1H-cyclopenta[a]naphthalen-6-yl)propanoate* (**12**): Ketocarboxylic acid **4** (3.06 g, 10.4 mmol, 1.00 eq), Cs_2_CO_3_ (6.78 g, 20.8 mmol, 2.00 eq) and dry DMF (34 mL) were added to an oven-dried round-bottom flask and the mixture was stirred for 30 min at room temperature. Then, iodomethane (2.37 mL, 15.6 mmol, 1.50 eq) was added, and the reaction mixture was stirred overnight at room temperature. After quenching with H_2_O (30 mL), the mixture was extracted with diethyl ether (3 × 30 mL). The combined organic phases were washed with water (30 mL) and brine (30 mL), dried over anhydrous sodium sulfate, filtered and concentrated in vacuo. The residue was purified by flash column chromatography (isohexane/ethyl acetate 2:1) to obtain methyl ester **12** as a colorless oil (3.11 g, 10.1 mmol, 96.9%) ^1^H NMR (400 MHz, DMSO-*d*_6_) *δ*/ppm = 4.49 (d, 1H, *J* = 4.9 Hz, OH), 3.45 (td, 1H, *J* = 8.5 Hz, 4.9 Hz, 17-H), 2.41 (m, H, 6-H_a_), 2.29 (m, 1H, 10-H), 2.24 (m, 1H, 6-H_b_), 2.20 (m, 1H, 2-H_a_), 2.13 (m, 1H, 2-H_b_), 1.88 (m, 1H, 7-H_a_), 1.81 (m, 1H, 1-H_a_), 1.73 (m, 1H, 12-H_a_), 1.68 (m, 1H, 15-H_a_), 1.64 (m, 1H, 1-H_b_), 1.58 (m, 1H, 8-H), 1.51 (m, 1H, 11-H_a_), 1.37 (m, 1H, 16-H_a_), 1.30 (m, 1H, 15-H_b_), 1.24 (m, 1H, 16-H_b_), 1.19 (m, 1H, 11-H_b_), 1.12 (m, 1H, 7-H_b_), 1.04 (m, 1H, 9-H), 0.99 (m, 1H, 12-H_b_), 0.93 (m, 1H, 14-H), 0.71 (s, 3H, 18-H) ^13^C NMR (101 MHz, DMSO-*d*_6_): *δ*/ppm = 211.39 (C-5), 173.49 (C-3), 79.86 (C-17), 52.75 (C-10), 51.22 (OCH_3_), 49.09 (C-14), 47.41 (C-9), 42.70 (C-13), 41.17 (C-6), 40.19 (C-8), 36.17 (C-12), 30.75 (C-2), 30.66 (C-7), 29.73 (C-16), 26.58 (C-15), 22.97 (C-11), 20.58 (C-1), 11.25 (C-18). IR (ATR): ν_max_/cm^−1^ = 2943, 2308, 1733, 1715, 1647, 1542, 1457, 1387, 1055 HRMS (EI): *m*/*z* = [M^•+^] calculated for C_18_H_28_O_4_^•+^: 308.1982; found: 308.1982.

*Methyl 3-((3S,3aS,5aS,6R,9aR,9bS)-3-Hydroxy-3a-methyldodecahydrospiro-[cyclopenta[a]naphthalene-7,2′-[1,3]dioxolan]-6-yl)propanoate* (**13**): A mixture of ketone **12** (3.09 g, 10.0 mmol, 1.00 eq), trimethyl orthoformate (24.1 mL, 220.0 mmol, 22.0 eq), ethylene glycol (24 mL, 430 mmol, 43.0 eq) and p-toluenesulfonic acid (0.194 g, 1.00 mmol, 0.100 eq) in a round bottom flask was stirred overnight at room temperature. The mixture was diluted with ethyl acetate, and the solution was washed with saturated aqueous sodium bicarbonate solution. The organic phase was dried over anhydrous sodium sulfate, filtered, and concentrated. The residue was purified by flash column chromatography (isohexane/ethyl acetate 2:1) to obtain the dioxolane **13** as a colorless solid (2.38 g, 6.75 mmol, 67.5%). m.p.: 84 °C ^1^H NMR (400 MHz, DMSO-*d*_6_) *δ*/ppm = 4.44 (d, 1H, *J* = 4.8 Hz, OH), 3.88 (m, 2H, ethylene), 3.81 (m, 2H, ethylene), 3.56 (2s, 3H, OCH_3_) 3.43 (td, 1H, *J* = 8.4 Hz, 4.9 Hz, 17-H), 2.38 (ddd, *J* = 16.7 Hz, 10.0 Hz, 6.6 Hz, 1H, 2-H_a_), 2.24 (ddd, *J* = 16.0 Hz, 10.0 Hz, 6.1 Hz, 1H, 2-H_b_), 1.82 (m, 1H, 16-H_a_), 1.76 (m, 1H, 6-H_a_), 1.70 (m, 1H, 12-H_a_), 1.64 (m, 1H, 15-H_a_), 1.61 (m, 1H, 1-H_a_), 1.57 (m, 1H, 1-H_b_), 1.47 (m, 1H, 11-H_a_ or 7-H_a_), 1.44 (m, 1H, 11-H_a_ or 7-H_a_), 1.38 (m, 1H, 10-H), 1.31 (m, 1H, 16-H_b_), 1.21 (m, 1H, 6-H_b_), 1.16 (m, 1H, 11-H_b_ or 15-H_b_), 1.13 (m, 1H, 11-H_b_ or 15-H_b_), 1.06 (m, 1H, 8-H), 1.01 (m, 1H, 9-H or 12-H_b_), 0.97 (m, 1H, 9-H or 12-H_b_), 0.95 (m, 1H, 7-H_b_), 0.91 (m, 1H, 14-H), 0.63 (s, 3H, 18-H) ^13^C NMR (101 MHz, DMSO-*d*_6_) *δ*/ppm = 173.76 (C-3), 110.55 (C-5), 79.99 (C-17), 63.88 (ethylene), 63.86 (ethylene), 51.14 (OCH_3_), 49.41 (C-14), 47.32 (C-10), 44.90 (C-9), 42.71 (C-13), 40.22 (C-8), 36.58 (C-12), 33.91 (C-6), 32.68 (C-2), 29.82 (C-16), 27.17 (C-7), 25.92 (C-15), 22.93 (C-11), 21.13 (C-1), 11.35 (C-18). IR (ATR): ν_max_/cm^−1^ = 1868, 2307, 1732, 1698, 1647, 1635, 1321 HRMS (EI): *m*/*z* = [M^•+^] calculated for C_20_H_32_O_5_^•+^: 352.2244; found: 352.2244.

*Methyl 3-((3S,3aS,5aS,6R,9aR,9bS)-3-((tert-butyldimethylsilyl)oxy)-3a-methyl dodecahydrospiro[cyclopenta[a]naphthalene-7,2′-[1,3]dioxolan]-6-yl)propanoate* (**14**): Compound **13** (2.38 g, 6.75 mmol, 1.00 eq) was dissolved in dimethylformamide (14 mL). Then imidazole (0.957 g, 14.1 mmol, 3.80 eq) and tert-butyldimethylsilyl chloride (1.06 g, 7.03 mmol, 2.00 eq) were added, and the resulting mixture was stirred overnight at room temperature. After addition of water (10 mL), the mixture was extracted with ethyl acetate (3 × 10 mL). The combined organic layers were washed with 1M hydrochloric acid (30 mL), water (20 mL) and brine (20 mL), dried over anhydrous sodium sulfate, filtered and concentrated in vacuo. The crude product was purified by flash column chromatography (isohexane/ethyl acetate 9:1) to obtain a colorless solid (2.35 g, 5.02 mmol, 74.4%). m.p.: 86 °C ^1^H NMR (400 MHz, CD_2_Cl_2_) *δ*/ppm = 3.94 (m, 2H, ethylene), 3.89 (m, 2H, ethylene), 3.61 (s, 3H, ester CH_3_), 3.58 (m, 1H, 17-H), 2.43 (m, 1H, 2-H_a_), 2.31 (m, 1H, 2-H_b_), 1.88 (m, 1H, 12-H_a_), 1.80 (m, 1H, 6-H_a_), 1.74 (m, 1H, 7-H_a_), 1.71 (m, 1H, 1-H_a_), 1.69 (m, 1H, 10-H), 1.56 (m, 1H, 16-H_a_), 1.54 (m, 1H, 11-H_a_ or 15-H_a_), 1.51 (m, 1H, 11-H_a_ or 15-H_a_), 1.44 (m, 1H, 12-H_b_), 1.39 (m, 1H, 16-H_b_), 1.29 (m, 1H, 6-H_b_), 1.25 (m, 1H, 1-H_b_), 1.21 (m, 1H, 11-H_b_), 1.14 (m, 1H, 15-H_b_), 1.09 (m, 1H, 8-H or 9-H), 1.06 (m, 1H, 8-H or 9-H), 1.02 (m, 1H, 7-H_b_), 0.97 (m, 1H, 14-H), 0.87 (s, 9H, tert-butyl), 0.72 (s, 3H, 18-H), 0.01 (s, 3H, dimethylsilyl), 0.01 (s, 3H, dimethylsilyl). ^13^C NMR (101 MHz, CD_2_Cl_2_) *δ*/ppm = 175.01 (ester carbonyl), 111.91 (C-5), 82.36 (C-17), 64.89 (ethylene), 51.69 (methyl ester), 49.96 (C-14), 48.53 (C-10), 45.93 (C-9), 43.99 (C-13), 41.33 (C-8), 37.70 (C-12), 34.93 (C-6), 33.69 (C-2), 31.44 (C-16), 27.97 (C-7), 27.04 (C-15), 26.18 (tert-butyl CH_3_), 23.89 (C-11), 21.96 (C-1), 18.54 (tert-butyl C), 11.77 (C-18), −4.25 (dimethylsilyl), −4.54 (dimethylsilyl). IR (ATR): ν_max_/cm^−1^ = 2926, 2885, 2854, 2307, 1735, 1472, 1162, 1093, 899, 885 HRMS (EI): *m*/*z* = [M^•+^] calculated for C_26_H_46_O_5_Si^•+^: 466.3109; found: 466.3102.

*Methyl (E)-3-((3S,3aS,5aS,6R,9aR,9bS)-3-((tert-butyldimethylsilyl)oxy)-3a-methyldodecahydrospiro[cyclopenta[a]naphthalene-7,2′-[1,3]dioxolan]-6 yl)acrylate* (**15**): Dry THF (1.2 mL) and lithium diisoproylamide (2M in in THF, 3.76 mL, 7.53 mmol, 1.25 eq) were added to a flame-dried Schlenk flask under nitrogen. The solution was cooled down to -78 °C, and after 10 min, a solution of compound **14** (2.34 g, 5.02 mmol, 1.00 eq) in 8.5 mL of dry THF was added dropwise via a syringe. After stirring for 25 min, a solution of diphenyldiselenide (0.888 g, 2.84 mmol, 1.25 eq) in 8.8 mL dry THF was added quickly. The mixture was stirred at −78 °C for 30 min and then gradually warmed up to room temperature over a 2 h period. The reaction mixture was then quenched by adding a saturated ammonium chloride solution (50 mL). After extraction with ethyl acetate (3 × 5 mL), the combined organic layers were washed with 1M hydrochloric acid (50 mL), water (50 mL), saturated aqueous sodium bicarbonate solution (50 mL) and brine (50 mL), dried over anhydrous sodium sulfate and filtered. After the evaporation of the solvent, an orange solid was obtained. Dichloromethane (15 mL) was added to this solid, and the resulting solution was cooled to 0 °C. The temperature of the solution was monitored throughout the whole reaction. Then, a solution of hydrogen peroxide (30%, 4.4 mL, 131 mmol, 26.0 eq) in water (4.4 mL) was added dropwise. After the addition was complete, the temperature of the reaction mixture rose quickly to about 30 °C, dropping thereafter. The mixture was allowed to come to room temperature and stirred until the reaction was complete (TLC control). The reaction mixture was transferred to a separation funnel containing saturated aqueous sodium bicarbonate solution (50 mL) and extracted with dichloromethane (3 × 25 mL). The combined organic layers were dried over anhydrous sodium sulfate, filtered and concentrated in vacuo. The crude product was purified by flash column chromatography (isohexane/ethyl acetate 9:1) to yield compound **15** (1.69 g, 3.63 mmol, 72.2%) as a colorless solid. m.p.: 96 °C ^1^H NMR (400 MHz, CD_2_Cl_2_) *δ*/ppm = 6.72 (dd, 1H, *J* = 15.7 Hz, 10.0 Hz, 1-H), 5.82 (d, 1H, *J* = 15.7 Hz, 2-H), 3.90-3.70 (m, 4H, ethylene), 3.68 (s, 3H, ester CH_3_), 3.58 (dd, 1H, *J* = 8.8 Hz, 7.8 Hz, 17-H), 2.21 (t, *J* = 10.6 Hz, 1H, 10-H), 1.89 (dtd, *J* = 13.1 Hz, 9.1 Hz, 5.7 Hz, 1H, 16-H_a_), 1.79 (dt, *J* = 13.5 Hz, 3.0 Hz, 1H, 6-H_a_), 1.66 (dt, *J* = 12.2 Hz, 2.9 Hz, 1H, 12-H_a_), 1.60 (m, 1H, 7-H or 11-H), 1.56 (m, 1H, 15-H_a_), 1.45 (m, 1H, 6-H_b_), 1.41 (m, 1H, 16-H_b_), 1.38 (m, 1H, 7-H or 11-H), 1.30 (m, 1H, 7-H or 11-H), 1.26 (m, 1H, 15-H_b_), 1.24 (m, 1H, 9-H), 1.12 (m, 1H, 8-H), 1.08 (m, 1H, 7-H or 11-H), 1.02 (m, 1H, 12-H_b_), 0.98 (m, 1H, 14-H), 0.87 (s, 9H, tert-butyl), 0.71 (s, 3H, 18-H), 0.00 (s, 6H, dimethylsilyl) ^13^C NMR (101 MHz, CD_2_Cl_2_) *δ*/ppm = 167.04 (ester carbonyl), 148.73 (C-1), 124.24 (C-2), 110.53 (C-5), 82.26 (C-17), 65.78 (Ethylen), 65.50 (ethylene), 55.39 (C-10), 51.72 (OCH_3_), 49.86 (C-14), 45.00 (C-9), 44.15 (C-13), 40.48 (C-8), 37.38 (C-12), 35.68 (C-6), 31.37 (C-16), 28.03 (C-7 or C-11), 28.02 (C-7 or C-11), 26.17 (tert-butyl CH_3_), 23.83 (C-15), 18.52 (tert-butyl C), 11.72 (C-18), −4.25 (dimethylsilyl), −4.25 (dimethylsilyl). IR (ATR): ν_max_/cm^−1^ = 2952, 2928, 2858, 1718, 1652, 1472, 1435, 1163, 900, 772 HRMS (EI): *m*/*z* = [M^•+^] calculated for C_26_H_44_O_5_Si^•+^: 464.2953; found: 464.2951.

*(3S,3aS,5aS,9aR,9bS)-3-((tert-Butyldimethylsilyl)oxy)-3a-methyldodecahydrospiro[cyclopenta[a]naphthalene-7,2′-[1,3]dioxolane]-6-carbaldehyde* (**16**): Compound **15** (1.44 g, 3.10 mmol) was dissolved in a mixture of dichloromethane (18 mL) and methanol (10 mL). The solution was cooled to −78 °C and then treated with ozone (5 min, flow: 50 L/h, 55 W). Progress of the reaction was monitored via TLC. After excess ozone had been removed by a stream of nitrogen, dimethyl sulfide (18.0 mL, 243 mmol) was added, and the reaction mixture was allowed to warm gradually. It was then stirred overnight at room temperature. The mixture was diluted with dichloromethane (18 mL) and washed with saturated aqueous sodium bicarbonate solution (2 × 50 mL) and brine (50 mL). The organic layer was dried over anhydrous sodium sulfate filtered, and the solvent was evaporated to obtain a colorless solid (0.960 g, 2.35 mmol, 75.8%), which was used as such in the next step. m.p.: 135 °C ^1^H NMR (500 MHz, CD_2_Cl_2;_ pure compound obtained by tedious flash chromatography) *δ*/ppm = 4.01(m, 4H, ethylene), 3.61 (s, 3H, OCH_3_), 3.59 (t, *J* = 8.3 Hz, 17-H), 2.54 (d, *J* = 11.5 Hz, 1H, 10-H), 1.92 (m, 1H, 16-H_a_), 1.89 (m, 1H, 6-H_a_), 1.72 (m, 1H, 12-H_a_), 1.62 (m, 1H, 7-H_a_ or 11-H_a_), 1.56 (m, 1H, 15-H_a_), 1.49 (m, 1H, 7-H_a_ or 11-H_a_), 1.45 (m, 1H, 16-H_b_), 1,41 (m, 1H, 8-H), 1.39 (m, 1H, 6-H_b_), 1.36 (m, 1H, 7-H_b_ or 11-H_b_), 1.28 (m, 1H, 15-H_b_), 1.15 (m, 1H, 9-H), 1.14 (m, 1H, 7-H_b_ or 11-H_b_), 1.05 (m, 1H, 12-H_b_), 1.01 (m, 1H, 14-H), 0.87 (s, 9H, (CH_3_)_3_), 0.73 (s, 3H, 18-H), 0.01 (s, 3H, dimethylsilyl), 0.01 (s, 3H, dimethylsilyl) ^13^C NMR (126 MHz, CD_2_Cl_2_) *δ*/ppm = 172.18 (C-1), 109.88 (C-5), 82.11 (C-17), 64.98 (ethylen), 64.95 (ethylen), 57.16 (C-10), 49.55 (C-14), 44.20 (C-13), 44.20 (C-8) 40.33 (C-9), 37.06 (C-12), 34.11 (C-6), 31.29 (C-16), 27.58 (C-7 or C-11), 27.34 (C-7 or C-11), 26.15 (tert-butyl CH_3_), 23.75 (C-15), 18.51 (tert-butyl quaternary carbon), 11.68 (C-18), −4.26 (dimethylsilyl), −4.57 (dimethylsilyl). IR (ATR): ν_max_/cm^−1^ = 2927, 2308, 1733, 1717, 1653, 1558, 1261, 900 HRMS (EI): *m*/*z* = [M^•+^] calculated for C_23_H_40_O_4_Si^•+^: 408.2690; found: 408.2658.

*(3S*,*3aS*,*5aS*,*9aR*,*9bS)-3-Hydroxy-3a-methyl-7-oxododecahydro-1H-cyclopenta[a]naphthalene-6-carbaldehyde* (**17**): Crude compound **16** (0.899 g, about 2.20 mmol) was suspended in a mixture of glacial acetic acid (22.0 mL), THF (7.5 mL) and water (7.5 mL) and stirred overnight at room temperature. After the addition of 16.4 mL of a 50% solution of acetic acid in water, the mixture was refluxed for 1 h. After cooling to room temperature, brine (20 mL) was added, and the mixture was extracted with ethyl acetate (4 × 50 mL). The combined organic extracts were washed with saturated aqueous sodium bicarbonate solution (50 mL), dried over anhydrous sodium sulfate and filtered. After evaporation of the solvent, compound **17** was obtained as a colorless oil (0.436 g, 1.74 mmol, about 79.2%), which was used as such in the next step. ^1^H NMR (400 MHz, CD_2_Cl_2_; pure compound obtained by tedious flash chromatography) *δ*/ppm = 15.45 (d, *J* = 6.2 Hz, 0.41H, enol OH), 9.60 (dd, *J* = 4.6 Hz, 2.2 Hz, 1H, 0.26H, aldehyde, keto tautomer), 8.28 (d, *J* = 5.7 Hz, 0.46H, aldehyde, enol tautomer), 3.90 (m, 1H), 3.66 (m, 1H, 17-H), 2.46 (m, 1H), 2.04 (m, 1H), 1.97 (m, 1H), 1.84 (m, 1H), 1.68 (m, 1H), 1.61 (m, 1H), 1.57 (m, 1H), 1.45 (m, 1H), 1.40 (m, 1H), 1.36 (m, 1H), 1.31 (m, 1H), 1.27 (m, 1H), 1.20 (m, 1H), 1.15 (m, 1H), 1.09 (m, 1H), 0.80-0.73 (3s, 3H, 18-H). ^13^C NMR (101 MHz, CD_2_Cl_2_) *δ*/ppm = 204.59 (aldehyde, keto tautomer), 200.93 (C-5, keto tautomer) 194.50 (C-5, enol tautomer), 178.27 (aldehyde, enol tautomer), 113.83 (C-10, enol tautomer), 81.98 (C-17), 62.51 (C-10, keto tautomer), 49.56 (C-14), 43.58 (C-13), 41.19 (CH), 39.04 (CH), 36.52 (CH_2_), 33.95 (CH_2_), 30.69 (CH_2_), 26.25 (CH_2_), 26.17 (CH_2_), 23.38 (CH_2_), 11.20 (C-18). IR (ATR): ν_max_/cm^−1^ = 2927, 2307, 1733, 1716, 1636, 1457, 1082 HRMS (EI): *m*/*z* = [M^•+^] calculated for C_15_H_22_O_3_^•+^: 250.1563; found: 250.1563.

*(4bS*,*6aS*,*7S*,*9aS*,*9bR)-2-Methoxy-6a-methyl-4b*,*6*,*6a*,*7*,*8*,*9*,*9a*,*9b*,*10*,*11-decahydro-5H-indeno[5,4-f]quinazolin-7-ol* (**2**): Crude compound **17** (0.401 g, 1.60 mmol, 1.00 eq) was added to a round bottom flask and dissolved in 10 mL of dry methanol. Then, methyl carbamimidate sulfate (0.826 g, 4.80 mmol, 3.00 eq) and 3.2 mL of a freshly prepared solution of sodium methanolate in methanol (0.11 g of sodium in 3.2 mL of dry methanol, 4.80 mmol, 3.00 eq) were added to the flask and the mixture was refluxed for 8 h under a nitrogen atmosphere. After cooling to room temperature, water (100 mL) was added, and the mixture was extracted with ethyl acetate (3 × 50 mL). The combined organic layers were washed with water and brine and dried over anhydrous sodium sulfate. After filtration and evaporation of the solvent, the residue was purified by flash column chromatography (isohexane/ethyl acetate 2:1 with 1% triethylamine) to obtain compound **2** as a white solid (0.174 g, 0.603 mmol, 37.7%). m.p.: 179 °C ${\left[\alpha \right]}_{D}^{23}$ = 2.1° (CH_2_Cl_2_) ^1^H NMR (400 MHz, CD_2_Cl_2_) *δ*/ppm = 8.31 (s, 1H, 1-H), 3.91 (s, 3H, OCH_3_), 3.70 (t, 1H, *J* = 8.5 Hz, 17-H), 2.83 (m, 2H, 6-H), 2.29 (m, 1H, 11-H_a_), 2.20 (m, 1H, 9-H), 2.08 (m, 1H, 16-H_a_), 1.96 (m, 1H, 7-H_a_), 1.92 (m, 1H, 12-H_a_), 1.70 (m, 1H, 15-H_a_), 1.48 (m, 1H, 11-H_b_), 1.45 (m, 1H, 16-H_b_), 1.42 (m, 1H, 8-H), 1.39 (m, 1H, 7-H_b_), 1.36 (m, 1H, 15-H_b_), 1.29 (m, 1H, 12-H_b_), 1.18 (m, 1H, 14-H), 0.76 (s, 3H, 18-H) ^13^C NMR (101 MHz, CD_2_Cl_2_) *δ*/ppm = 168.87 (C-5), 164.32 (C-3), 156.38 (C-1), 127.06 (C-10), 82.14 (C-17), 54.86 (OCH_3_), 50.12 (C-14), 43.78 (C-13), 42.11 (C-9), 38.76 (C-8), 36.85 (C-12), 32.74 (C-6), 30.91 (C-16), 26.92 (C-7), 26.10 (C-11), 23.54 (C-15), 11.36 (C-18). IR (ATR): ν_max_/cm^−1^ = 2943, 2866, 2307, 1734, 1654, 1587, 1546, 1467, 1389, 1323, 1034, 749 HRMS (ESI): *m*/*z* = [M + H]^+^ calculated for C_17_H_25_N_2_O_2_^+^: 289.1911; found: 289.1912 Purity (HPLC, acetonitrile/water 50:50): >96% (λ = 210 nm), >97% (λ = 254 nm).

### 3.2. Biological Testing

hTRPML1ΔNC-YFP, a plasma membrane variant of wild-type TRPML1, was obtained from HEK293 cells stably expressing plasma membrane-targeted TRPML1 by trypsination and after resuspending in HEPES buffered solution. IC_50_ values for TRPML1 inhibition were determined on a fluorescence imaging plate reader built into a robotic liquid handling station (Freedom Evo 150, Tecan, Mannedorf, Switzerland) using the calcium dye Fluo-4/AM (Invitrogen, Thermo Fisher Scientific, Waltham, MA, USA) according to the test procedure described in our previous work [13] in the section Compound screening and generation of concentration–response curves.

## 4. Conclusions

In conclusion, we have worked out straightforward chiral pool syntheses of the 4-aza (1) and 2,4-diaza analog (**2**) of the TRPML1 inhibitor estradiol methyl ether (**EDME**) starting with oxidative cleavage of ring A of the readily available steroid 19-nortestosterone (**3**) to provide ketocarboxylic acid **4** as the central intermediate for both target compounds. By utilizing carefully selected protective groups for the 17-OH group (benzyl in the pyridine synthesis, TBDS in the pyrimidine synthesis) and oxidants for dehydrogenation (CuBr_2_/LiBr/methanesulfonic acid in the pyridine synthesis) and chain degradation (selenylation/selenoxide elimination/ozonolysis in the pyrimidine synthesis) both target compounds were obtained in 6 and 8 steps, respectively, and in acceptable overall yields (8.6%, 7.5%).

While the 4-aza analog **1** showed significant TRPML1-inhibitory activity (only factor <2 less potent than the gold standard **EDME**), the 2,4-diaza analog **2** significantly lost inhibitory potency, and the pyridone analog, obtained as an unexpected side product, was completely inactive. This leads to the conclusion that for the cation channel TRPML1, aza analogs are not promising bioisosteres of **EDME**.

## Figures and Tables

**Table 1:** TRPML1-inhibitory activities of lead structure **EDME** and the three synthesized aza analogs.

Compound	IC_50_ for TRPML1 Inhibition
EDME	0.60 µM
1	1.0 µM
2	8.8 µM
6	40 µM

## Data Availability

Experimental data, spectra, and protocols are stored in an electronic lab journal by the authors.

## Supplementary Materials

The following supporting information can be downloaded at: https://www.mdpi.com/article/10.3390/molecules28217428/s1, Additional synthetic procedures; Figure S1: Numbering of the compounds (for assignment of NMR signals); ^1^H- and ^13^C-NMR spectra of the compounds.

## References

[1] Bargal R., Avidan N., Ben-Asher E., Olender Z., Zeigler M., Frumkin A., Raas-Rothschild A., Glusman G., Lancet D., Bach G. (2000). Identification of the gene causing mucolipidosis type IV. Nat. Genet..

[2] Kasitinon S.Y., Eskiocak U., Martin M., Bezwada D., Khivansara V., Tasdogan A., Zhao Z., Mathews T., Aurora A.B., Morrison S.J. (2019). TRPML1 Promotes Protein Homeostasis in Melanoma Cells by Negatively Regulating MAPK and mTORC1 Signaling. Cell Rep..

[3] Yin C., Zhang H., Liu X., Zhang H., Zhang Y., Bai X., Wang L., Li H., Li X., Zhang S. (2019). Downregulated MCOLN1 Attenuates The Progression Of Non-Small-Cell Lung Cancer By Inhibiting Lysosome-Autophagy. Cancer Manag. Res..

[4] Xing Y., Sui Z., Liu Y., Wang M.-m., Wei X., Lu Q., Wang X., Liu N., Lu C., Chen R. (2022). Blunting TRPML1 channels protects myocardial ischemia/reperfusion injury by restoring impaired cardiomyocyte autophagy. Basic Res. Cardiol..

[5] Santoni G., Maggi F., Amantini C., Marinelli O., Nabissi M., Morelli M.B. (2020). Pathophysiological Role of Transient Receptor Potential Mucolipin Channel 1 in Calcium-Mediated Stress-Induced Neurodegenerative Diseases. Front. Physiol..

[6] Zhang X., Li X., Xu H. (2012). Phosphoinositide isoforms determine compartment-specific ion channel activity. Proc. Natl. Acad. Sci. USA.

[7] Chen C.C., Keller M., Hess M., Schiffmann R., Urban N., Wolfgardt A., Schaefer M., Bracher F., Biel M., Wahl-Schott C. (2014). A small molecule restores function to TRPML1 mutant isoforms responsible for mucolipidosis type IV. Nat. Commun..

[8] Shen D., Wang X., Li X., Zhang X., Yao Z., Dibble S., Dong X.P., Yu T., Lieberman A.P., Showalter H.D. (2012). Lipid storage disorders block lysosomal trafficking by inhibiting a TRP channel and lysosomal calcium release. Nat. Commun..

[9] Spix B., Butz E., Chen C.-C., Scotto Rosato A., Tang R., Jeridi A., Kudrina V., Melzergeb Plesch E., Wartenberg P., Arlt E. (2022). Lung emphysema and impaired macrophage elastase clearance in mucolipin 3 deficient mice. Nat. Commun..

[10] Wang W., Gao Q., Yang M., Zhang X., Yu L., Lawas M., Li X., Bryant-Genevier M., Southall N.T., Marugan J. (2015). Up-regulation of lysosomal TRPML1 channels is essential for lysosomal adaptation to nutrient starvation. Proc. Natl. Acad. Sci. USA.

[11] Leser C., Keller M., Gerndt S., Urban N., Chen C.C., Schaefer M., Grimm C., Bracher F. (2021). Chemical and pharmacological characterization of the TRPML calcium channel blockers ML-SI1 and ML-SI3. Eur. J. Med. Chem..

[12] Kriegler K., Leser C., Mayer P., Bracher F. (2022). Effective chiral pool synthesis of both enantiomers of the TRPML inhibitor trans-ML-SI3. Arch. Pharm..

[13] Rühl P., Rosato A.S., Urban N., Gerndt S., Tang R., Abrahamian C., Leser C., Sheng J., Jha A., Vollmer G. (2021). Estradiol analogs attenuate autophagy, cell migration and invasion by direct and selective inhibition of TRPML1, independent of estrogen receptors. Sci. Rep..

[14] Mayer C.D., Bracher F. (2011). Cytotoxic ring A-modified steroid analogues derived from Grundmann’s ketone. Eur. J. Med. Chem..

[15] Renard D., Perruchon J., Giera M., Müller J., Bracher F. (2009). Side chain azasteroids and thiasteroids as sterol methyltransferase inhibitors in ergosterol biosynthesis. Bioorg. Med. Chem..

[16] Meanwell N.A. (2011). Synopsis of some recent tactical application of bioisosteres in drug design. J. Med. Chem..

[17] Subbaiah M.A.M., Meanwell N.A. (2021). Bioisosteres of the Phenyl Ring: Recent Strategic Applications in Lead Optimization and Drug Design. J. Med. Chem..

[18] Tse E.G., Houston S.D., Williams C.M., Savage G.P., Rendina L.M., Hallyburton I., Anderson M., Sharma R., Walker G.S., Obach R.S. (2020). Nonclassical Phenyl Bioisosteres as Effective Replacements in a Series of Novel Open-Source Antimalarials. J. Med. Chem..

[19] Brill Z.G., Condakes M.L., Ting C.P., Maimone T.J. (2017). Navigating the Chiral Pool in the Total Synthesis of Complex Terpene Natural Products. Chem. Rev..

[20] Holt D.A., Levy M.A., Brandt M., Metcalf B.W. (1986). Inhibition of pyridine-nucleotide-dependent enzymes by pyrazoles. Synthesis and enzymology of A novel A-ring pyrazole steroid. Steroids.

[21] Rasmusson G.H., Reynolds G.F., Utne T., Jobson R.B., Primka R.L., Berman C., Brooks J.R. (1984). Azasteroids as inhibitors of rat prostatic 5 alpha-reductase. J. Med. Chem..

[22] Xia P., Yang Z.-Y., Xia Y., Zhang H.-B., Zhang K.-H., Sun X., Chen Y., Zheng Y.-Q. (1998). Synthesis of N-Substituted 3-Oxo-17β- carboxamide-4-aza-5α-androstanes and the Tautomerism of 3-Oxo-4-aza-5-androstenes. Heterocycles.

[23] Skaanderup P.R., Poulsen C.S., Hyldtoft L., Jørgensen M.R., Madsen R. (2002). Regioselective Conversion of Primary Alcohols into Iodides in Unprotected Methyl Furanosides and Pyranosides. Synthesis.

[24] Poon K.W.C., Dudley G.B. (2006). Mix-and-Heat Benzylation of Alcohols Using a Bench-Stable Pyridinium Salt. J. Org. Chem..

[25] Zhang J., Yan Y., Hu R., Li T., Bai W.J., Yang Y. (2020). Enantioselective Total Syntheses of Lyconadins A-E through a Palladium-Catalyzed Heck-Type Reaction. Angew. Chem. Int. Ed..

[26] Fischer D.F., Sarpong R. (2010). Total Synthesis of (+)-Complanadine A Using an Iridium-Catalyzed Pyridine C−H Functionalization. J. Am. Chem. Soc..

[27] Lee A.S., Liau B.B., Shair M.D. (2014). A unified strategy for the synthesis of 7-membered-ring-containing Lycopodium alkaloids. J. Am. Chem. Soc..

[28] Wu B., Bai D. (1997). The First Total Synthesis of (±)-Huperzine B. J. Org. Chem..

[29] King A.O., Anderson R.K., Shuman R.F., Karady S., Abramson N.L., Douglas A.W. (1993). Iodotrimethylsilane-mediated 2-monohalogenation of 4-aza-5.alpha.-androstan-3-one steroids. J. Org. Chem..

[30] King L.C., Ostrum G.K. (1964). Selective Bromination with Copper(II) Bromide1. J. Org. Chem..

[31] Sander M., Gries J., Schuetz A. (2009). Method for Aromatizing 19-Norandrost-4-en-3-Ones to Estra-1,3,5(10)-Trienes. U.S. Patent Application.

[32] White J.D., Li Y., Kim J., Terinek M. (2015). Cyclobutane Synthesis and Fragmentation. A Cascade Route to the Lycopodium Alkaloid (−)-Huperzine A. J. Org. Chem..

[33] Mandal P.K., McMurray J.S. (2007). Pd−C-Induced Catalytic Transfer Hydrogenation with Triethylsilane. J. Org. Chem..

[34] Falck J.R., Barma D.K., Baati R., Mioskowski C. (2001). Differential Cleavage of Arylmethyl Ethers: Reactivity of 2,6-Dimethoxybenzyl Ethers. Angew. Chem. Int. Ed..

[35] Aversa R.J., Burger M.T., Dillon M.P., Dineen T.A., Lou Y., Nishiguchi G.A., Ramurthy S., Rico A.C., Rauniyar V., Sendzik M. (2014). Compounds and Compositions as Raf Kinase Inhibitors. U.S. Patent Application.

[36] Bertin D., Nedelec L., Pierdet A. (1967). Diaza Steroids.

[37] Nomine G. (1960). Derivatives of 7-oxo-1,2,3,4,5,6,7,8-Octahydronaphthalene.

[38] de Avellar I.G.J., Vierhapper F.W. (2000). Novel Partial Synthetic Approaches to Replace Carbons 2,3,4 of Steroids. A Methodology to Label Testosterone and Progesterone with 13C in the Steroid A Ring. Part 1. Tetrahedron.

