# Supplementary material for: Aza Analogs of the TRPML1 Inhibitor Estradiol Methyl Ether (EDME)
Source: Molecules. 2023 Nov 4;28(21):7428. doi: 10.3390/molecules28217428 (PMC10647736; doi:10.3390/molecules28217428)

Supporting Information

Aza Analogs of the TRPML1 Inhibitor Estradiol Methyl Ether (EDME)

Philipp Rühl [1] and Franz Bracher [1,*]

[1] Department of Pharmacy – Center for drug Research, Ludwig-Maximilians University, Munich, Germany; philipp.ruehl@cup.lmu.de (P.R.); franz.bracher@cup.uni-muenchen.de (F.B.)

* Correspondence: franz.bracher@cup.uni-muenchen.de

Additional synthetic procedures

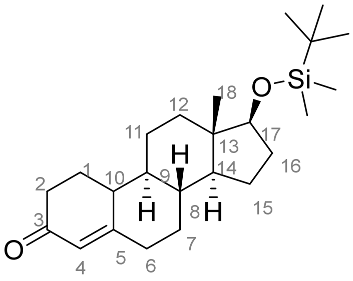

*(8R,9S,13S,14S,17S)-17-((tert-Butyldimethylsilyl)oxy)-13-methyl-1,2,6,7,8,9,10,11,12,13,14,15,16, 17-tetradecahydro-3H-cyclopenta[a]phenanthren-3-one (**S1**):* 19-Nortestosterone (**3**; 1.92 g, 7.00 mmol, 1.00 eq), *tert*-butyldimethylsilyl chloride (1.32 g, 8.75 mmol, 1.25 eq) and imidazole (1.19 g, 17.5 mmol, 2.50 eq) were combined in a round bottom flask. Dry dimethylformamide (20 mL) was added and the resulting reaction mixture stirred at room temperature for 7 h. It was diluted with diethyl ether (150 mL) and washed with 2M aqueous hydrochloric acid solution (3 x 50 mL), saturated aqueous sodium bicarbonate solution (3 x 50 mL) and water (30 mL). The organic layer was dried over anhydrous sodium sulfate and filtered. After evaporation of the solvent, the crude product was purified by flash column chromatography (isohexane/ ethyl acetate 4:1) to yield 2.60 g (6.69 mmol, 95.6 %) of the desired compound as a white solid. m.p.: 137 °C. ^{1}H NMR (400 MHz, CD_2Cl_2) δ/ppm = 5.75 (s, 1H, 4-H), 3.59 (dd, *J* = 8.8 Hz, 7.8 Hz, 1H, 17-H), 2.45 (ddd, *J* = 14.5 Hz, 4.0 Hz, 2.4 Hz, 1H, 6-H_a), 2.34 (m, 1H, 6-H_b), 2.28 (m, 1H, 2-H_a), 2.25 (m, 1H, 1-H_a), 2.22 (m. 1H, 2-H_b), 2.08 (m, 1H, 10-H), 1.90 (m, 1H, 16-H_a) 1.85 (m, 1H, 7-H_a or 15-H_a), 1.81 (m, 1H, 7-H_a or 15-H_a), 1.77 (m, 1H, 12-H_a), 1.59 (m, 1H, 11-H_a), 1.51 (m, 1H, 1-H_b), 1.45 (m, 1H, 16-H_b), 1.34 (m, 1H, 8-H), 1.29 (m, 1H, 11-H_b), 1.25 (m, 1H, 15-H_b), 1.06 (m, 1H, 12-H_b), 1.02 (m, 1H, 7-H_b), 0.97 (m, 1H, 9-H), 0.89 (s, 9H, $(CH_3)_3$), 0.82 (m, 1H, 14-H), 0.77 (s, 3H, 18-H) , 0.02 (s, 3H, dimethylsilyl$_a$), 0.01 (s, 3H, dimethylsilyl$_b$) ^{13}C NMR (101 MHz, CD_2Cl_2) δ/ppm = 199.74 (C-3), 167.24 (C-5), 124.62 (C-4), 82.11 (C-17), 50.20 (C-14), 49.79 (C-9), 43.74 (C-13), 43.00 (C-10), 40.92 (C-8), 37.28 (C-12), 36.96 (C-2), 35.90 (C-6), 31.24 (C-7 or C-16), 31.22 (C-7 or C-16), 27.10 (C-1), 26.62 (C-15), 26.02 ($(CH_3)_3$), 23.69 (C-11), 18.38 (tert-butyl, quaternary carbon), 11.50 (C-18), -4.39 (dimethylsilyl$_a$), -4.72 (dimethylsilyl$_b$) IR (ATR): ν_{max}/cm^{-1} = 2929, 2856, 1667, 1616, 1457, 1249, 1140, HRMS (ESI): m/z = $[M+H]^+$ calculated for $C_{24}H_{41}O_2Si^+$: 389.2870; found: 389.2871.

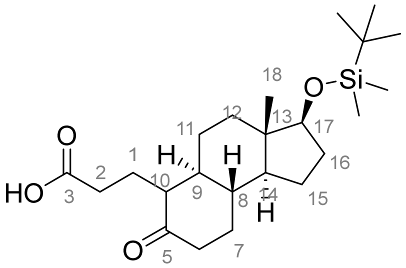

*3-((3S,3aS,5aS,6R,9aR,9bS)-3-((tert-Butyldimethylsilyl)oxy)-3a-methyl-7-oxododecahydro-1H-cyclopenta[a]naphthalen-6-yl)propanoic acid (**S2**)*: To a solution of the above compound (2.60 g, 6.70 mmol, 1.00 eq) in 40 mL of *tert*-butanol were added 13.0 mL of a saturated aqueous Na_2CO_3 solution. The mixture was heated at reflux and a solution of $NaIO_4$ (14.3 g, 67.0 mmol, 10.00 eq) and $KMnO_4$ (80.0 mg, 0.500 mmol, 7.50 mol%) in water (40 mL) preheated to 80 °C was added via a dropping funnel over a time period of 30 min. After cooling, the reaction mixture was filtered and the filter cake was washed with 10 mL of water. The filtrate was acidified with 6M HCl to pH 2 and then extracted with dichloromethane (4 x 20 mL). The organic phase was washed with water (20 mL) and dried over anhydrous sodium sulfate. After filtration and removal of the solvent, the crude product was purified by column chromatography to yield a colorless oil (2.37 g, 5.81 mmol, 86.7%) ^{1}H NMR (400 MHz, DMSO-d_6) δ/ppm = 11.98 (s, 1H, COOH), 3.58 (t, *J* = 8.1 Hz, 1H, 17-H), 2.41 (td, *J* = 13.7 Hz, 6.1 Hz, 1H, 6-H_a), 2.29 (m, 1H, 10-H), 2.20 (m, 1H, 6-H_b), 2.13 (m, 1H, 2-H_a), 2.07 (m, 1H, 2-H_b), 1.90 (m, 1H, 7-H_a or 16-H_a), 1.85 (m, 1H, 7-H_b or 16-H_a), 1.76 (m, 1H, 1-H_a), 1.69 (m, 1H, 15-H_a), 1.65 (m, 1H, 12-H_a), 1.61 (m, 1H, 1-H_b), 1.57 (m, 1H, 8-H), 1.51 (m, 1H, 11-H_a), 1.39 (m, 1H, 7H_b or 16-H_b), 1.32 (m, 1H, 15-H_b), 1.27 (m, 1H, 11-H_b), 1.15 (m, 1H, 7-H_b or 16-H_b), 1.06 (m, 1H, 12-H_b), 1.01 (m, 1H, 9-H), 0.94 (m, 1H, 14-H), 0.86 (s, 9H, $(CH_3)_3$), 0,74 (s, 3H, 18-H), 0.01 (s, 3H, dimethylsilyl$_a$), 0.00 (s, 3H, dimethylsilyl$_b$) ^{13}C NMR (101 MHz, DMSO-d_6) δ/ppm = 211.39 (C-5), 174.61 (C-3), 81.01 (C-17), 52.77 (C-10), 49.50 (C-14), 47.40 (C-9), 43.00 (C-13), 41.15 (C-6), 40.15 (C-8), 36.18 (C-12), 30.91 (C-2), 30.70 (C-7 or C-16), 30.40 (C-7 or C-16), 26.50 (C-15), 25.75 ($(CH_3)_3$), 23.01 (C-11), 20,54 (C-1), 17.78 (*tert*-butyl, quaternary carbon), 11.30 (C-18), -4.51 (dimethylsilyl$_a$), -4.84 (dimethylsilyl$_b$) IR (ATR): ν_{max}/cm^{-1} = 2927, 2856, 1715. 1558, 1541, 1418, 1249, 1199 HRMS (ESI): m/z = (M-H)$^-$ calculated for $C_{23}H_{39}O_4Si^-$: 407.2623; found: 407.2625.

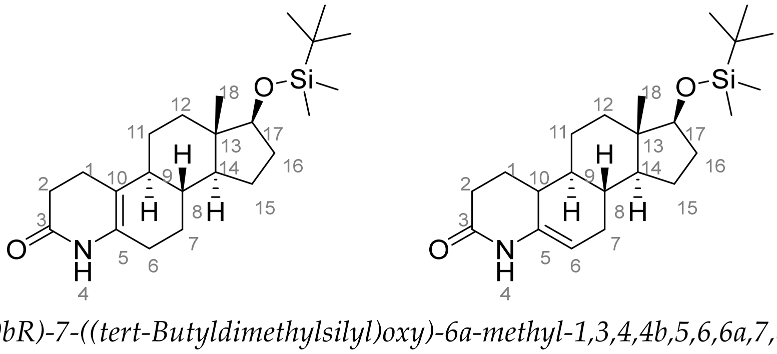

(4bS,6aS,7S,9aS,9bR)-7-((tert-Butyldimethylsilyl)oxy)-6a-methyl-1,3,4,4b,5,6,6a,7,8,9,9a,9b,10,11-tetradecahydro-2H-indeno[5,4-f]quinolin-2-one ***(Δ5,10 isomer, S3a)*** and *(4bS,6aS,7S,9aS,9bR)-7-((tert-butyldimethylsilyl)oxy)-6a-methyl-1,3,4,4a,4b,5,6,6a,7,8,9,9a,9b,10-tetradecahydro-2H-indeno[5,4-f]quinolin-2-one* ***(Δ5,6 isomer, S3b):*** A mixture of the above ketocarboxylic acid (2.32

g, 5.70 mmol, 1.00 eq) and ammonium acetate (1.54 g, 19.9 mmol, 3.50 eq) in glacial acetic acid (45 mL) was stirred and heated at reflux for 4 h. After cooling, it was concentrated under reduced pressure and the remaining residue was poured into water. The precipitate was filtered, washed with water (20 mL) and dissolved in dichloromethane (40 mL). The resulting solution was washed with NaOH (1M, 3 x 20 mL), water (20 mL) and brine (20 mL), filtered over a hydrophobic filter and concentrated in vacuo. The crude product was purified by flash column chromatography (isohexane/ ethyl acetate 5:1) to yield 1.59 g (4.08 mmol, 71.6%) of a mixture of the two lactams (ratio: Δ5,10 isomer: Δ5,6 isomer: 15:85) as a beige solid.

Δ5,10 isomer S3a: ^{1}H NMR (500 MHz, CD_2Cl_2) *δ*/ppm = 3.62 (m, 1H, 17-H), 2.38 (m, 1H, 6-H$_a$), 2.31 (m, 1H, 1-H$_a$), 2.20 (m, 1H, 2-H$_a$), 2.12 (m, 1H, 1-H$_b$), 1.95 (m, 1H, 2-H$_b$), 1.91 (m, 1H, 16-H$_a$), 1.86 (m, 1H, 15-H$_a$), 1.81 (m, 1H, 12-H$_a$), 1.74 (m, 1H, 7-H$_a$), 1.68 (m, 1H, 9-H), 1.57 (m, 1H, 11-H$_a$), 1.50 (m, 1H, 15-H$_b$), 1.43 (m, 1H, 16-H$_b$), 1.36 (m, 1H, 11-H$_b$), 1.30 (m, 1H, 8-H), 1.26 (m, 1H, 6-H$_b$), 1.19 (m, 1H, 7-H$_b$), 1.10 (m, 1H, 14-H), 1.01 (m, 1H, 12-H$_b$), 0.88 (s, 9H, $(CH_3)_3$), 0.73 (s, 3H, 18-H), 0.02 (2s, 6H, dimethylsilyl) ^{13}C NMR (126 MHz, CD_2Cl_2) *δ*/ppm = 171.05 (C-3), 128.52 (C-5), 113.11 (C-10), 82.08 (C-17), 49.32 (C-14), 44.41 (C-9), 44.38 (C-13), 39.56 (C-8), 37.60 (C-12), 31.38 (C-6 or C-16), 31.10 (C-6 or C-16), 27.39 (C-2), 26.36 (C-7), 26.01 ($(CH_3)_3$), 25.75 (C-15), 23.45 (C-11), 22.21 (C-1), 18.38 (*tert*-butyl, quaternary carbon), 11.76 (C-18), -4.39 (dimethylsilyl$_a$), -4.72 (dimethylsilyl$_b$) IR (ATR): $_{max}$/cm^{-1} = 32145, 2953, 2928, 1695, 1674, 1472, 1387, 1255, 1141, 1094, HRMS (EI): m/z = [$M^{\bullet +}$] calculated for $C_{23}H_{39}NO_2Si^{\bullet +}$: 389.2745; found: 389.2746.

Δ5,6 isomer S3b*:* ^{1}H NMR (500 MHz, CD_2Cl_2) *δ*/ppm = 7.58 (NH), 4.88 (dt, *J* = 5.1 Hz, 2.3 Hz, 1H, 6-H), 3.60 (t, *J* = 8.3 Hz, 1H, 17-H), 2.46 (ddd, *J* = 17.3 Hz, 5.0 Hz, 2.0 Hz, 1H, 2-H$_a$), 2.37 (m, 1H, 2-H$_b$), 2.11 (m, 1H, 1-H$_a$), 2.07 (m, 1H, 7-H$_a$), 1.95 (m, 1H, 10-H), 1.91 (m, 1H, 16-H$_a$), 1.89 (m, 1H, 11-H$_a$), 1.78 (m, 1H, 12-H$_a$), 1.64 (m, 1H, 7-H$_b$), 1.56 (m, 1H, 15-H$_a$), 1.44 (m, 1H, 16-H$_b$), 1.40 (m, 1H, 8-H), 1.31 (m, 1H, 1-H$_b$), 1.29 (m, 1H, 15-H$_b$, 1.27 (m, 1H, 11-H$_b$), 1.08 (m, 1H, 12-H$_b$), 1.00 (m, 1H, 9-H), 0.97 (m, 1H, 14-H), 0.88 (s, 9H, $(CH_3)_3$), 0.74 (s, 3H, 18-H), 0.02 (2s, 6H, $Si(CH_3)_2$) ^{13}C NMR (126 MHz, CD_2Cl_2) *δ*/ppm = 170.07 (C-3), 136.43 (C-5), 102.77 (C-6), 82.15 (C-17), 50.35 (C-14), 43.91 (C-9), 43.73 (C-13), 39.97 (C-10), 37.15 (C-8), 37.10 (C-12), 32.33 (C-2), 31.20 (C-16), 29.24 (C-7), 26.69 (C-11), 26.01 ($(CH_3)_3$), 25.27 (C-1), 23.63 (C-15), 18.38 (*tert*-butyl, quaternary carbon), 11.40 (C-18), -4.39 (dimethylsilyl$_a$), -4.72 (dimethylsilyl$_b$). IR (ATR): ν_{max}/cm^{-1} = 32145, 2953, 2928, 1695, 1674, 1472, 1387, 1255, 1094 HRMS (EI): m/z = [$M^{\bullet +}$] calculated for $C_{23}H_{39}NO_2Si^{\bullet +}$: 389.2745; found: 389.2745.

Figure S1: Numbering of the compounds (for assignment of NMR signals).

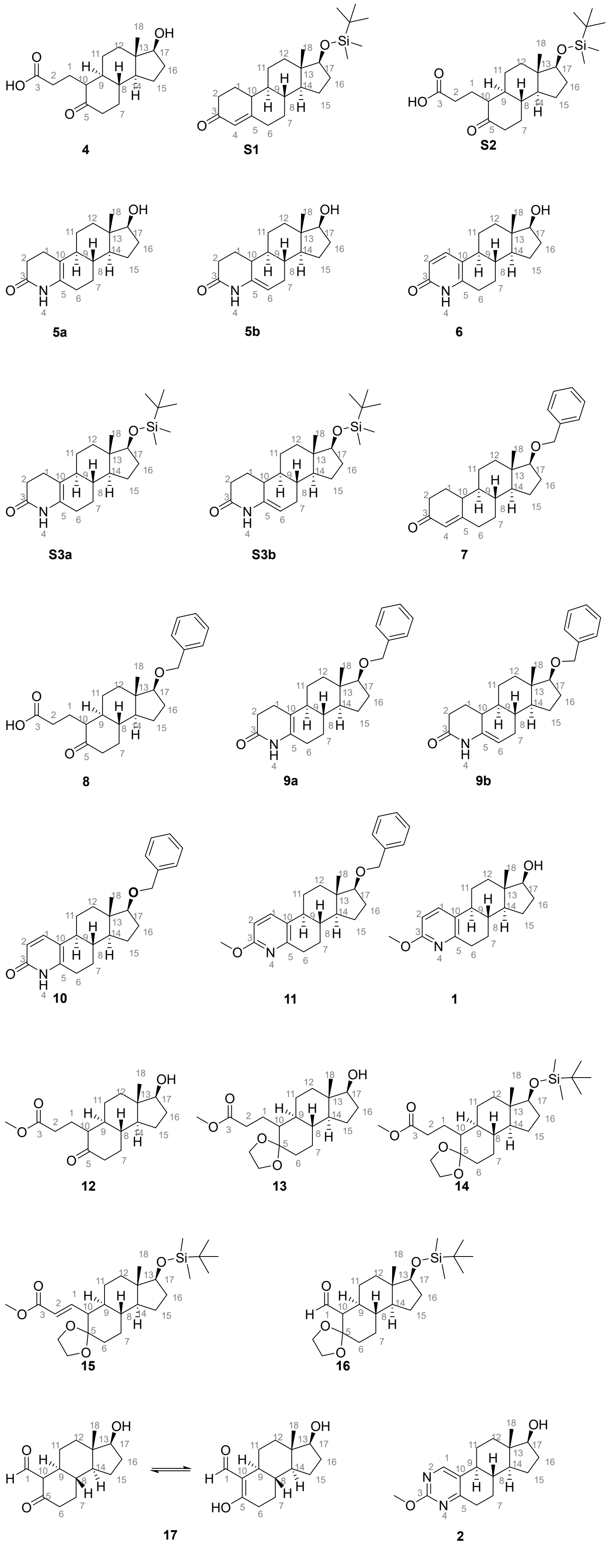

^{1}H- and ^{13}C-NMR spectra

Compound **4**

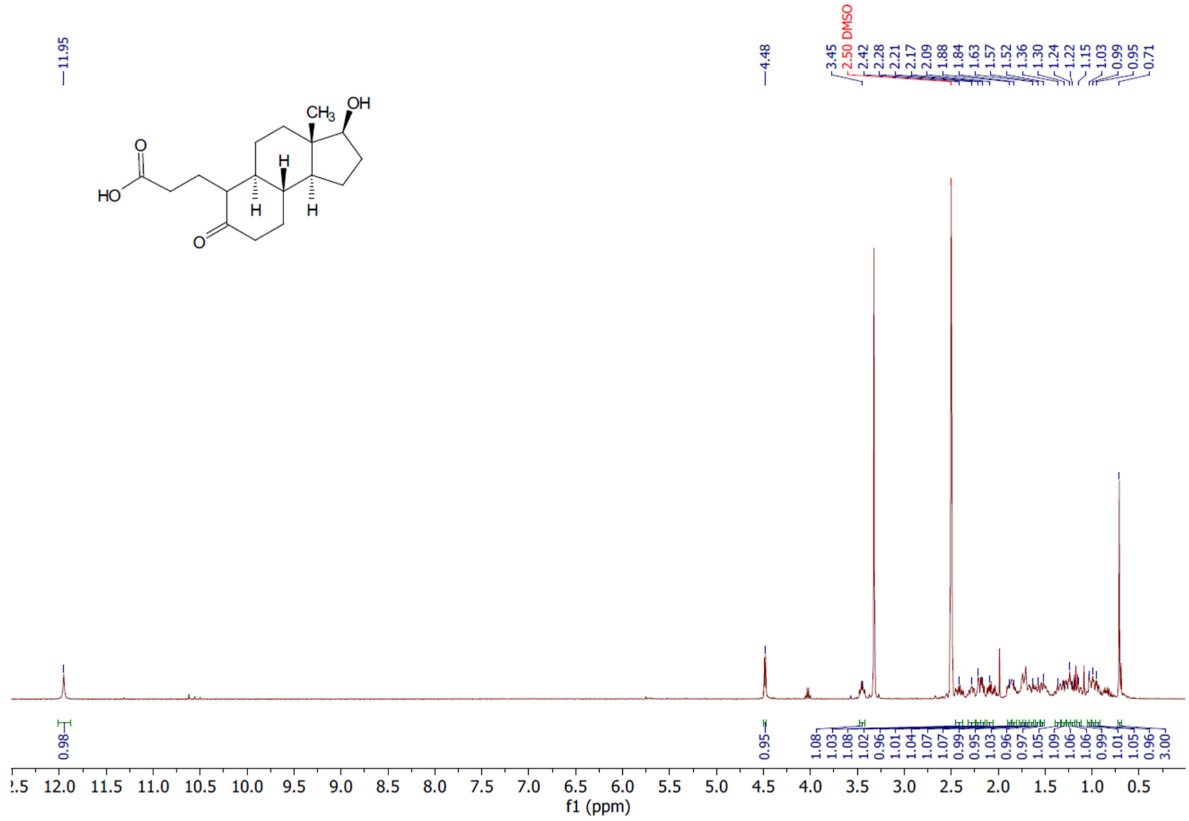

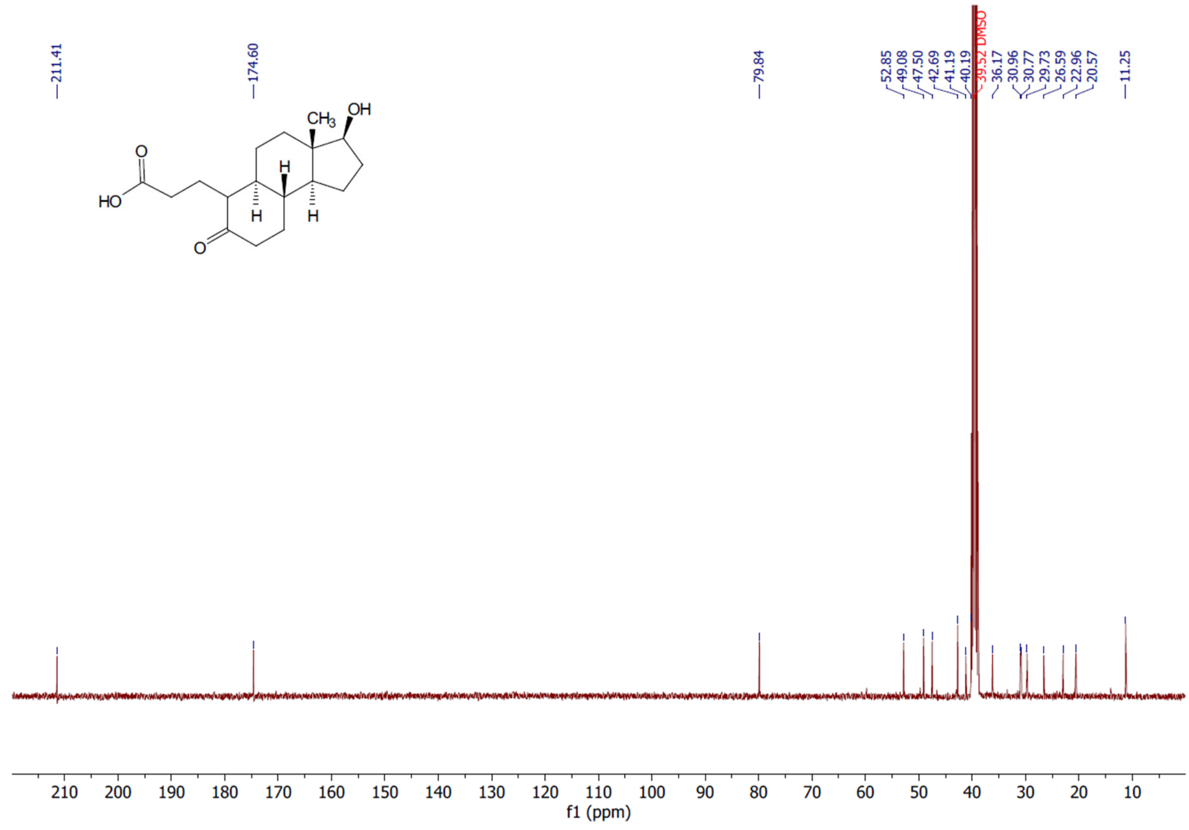

Compound 5a

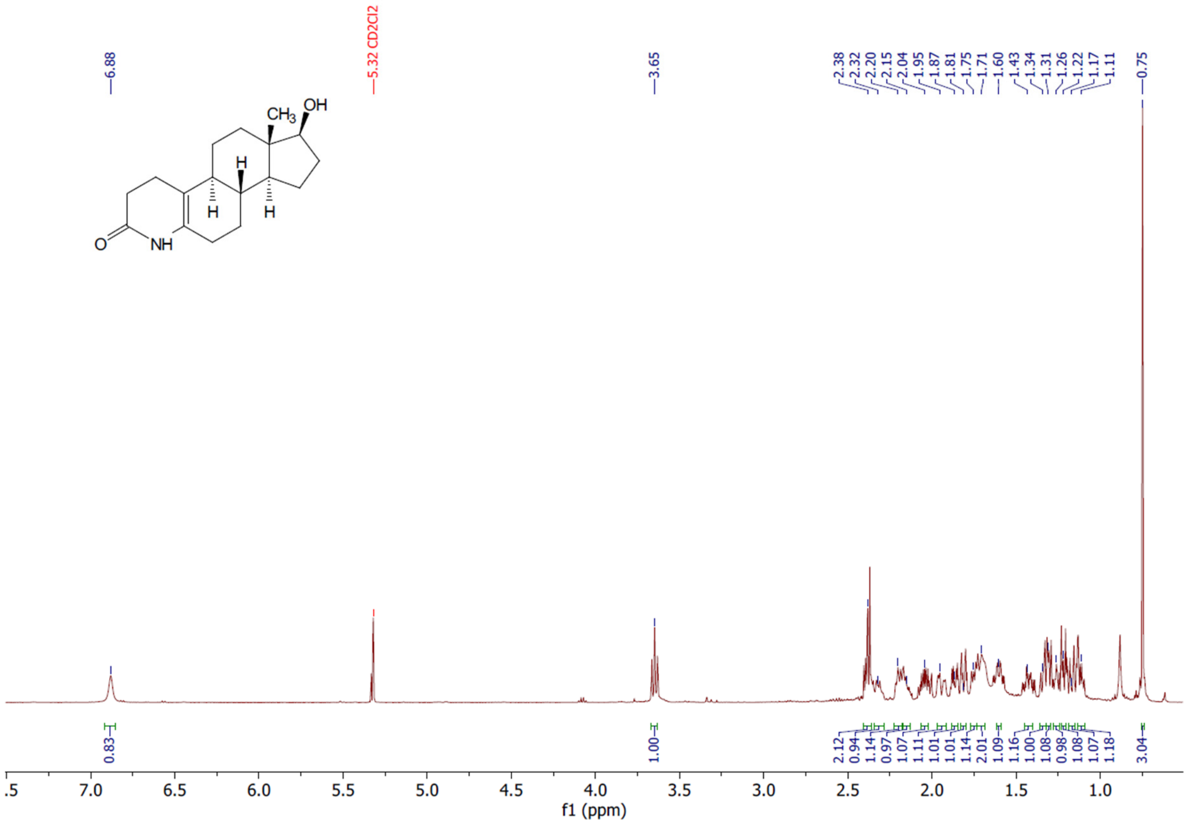

6.88
5.32 CD2Cl2
3.65
2.38
2.32
2.20
2.15
2.04
1.95
1.87
1.81
1.75
1.71
1.60
1.43
1.34
1.31
1.26
1.22
1.17
1.11
0.75
CH3
OH
H
H
H
O
NH
0.83
1.00
2.12
0.94
1.14
0.97
1.07
1.11
1.01
1.01
1.14
2.01
1.09
1.16
1.00
1.08
0.98
1.08
1.07
1.18
3.04
f1 (ppm)

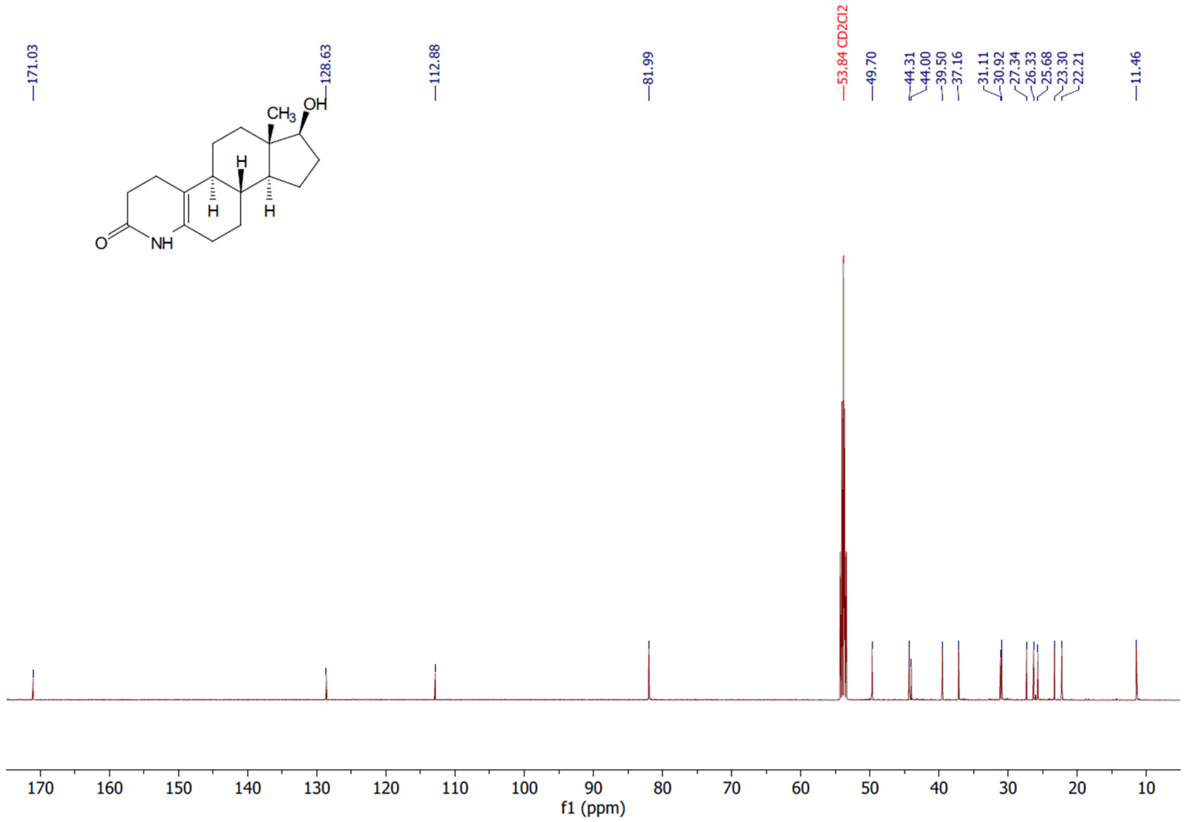

171.03
128.63
112.88
81.99
53.84 CD2Cl2
49.70
44.31
44.00
39.50
37.16
31.11
30.92
27.34
26.33
25.68
23.30
22.21
11.46
CH3
OH
H
H
H
O
NH
f1 (ppm)

Compound 5b

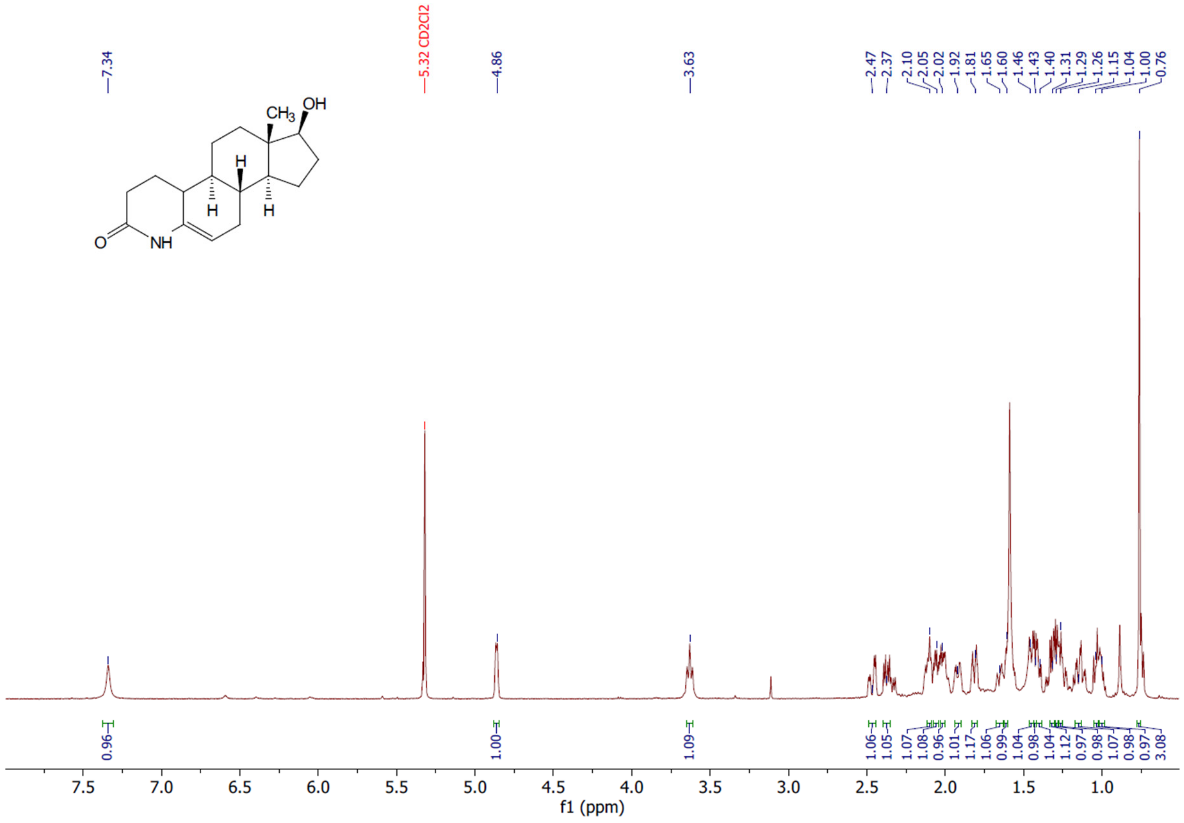

7.34
5.32 CD2Cl2
4.86
3.63
2.47
2.37
2.10
2.05
2.02
1.92
1.81
1.65
1.60
1.46
1.43
1.40
1.31
1.29
1.26
1.15
1.04
1.00
0.76
CH3
OH
H
O
NH
0.96
1.00
1.09
1.06
1.05
1.07
1.08
0.96
1.01
1.17
1.06
0.99
1.04
0.98
1.04
1.12
0.97
0.98
1.07
0.98
0.97
3.08
7.5 7.0 6.5 6.0 5.5 5.0 4.5 4.0 3.5 3.0 2.5 2.0 1.5 1.0
f1 (ppm)

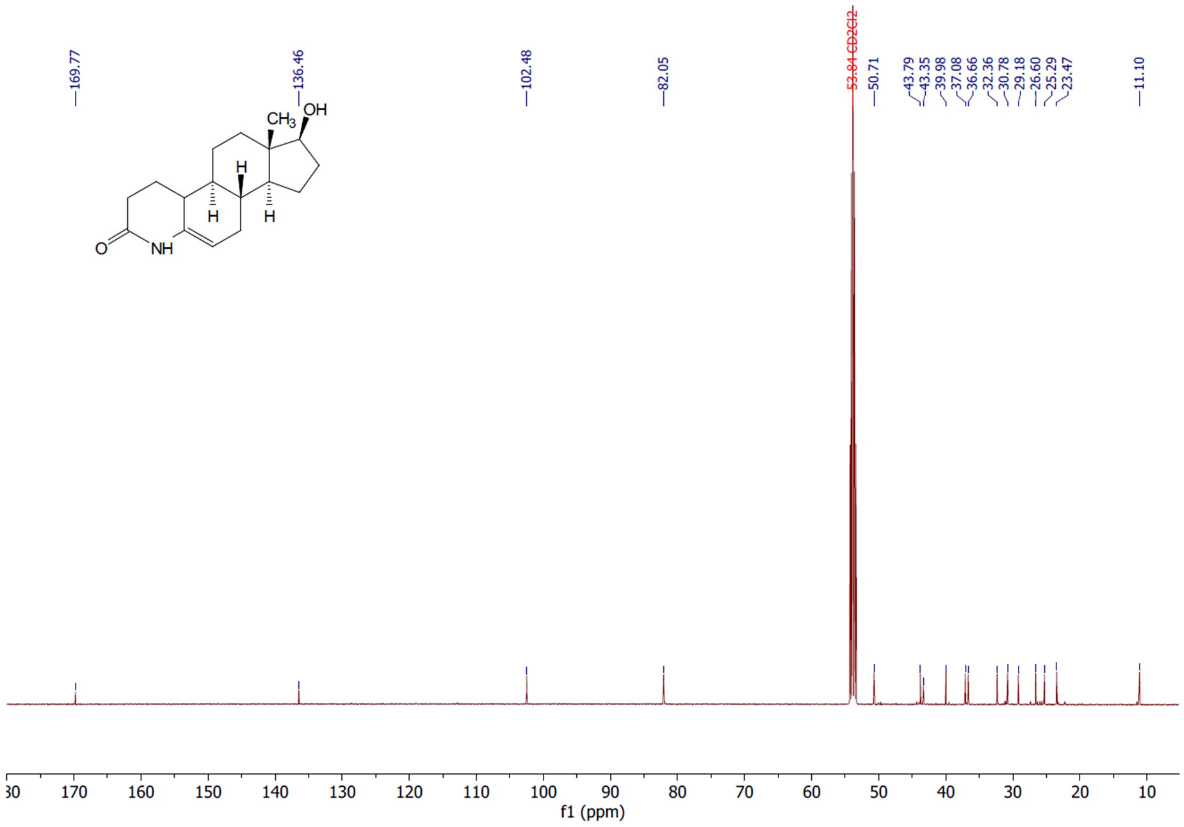

169.77
136.46
102.48
82.05
53.84 CD2Cl2
50.71
43.79
43.35
39.98
37.08
36.66
32.36
30.78
29.18
26.60
25.29
23.47
11.10
CH3
OH
H
O
NH
170 160 150 140 130 120 110 100 90 80 70 60 50 40 30 20 10
f1 (ppm)

Compound 6

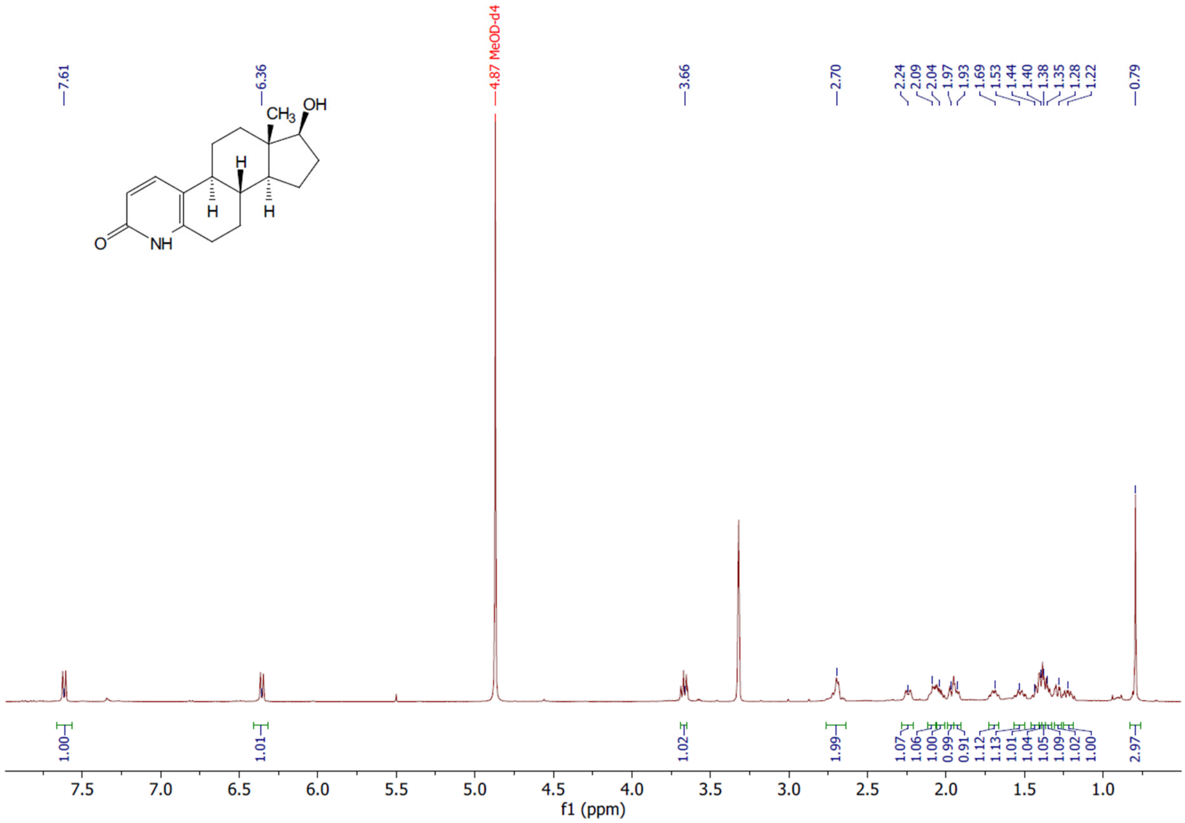

7.61
6.36
4.87 MeOD-d4
3.66
2.70
2.24
2.09
2.04
1.97
1.93
1.69
1.53
1.44
1.40
1.38
1.35
1.28
1.22
0.79
CH3
OH
H
H
H
O
NH
1.00
1.01
1.02
1.99
1.07
1.06
1.00
0.99
0.91
1.12
1.13
1.01
1.04
1.05
1.09
1.02
1.00
2.97
7.5
7.0
6.5
6.0
5.5
5.0
4.5
4.0
3.5
3.0
2.5
2.0
1.5
1.0
f1 (ppm)

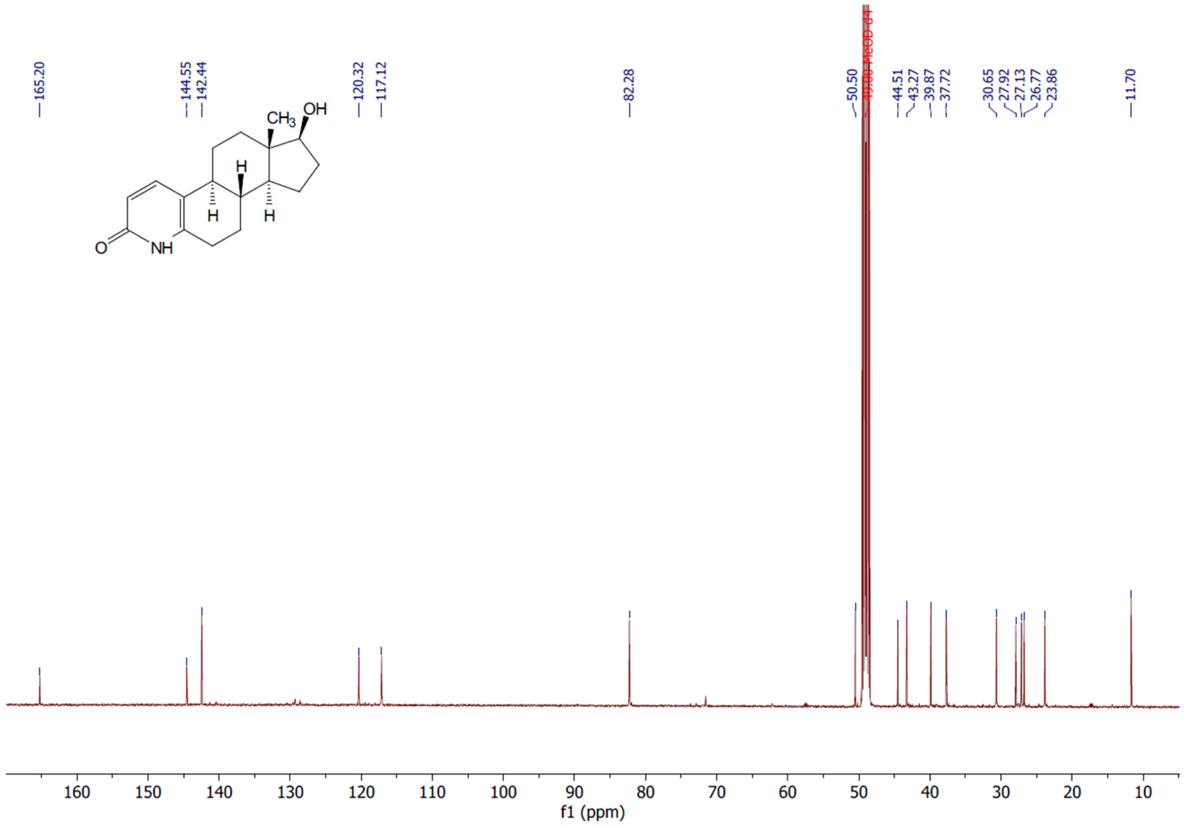

165.20
144.55
142.44
120.32
117.12
82.28
50.50
49.00 MeOD-d4
44.51
43.27
39.87
37.72
30.65
27.92
27.13
26.77
23.86
11.70
CH3
OH
H
H
H
O
NH
160
150
140
130
120
110
100
90
80
70
60
50
40
30
20
10
f1 (ppm)

Compound 7

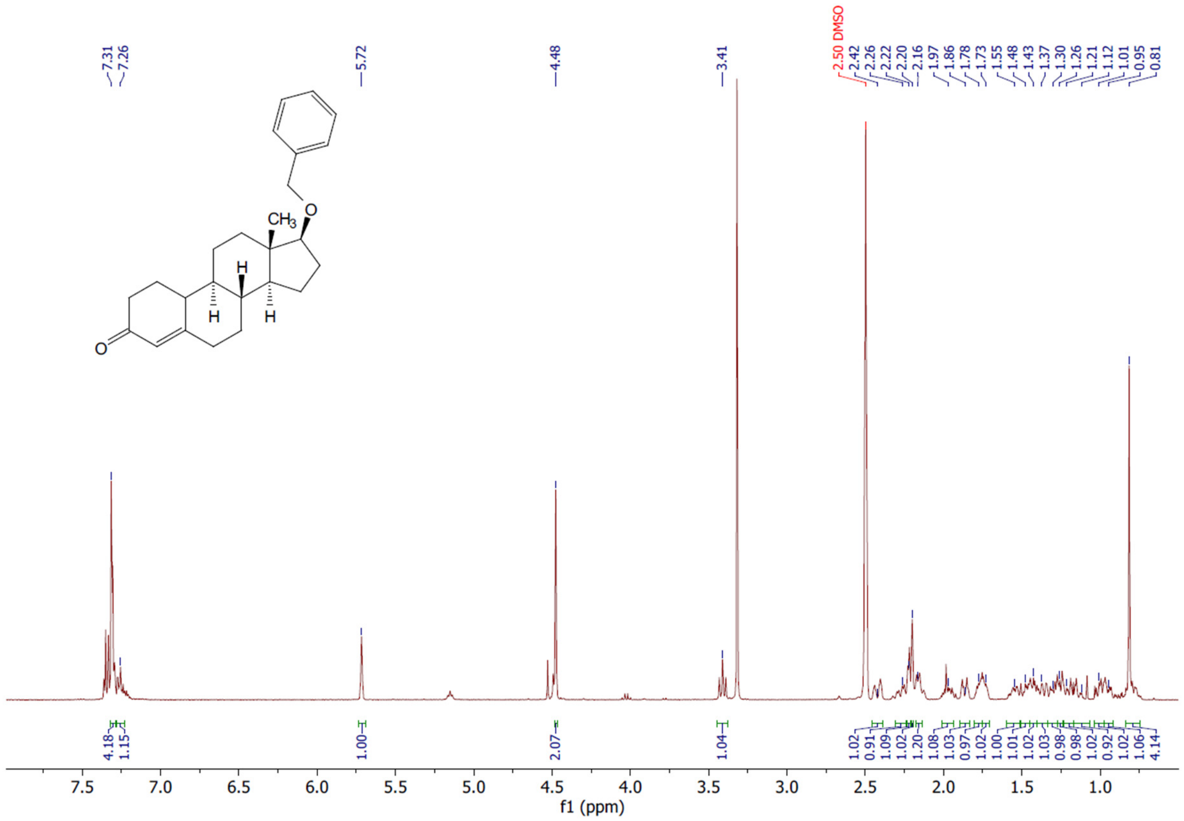

7.31
7.26
5.72
4.48
3.41
2.50 DMSO
2.42
2.26
2.22
2.20
2.16
1.97
1.86
1.78
1.73
1.55
1.48
1.43
1.37
1.30
1.26
1.21
1.12
1.01
0.95
0.81
CH3
O
H
H
H
O
4.18
1.15
1.00
2.07
1.04
1.02
0.91
1.09
1.02
1.20
1.08
1.03
0.97
1.02
1.00
1.01
1.02
1.03
0.98
0.98
1.02
0.92
1.02
1.06
4.14
7.5
7.0
6.5
6.0
5.5
5.0
4.5
4.0
3.5
3.0
2.5
2.0
1.5
1.0
f1 (ppm)

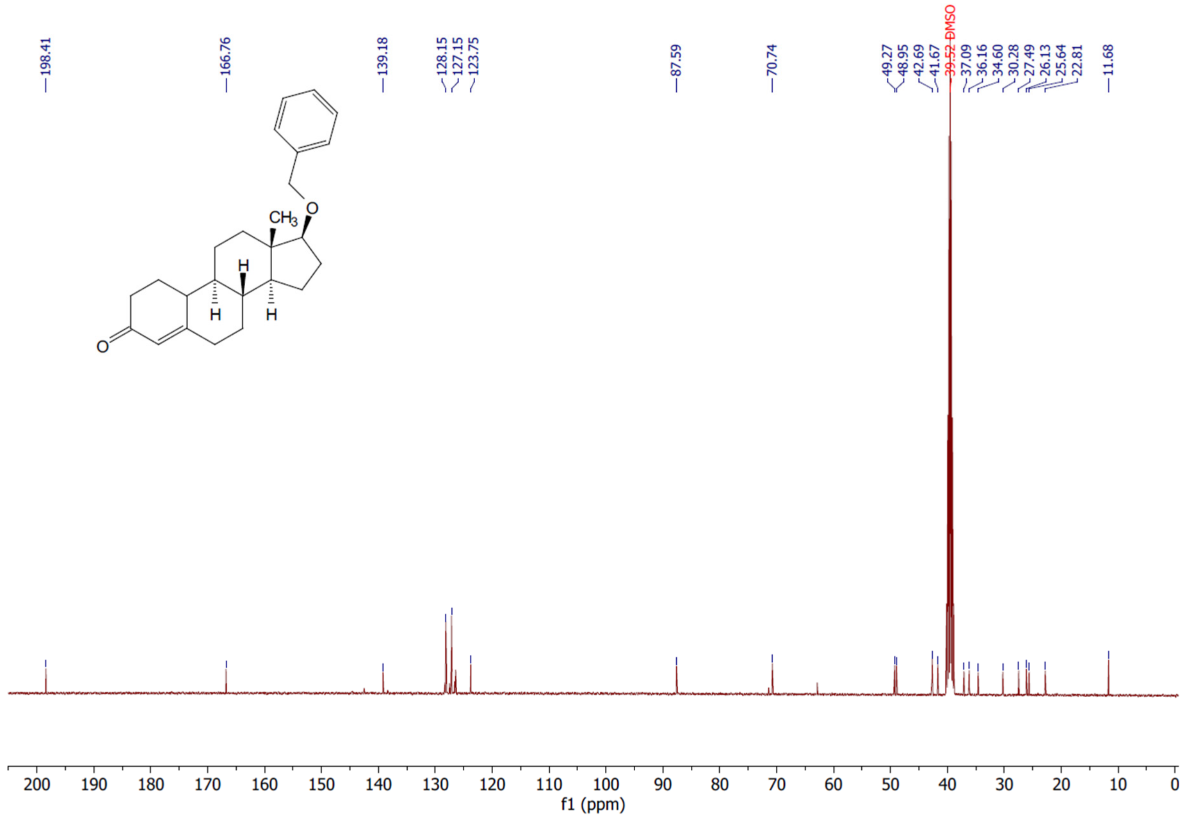

198.41
166.76
139.18
128.15
127.15
123.75
87.59
70.74
49.27
48.95
42.69
41.67
39.52 DMSO
37.09
36.16
34.60
30.28
27.49
26.13
25.64
22.81
11.68
CH3
O
H
H
H
O
200
190
180
170
160
150
140
130
120
110
100
90
80
70
60
50
40
30
20
10
0
f1 (ppm)

Compound 8

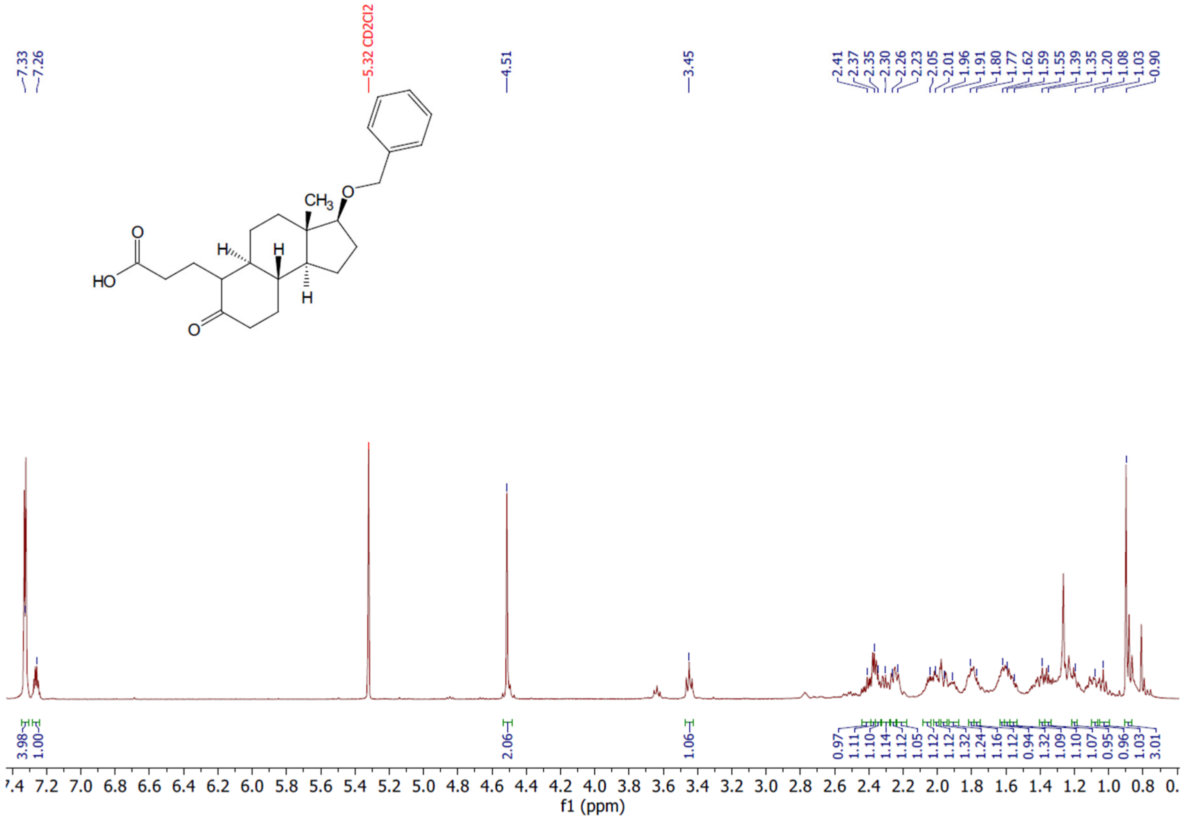

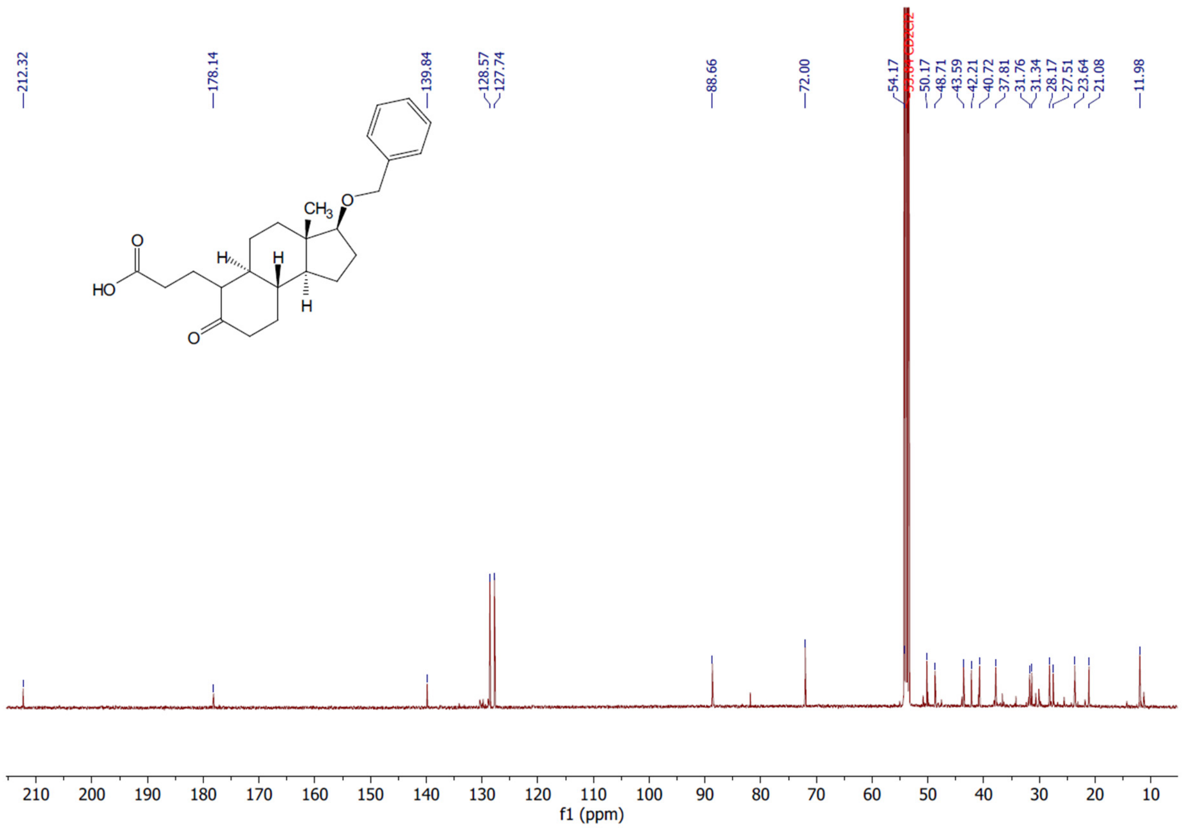

Compound 9a

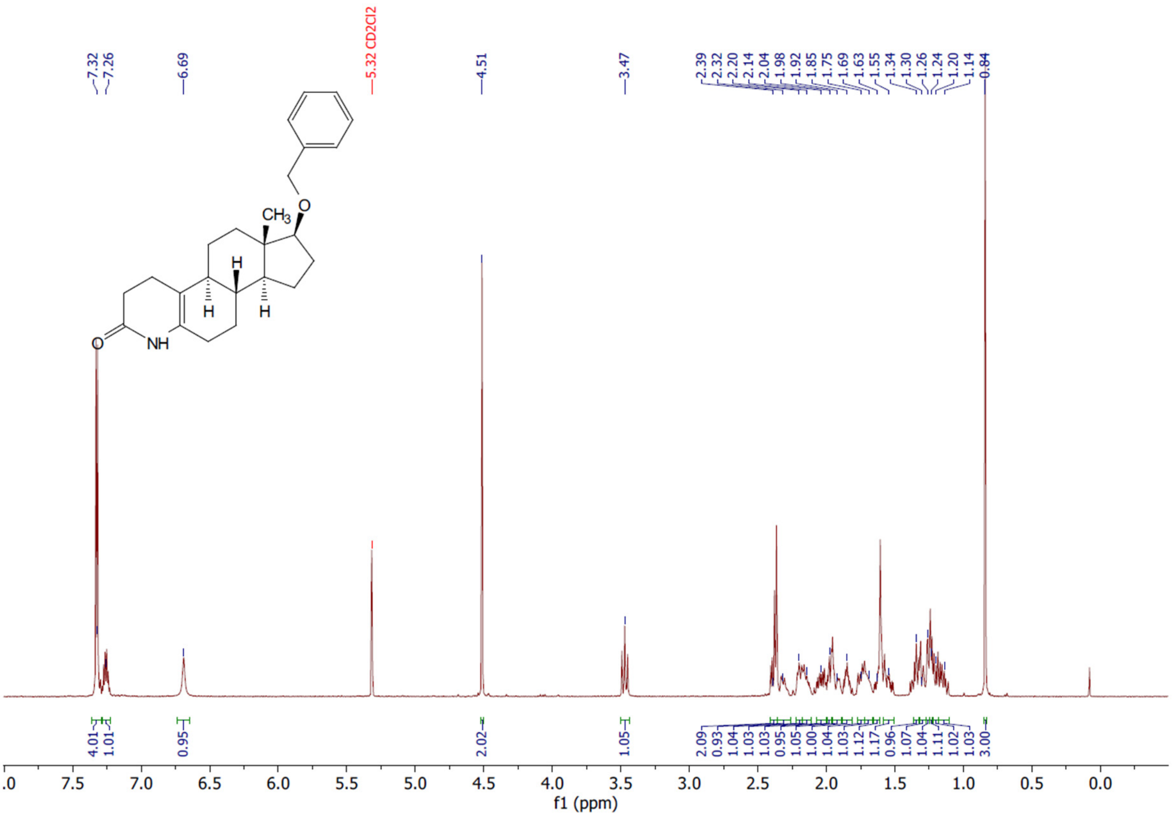
7.32
7.26
6.69
5.32 CD2Cl2
4.51
3.47
2.39
2.32
2.20
2.14
2.04
1.98
1.92
1.85
1.75
1.69
1.63
1.55
1.34
1.30
1.26
1.24
1.20
1.14
0.84
CH3
O
H
H
H
NH
O
4.01
1.01
0.95
2.02
1.05
2.09
0.93
1.04
1.03
1.03
0.95
1.05
1.00
1.04
1.03
1.12
1.17
0.96
1.07
1.04
1.11
1.02
1.03
3.00
f1 (ppm)

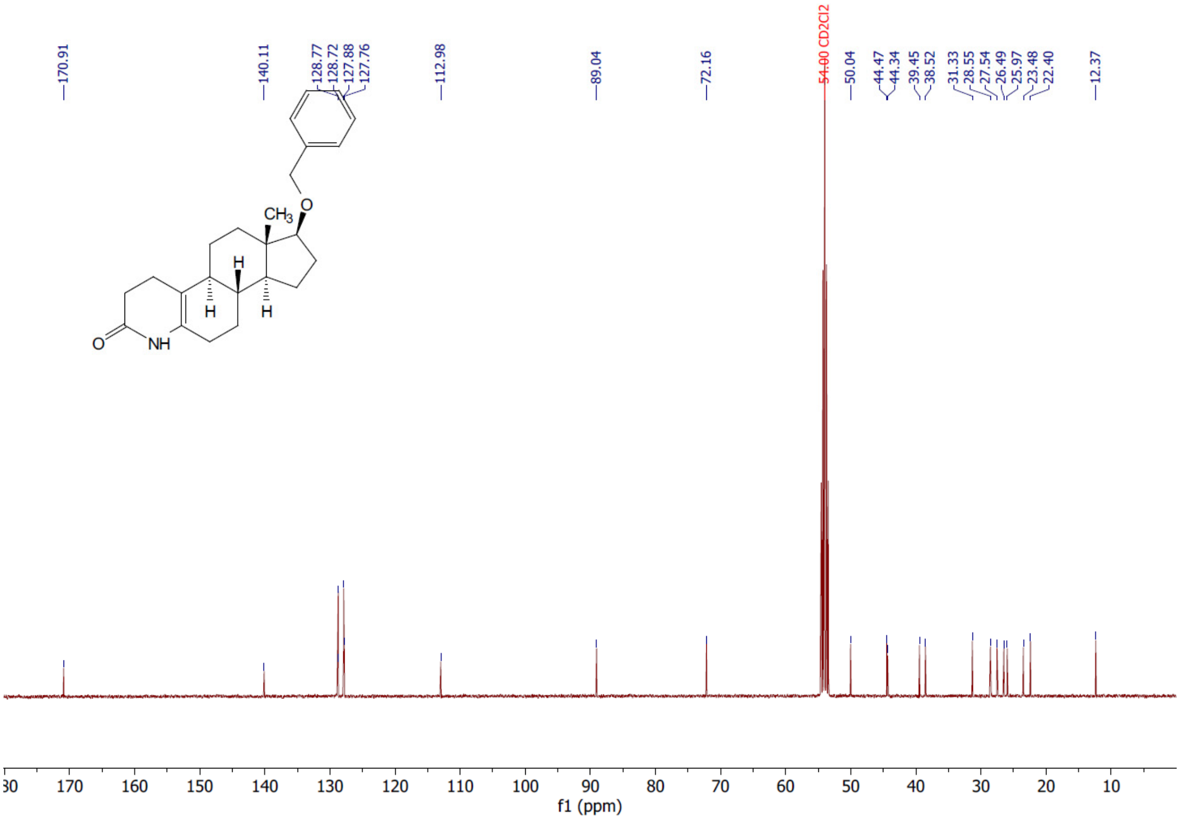
170.91
140.11
128.77
128.72
127.88
127.76
112.98
89.04
72.16
54.00 CD2Cl2
50.04
44.47
44.34
39.45
38.52
31.33
28.55
27.54
26.49
25.97
23.48
22.40
12.37
CH3
O
H
H
H
NH
O
f1 (ppm)

Compound 9b

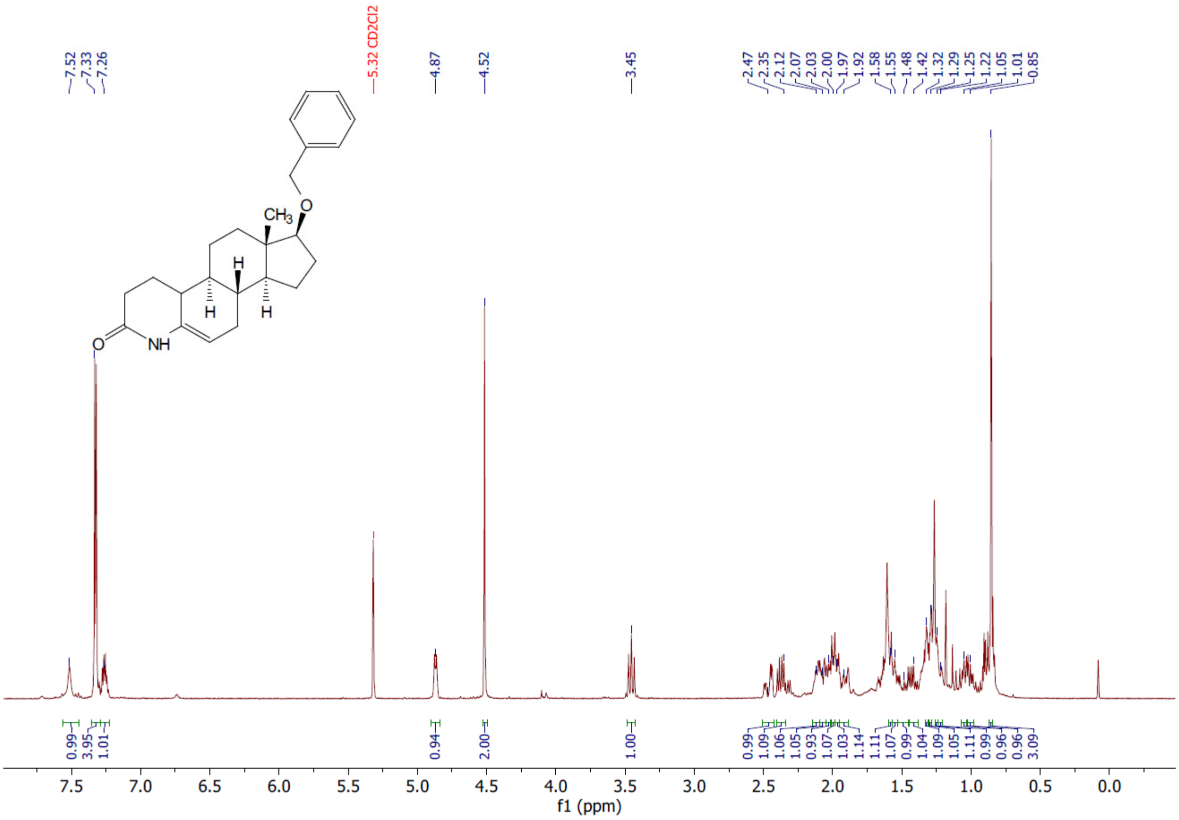

7.52
7.33
7.26
5.32 CD2Cl2
4.87
4.52
3.45
2.47
2.35
2.12
2.07
2.03
2.00
1.97
1.92
1.58
1.55
1.48
1.42
1.32
1.29
1.25
1.22
1.05
1.01
0.85
CH3
O
H
H
H
NH
O
0.99
3.95
1.01
0.94
2.00
1.00
0.99
1.09
1.06
1.05
0.93
1.07
1.03
1.14
1.11
1.07
0.99
1.04
1.09
1.05
1.11
0.99
0.96
0.96
3.09
7.5
7.0
6.5
6.0
5.5
5.0
4.5
4.0
3.5
3.0
2.5
2.0
1.5
1.0
0.5
0.0
f1 (ppm)

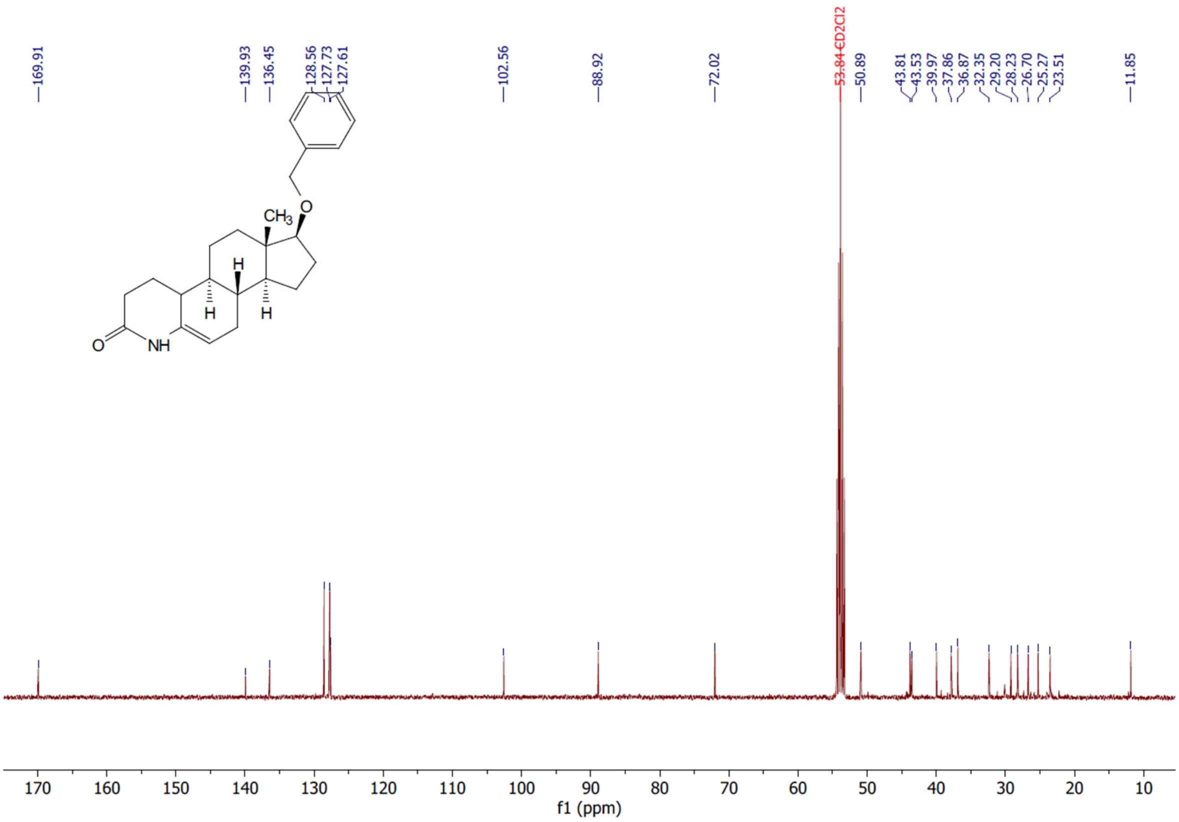

169.91
139.93
136.45
128.56
127.73
127.61
102.56
88.92
72.02
53.84 CD2Cl2
50.89
43.81
43.53
39.97
37.86
36.87
32.35
29.20
28.23
26.70
25.27
23.51
11.85
CH3
O
H
H
H
NH
O
170
160
150
140
130
120
110
100
90
80
70
60
50
40
30
20
10
f1 (ppm)

Compound 10

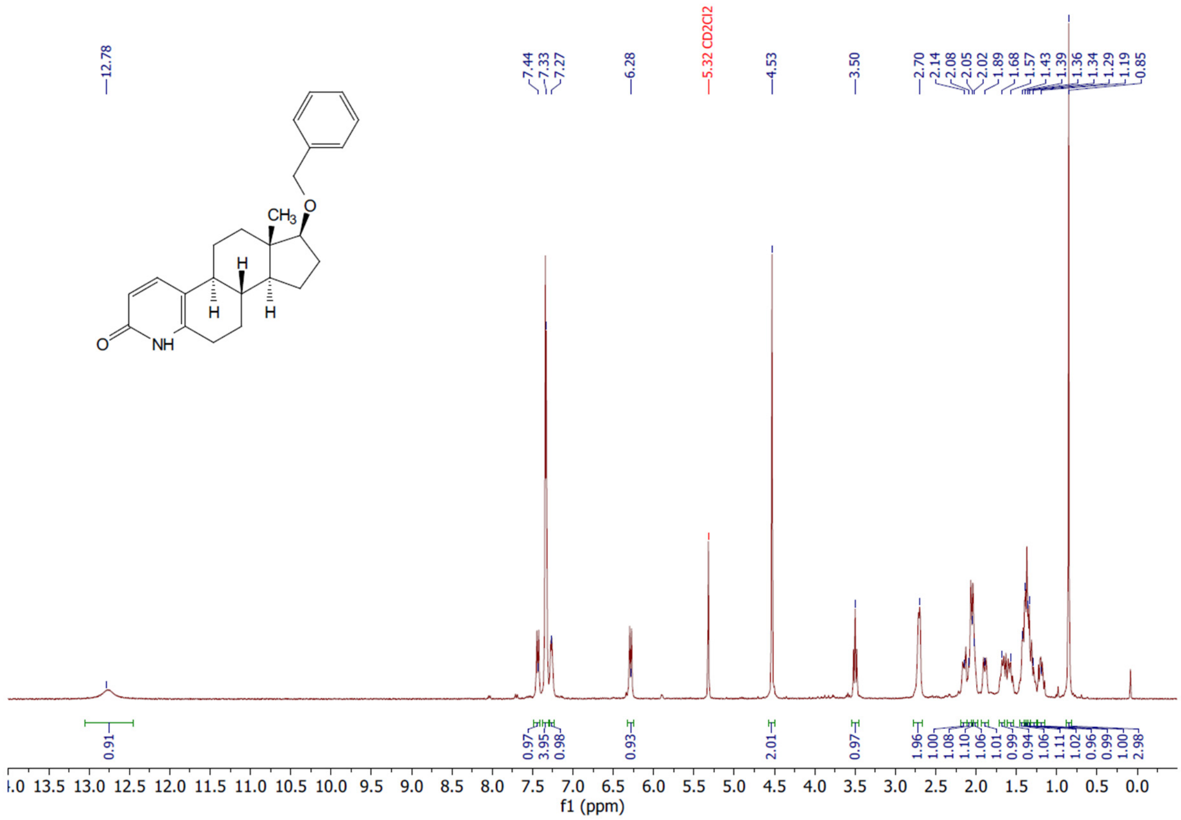

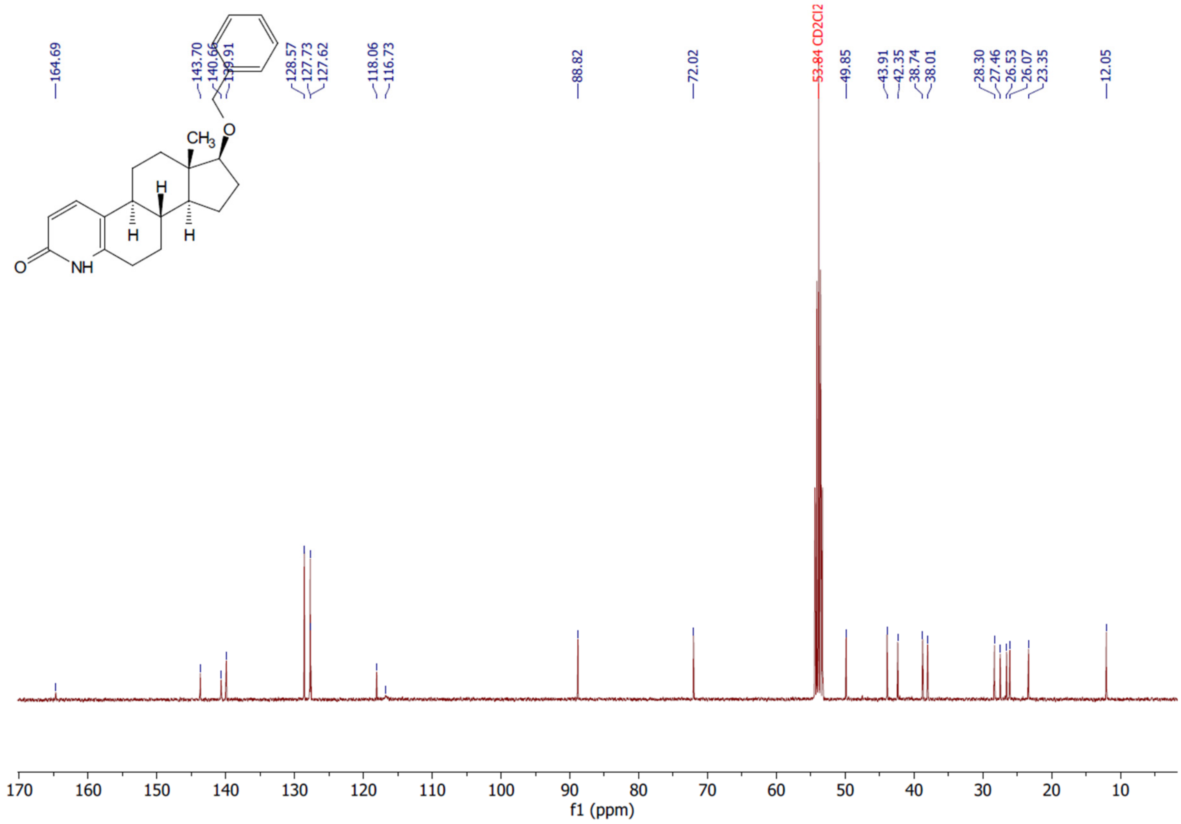

Compound 11

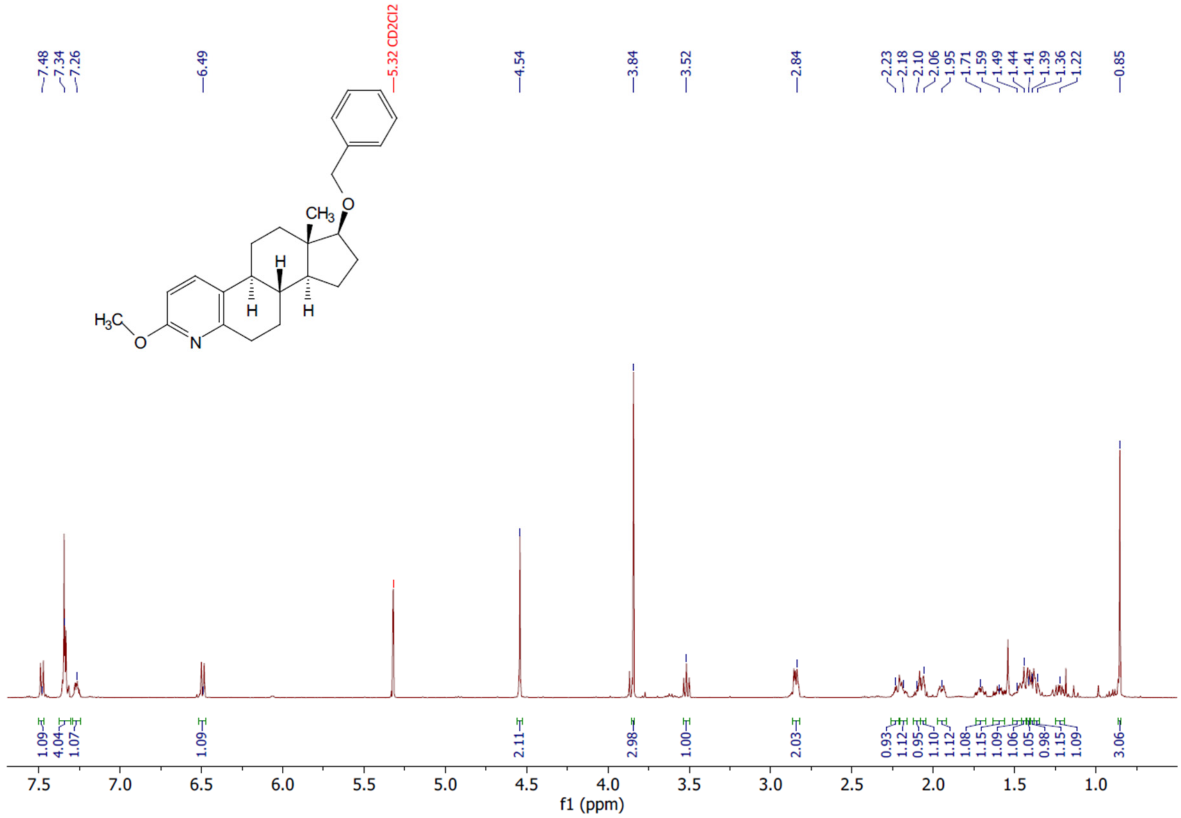

7.48
7.34
7.26
6.49
5.32 CD2Cl2
4.54
3.84
3.52
2.84
2.23
2.18
2.10
2.06
1.95
1.71
1.59
1.49
1.44
1.41
1.39
1.36
1.22
0.85
CH3
H
H
H
H3C
O
N
O
1.09
4.04
1.07
1.09
2.11
2.98
1.00
2.03
0.93
1.12
0.95
1.10
1.12
1.08
1.15
1.09
1.06
1.05
0.98
1.15
1.09
3.06
7.5
7.0
6.5
6.0
5.5
5.0
4.5
4.0
3.5
3.0
2.5
2.0
1.5
1.0
f1 (ppm)

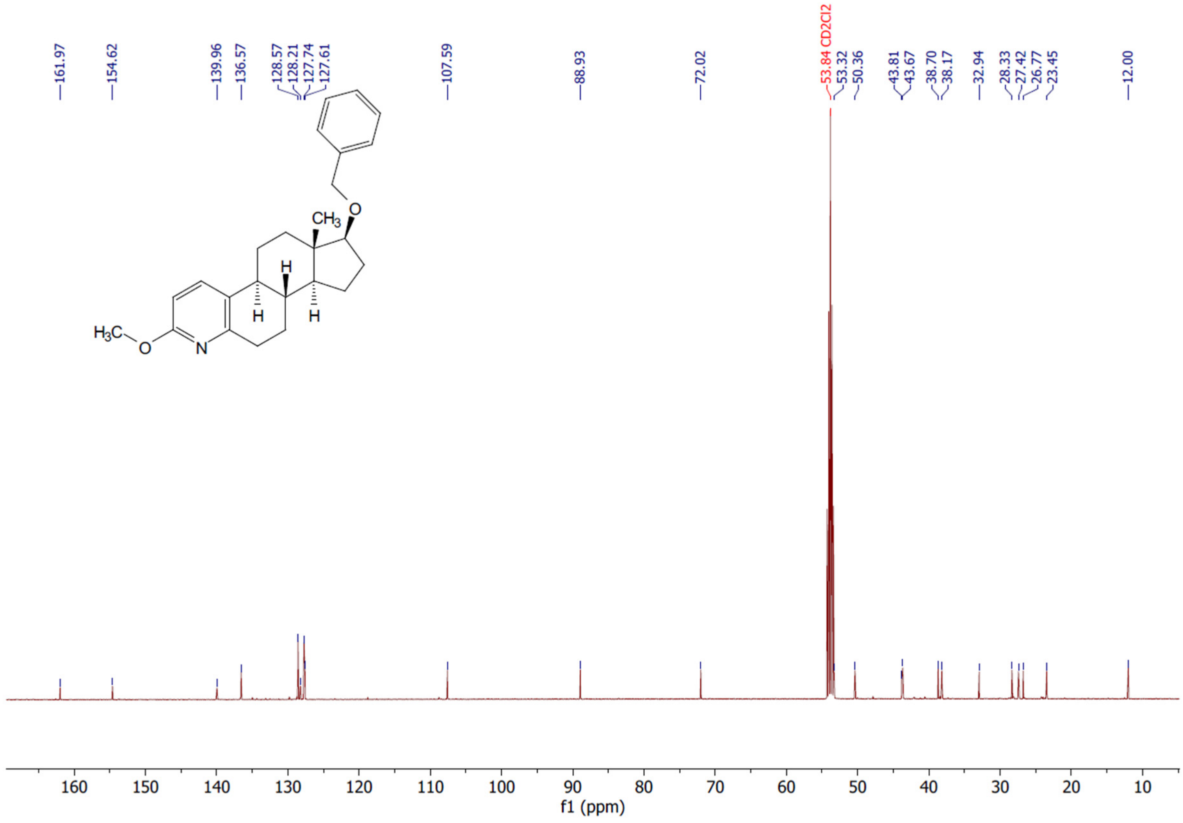

161.97
154.62
139.96
136.57
128.57
128.21
127.74
127.61
107.59
88.93
72.02
53.84 CD2Cl2
53.32
50.36
43.81
43.67
38.70
38.17
32.94
28.33
27.42
26.77
23.45
12.00
CH3
H
H
H
H3C
O
N
O
160
150
140
130
120
110
100
90
80
70
60
50
40
30
20
10
f1 (ppm)

Compound 1

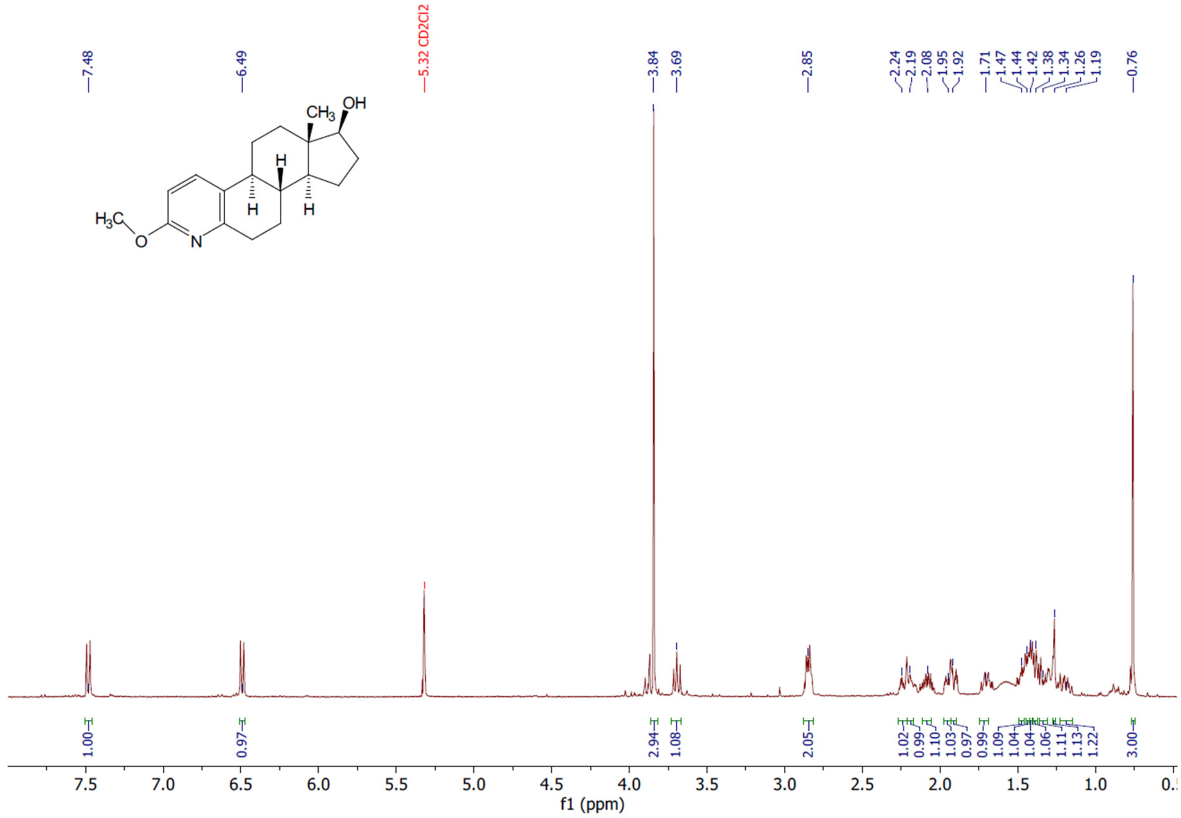

7.48
6.49
5.32 CD2Cl2
3.84
3.69
2.85
2.24
2.19
2.08
1.95
1.92
1.71
1.47
1.44
1.42
1.38
1.34
1.26
1.19
0.76
1.00
0.97
2.94
1.08
2.05
1.02
0.99
1.10
1.03
0.97
0.99
1.09
1.04
1.04
1.06
1.11
1.13
1.22
3.00
f1 (ppm)

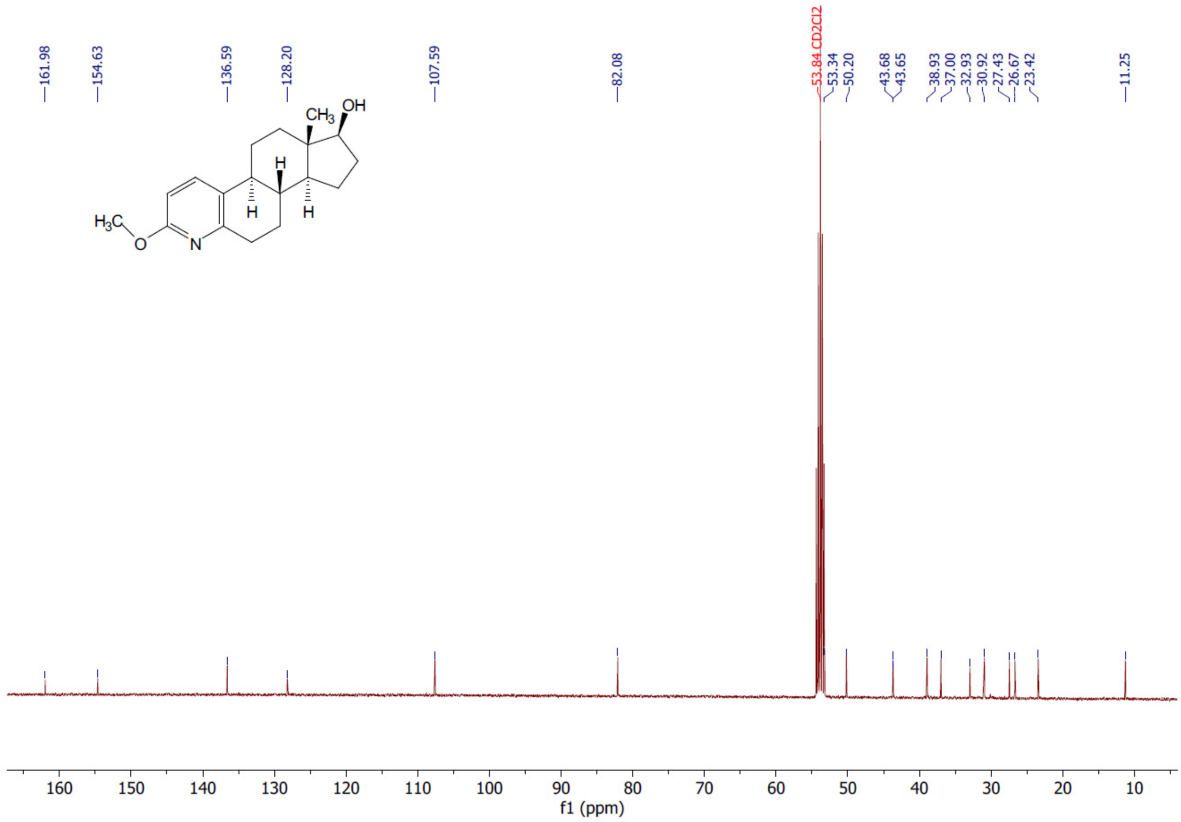

161.98
154.63
136.59
128.20
107.59
82.08
53.84 CD2Cl2
53.34
50.20
43.68
43.65
38.93
37.00
32.93
30.92
27.43
26.67
23.42
11.25
f1 (ppm)

Compound 12

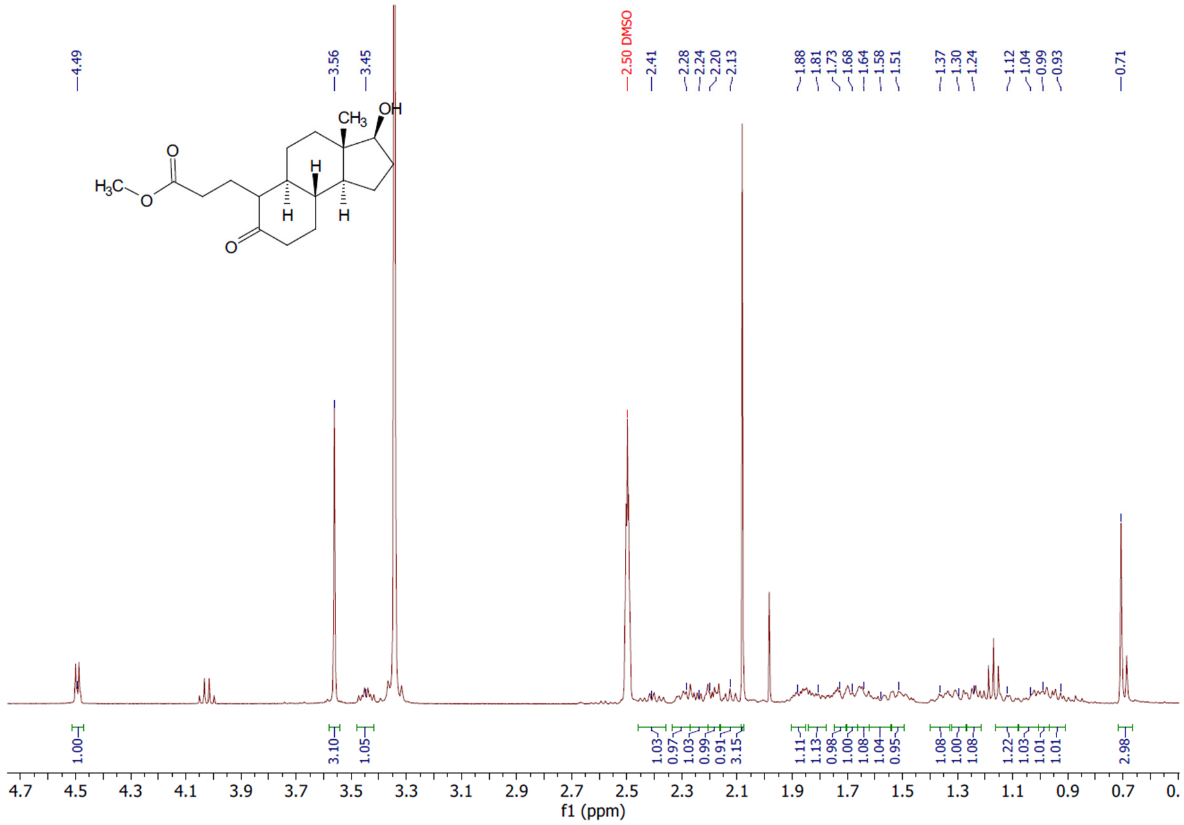

4.49
3.56
3.45
2.50 DMSO
2.41
2.28
2.24
2.20
2.13
1.88
1.81
1.73
1.68
1.64
1.58
1.51
1.37
1.30
1.24
1.12
1.04
0.99
0.93
0.71
CH3
OH
O
H3C
O
H
H
H
O
1.00
3.10
1.05
1.03
0.97
1.03
0.99
0.91
3.15
1.11
1.13
0.98
1.00
1.08
1.04
0.95
1.08
1.00
1.08
1.22
1.03
1.01
1.01
2.98
4.7
4.5
4.3
4.1
3.9
3.7
3.5
3.3
3.1
2.9
2.7
2.5
2.3
2.1
1.9
1.7
1.5
1.3
1.1
0.9
0.7
f1 (ppm)

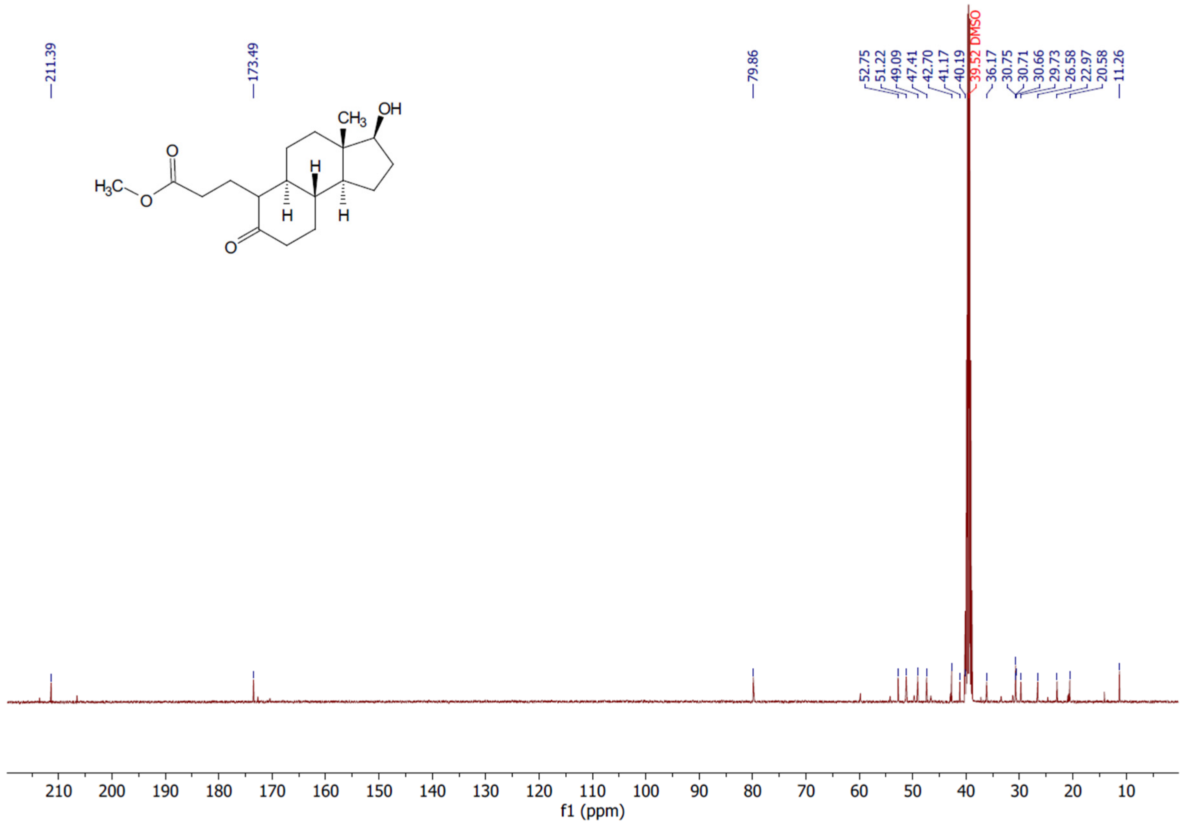

211.39
173.49
79.86
52.75
51.22
49.09
47.41
42.70
41.17
40.19
39.52 DMSO
36.17
30.75
30.71
30.66
29.73
26.58
22.97
20.58
11.26
CH3
OH
O
H3C
O
H
H
H
O
210
200
190
180
170
160
150
140
130
120
110
100
90
80
70
60
50
40
30
20
10
f1 (ppm)

Compound 13

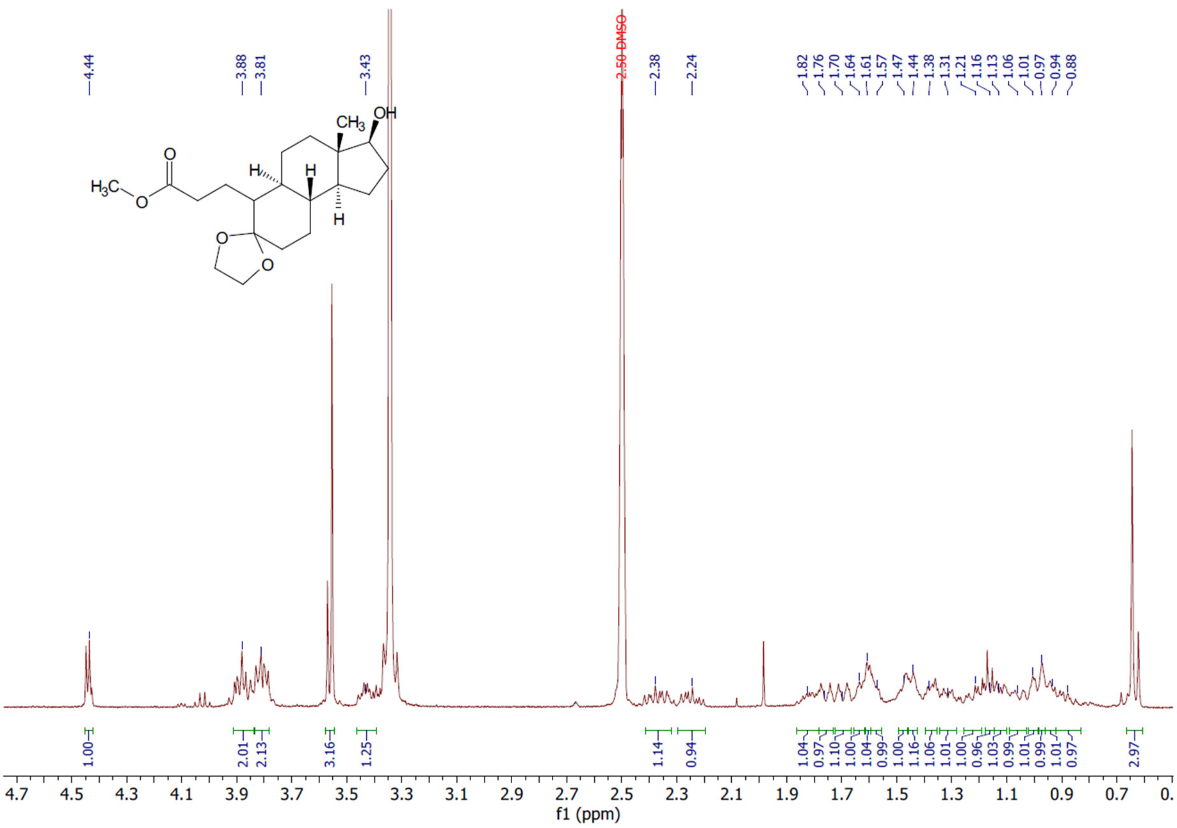
4.44
3.88
3.81
3.43
2.50 DMSO
2.38
2.24
1.82
1.76
1.70
1.64
1.61
1.57
1.47
1.44
1.38
1.31
1.21
1.16
1.13
1.06
1.01
0.97
0.94
0.88
CH3
OH
O
H3C
O
H
H
H
O
O
1.00
2.01
2.13
3.16
1.25
1.14
0.94
1.04
0.97
1.10
1.00
1.04
0.99
1.00
1.16
1.06
1.01
1.00
0.96
1.03
0.99
1.01
0.99
1.01
0.97
2.97
f1 (ppm)

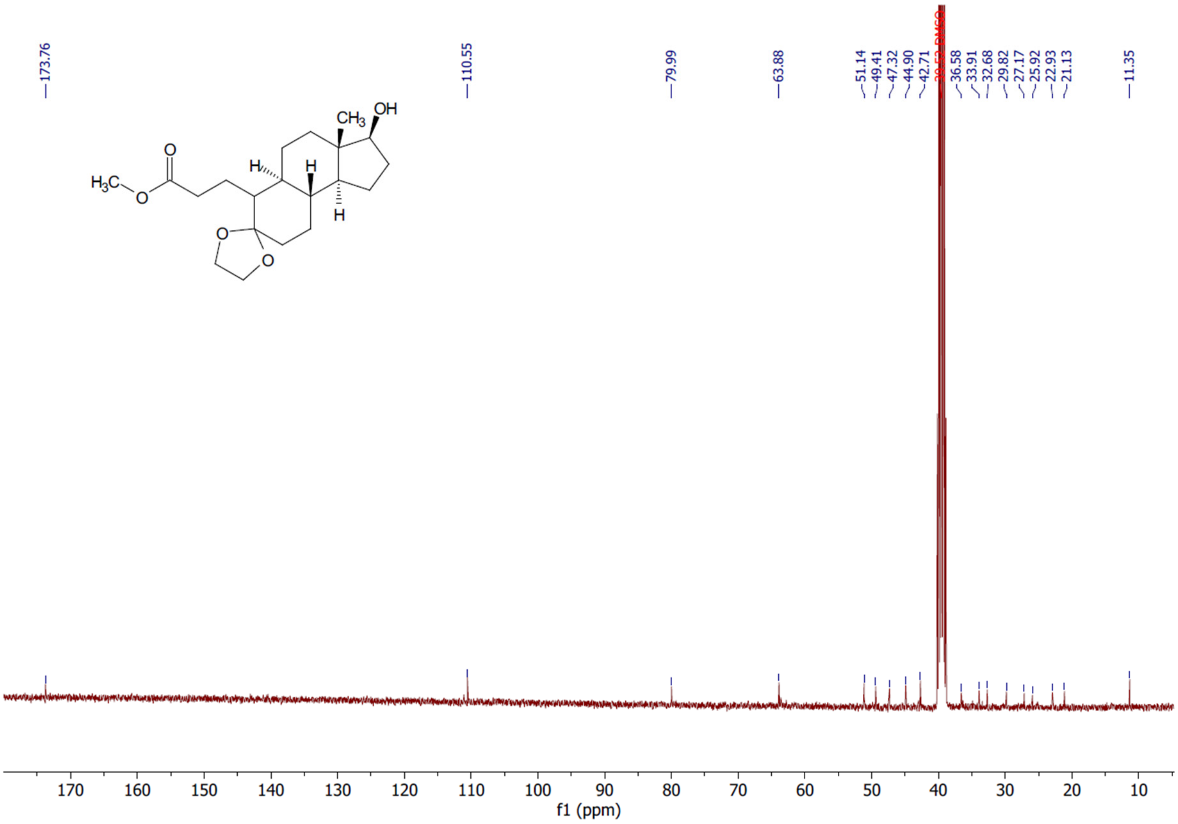
173.76
110.55
79.99
63.88
51.14
49.41
47.32
44.90
42.71
39.52 DMSO
36.58
33.91
32.68
29.82
27.17
25.92
22.93
21.13
11.35
CH3
OH
O
H3C
O
H
H
H
O
O
f1 (ppm)

Compound 14

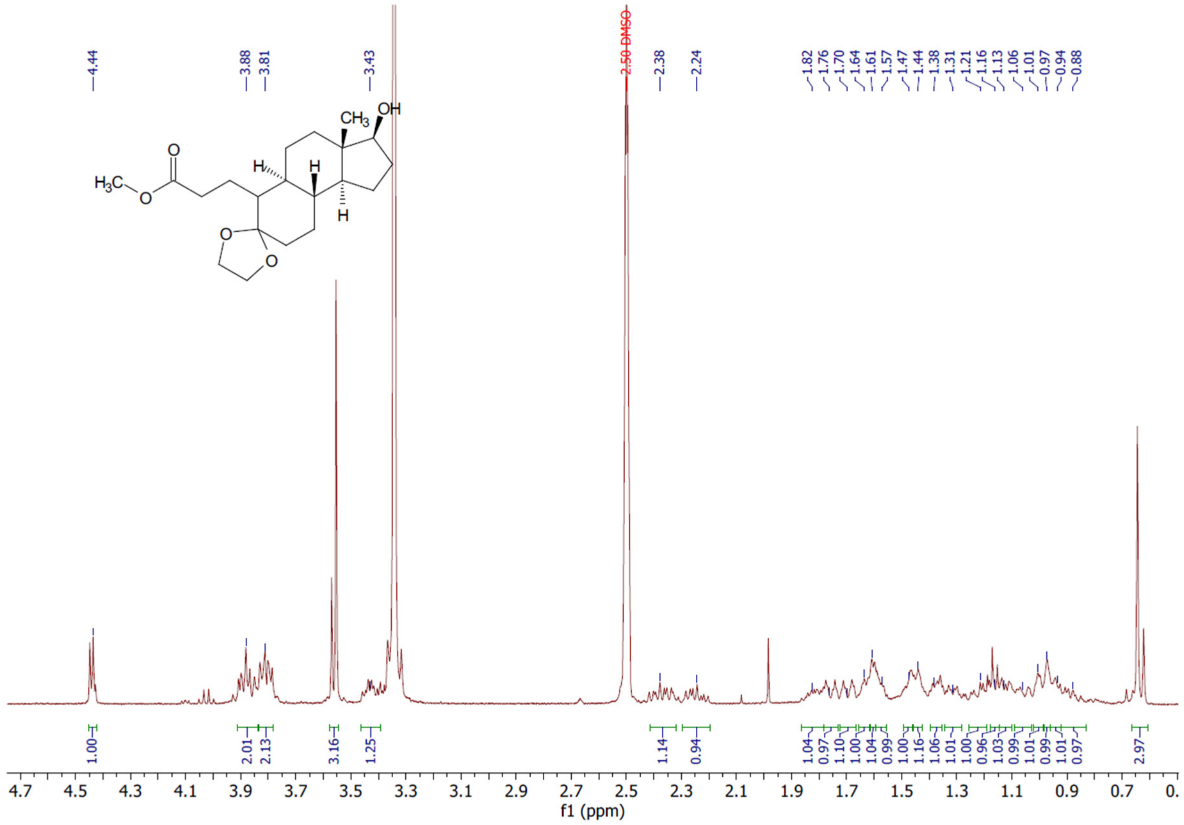

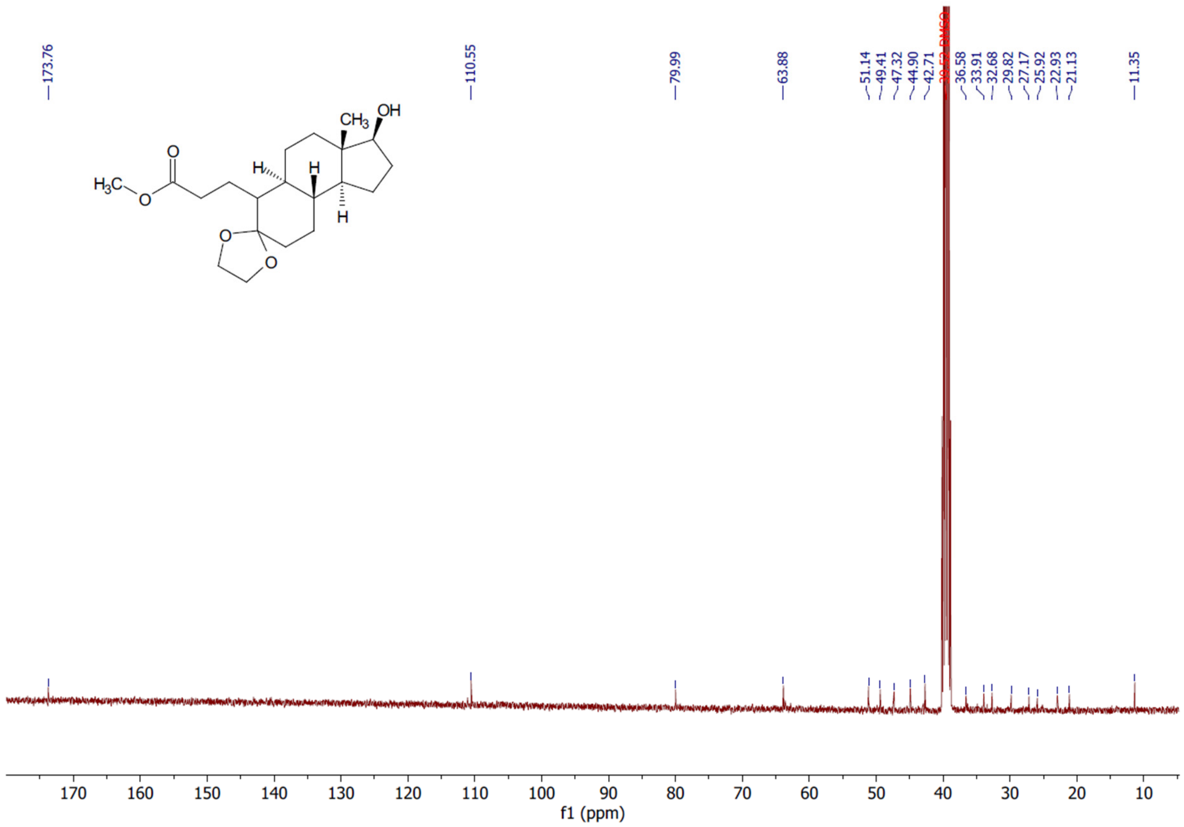

Compound 15

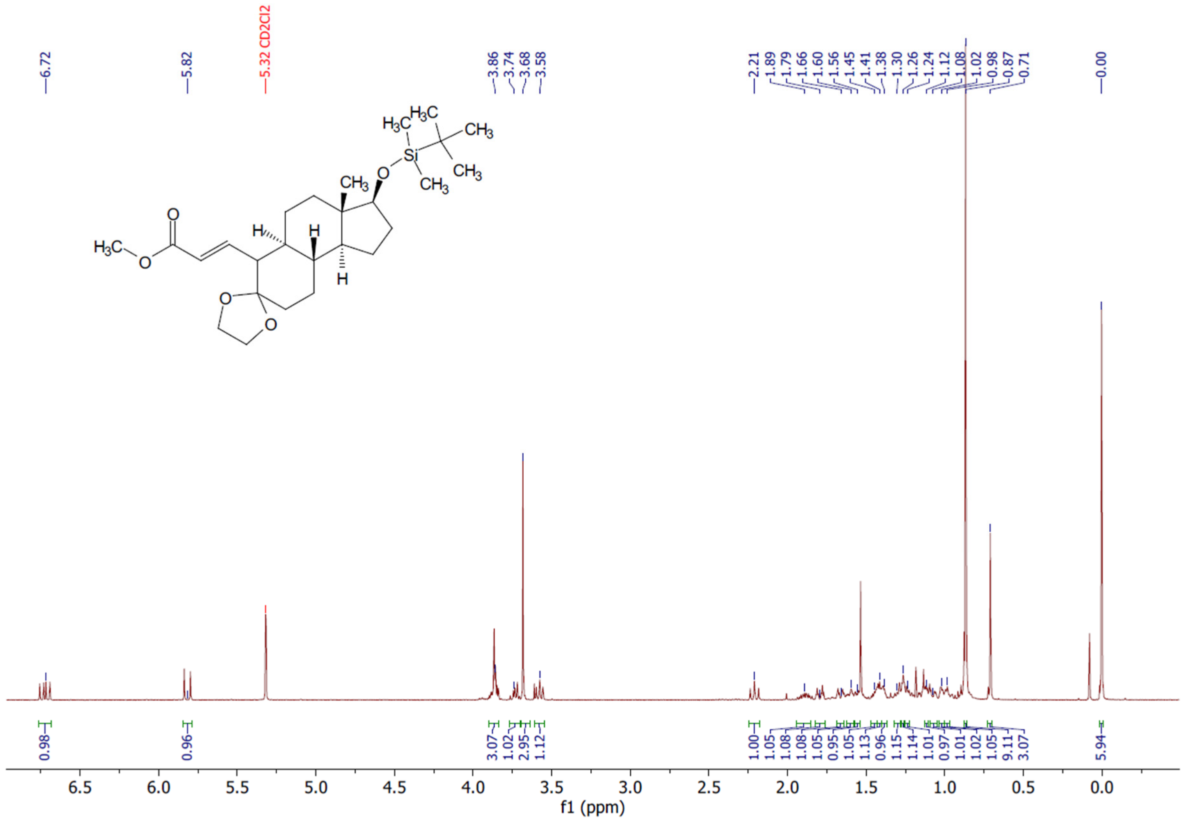

6.72
5.82
5.32 CD2Cl2
3.86
3.74
3.68
3.58
2.21
1.89
1.79
1.66
1.60
1.56
1.45
1.41
1.38
1.30
1.26
1.24
1.12
1.08
1.02
0.98
0.87
0.71
0.00
0.98
0.96
3.07
1.02
2.95
1.12
1.00
1.05
1.08
1.08
1.05
0.95
1.05
1.05
1.13
0.96
1.15
1.14
1.01
0.97
1.01
1.02
1.05
9.11
3.07
5.94
f1 (ppm)

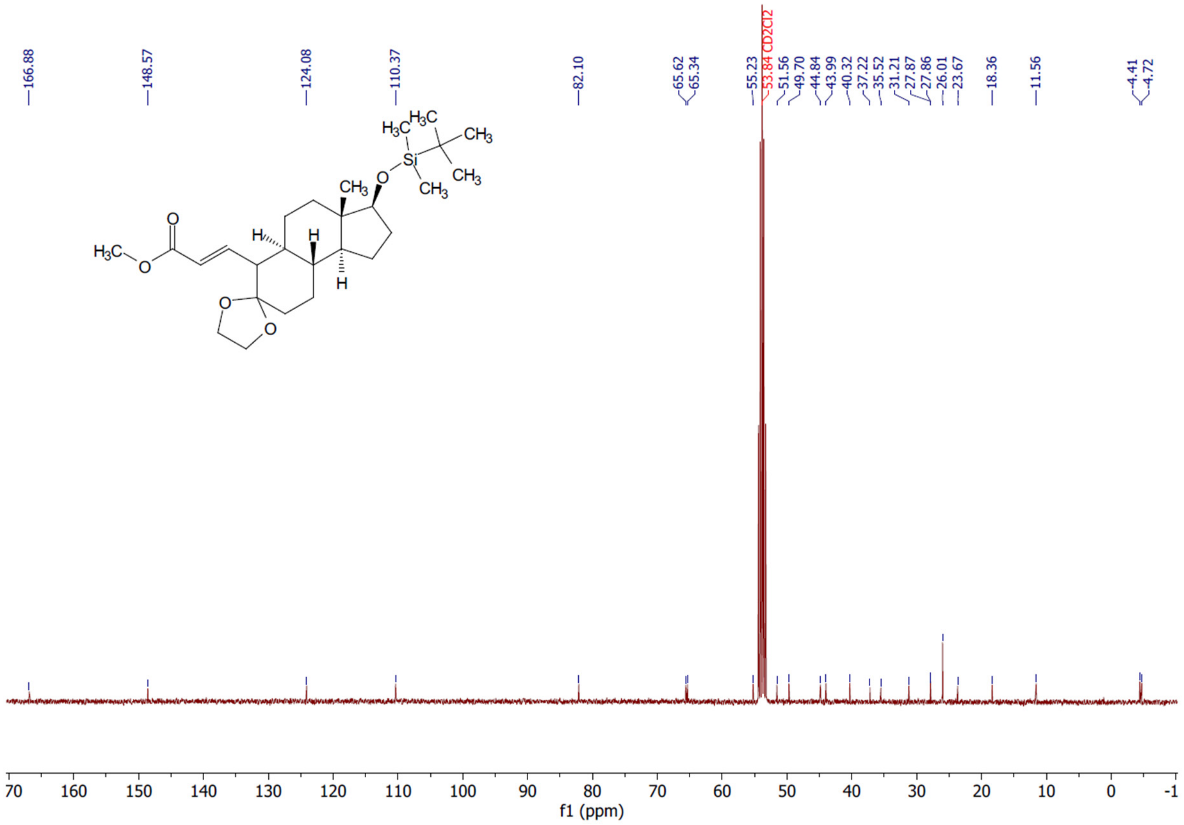

166.88
148.57
124.08
110.37
82.10
65.62
65.34
55.23
53.84 CD2Cl2
51.56
49.70
44.84
43.99
40.32
37.22
35.52
31.21
27.87
27.86
26.01
23.67
18.36
11.56
-4.41
-4.72
f1 (ppm)

Compound 16

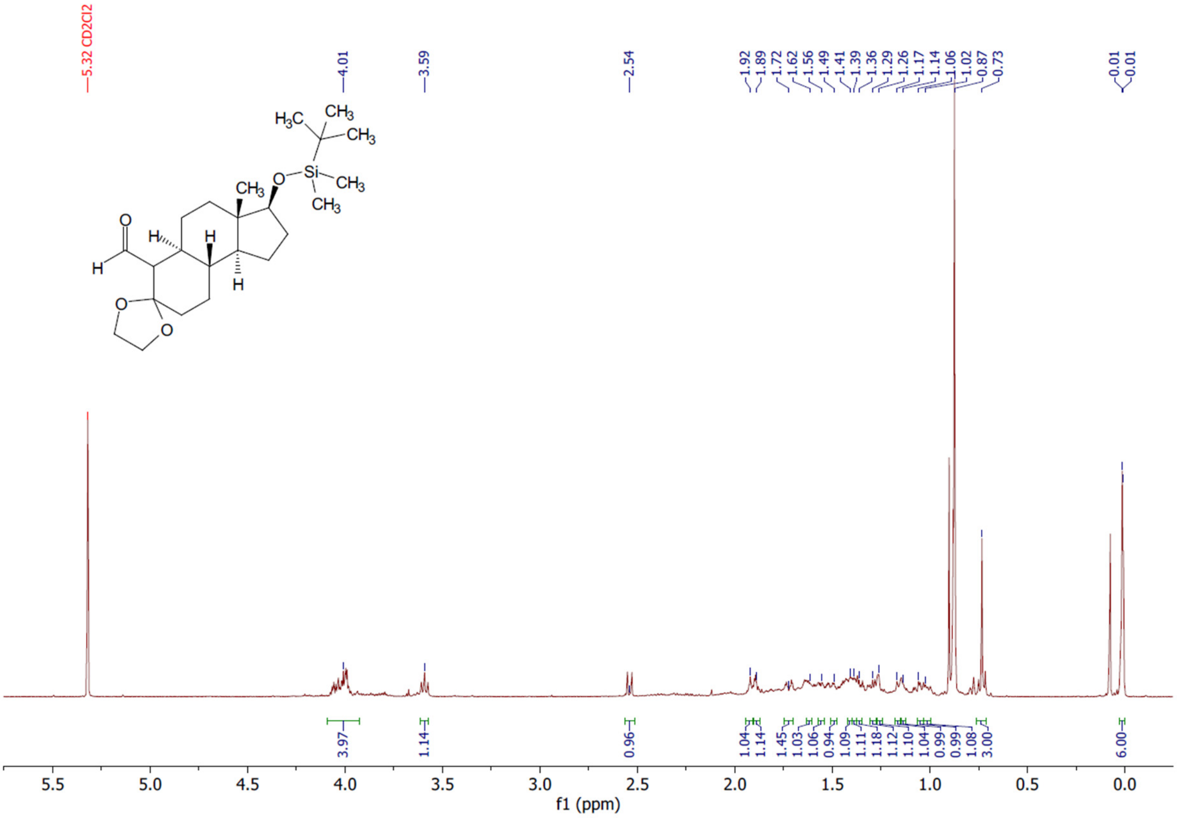
5.32 CD2Cl2
4.01
3.59
2.54
1.92
1.89
1.72
1.62
1.56
1.49
1.41
1.39
1.36
1.29
1.26
1.17
1.14
1.06
1.02
0.87
0.73
0.01
0.01
3.97
1.14
0.96
1.04
1.14
1.45
1.03
1.06
0.94
1.09
1.11
1.18
1.12
1.10
1.04
0.99
0.99
1.08
3.00
6.00
f1 (ppm)

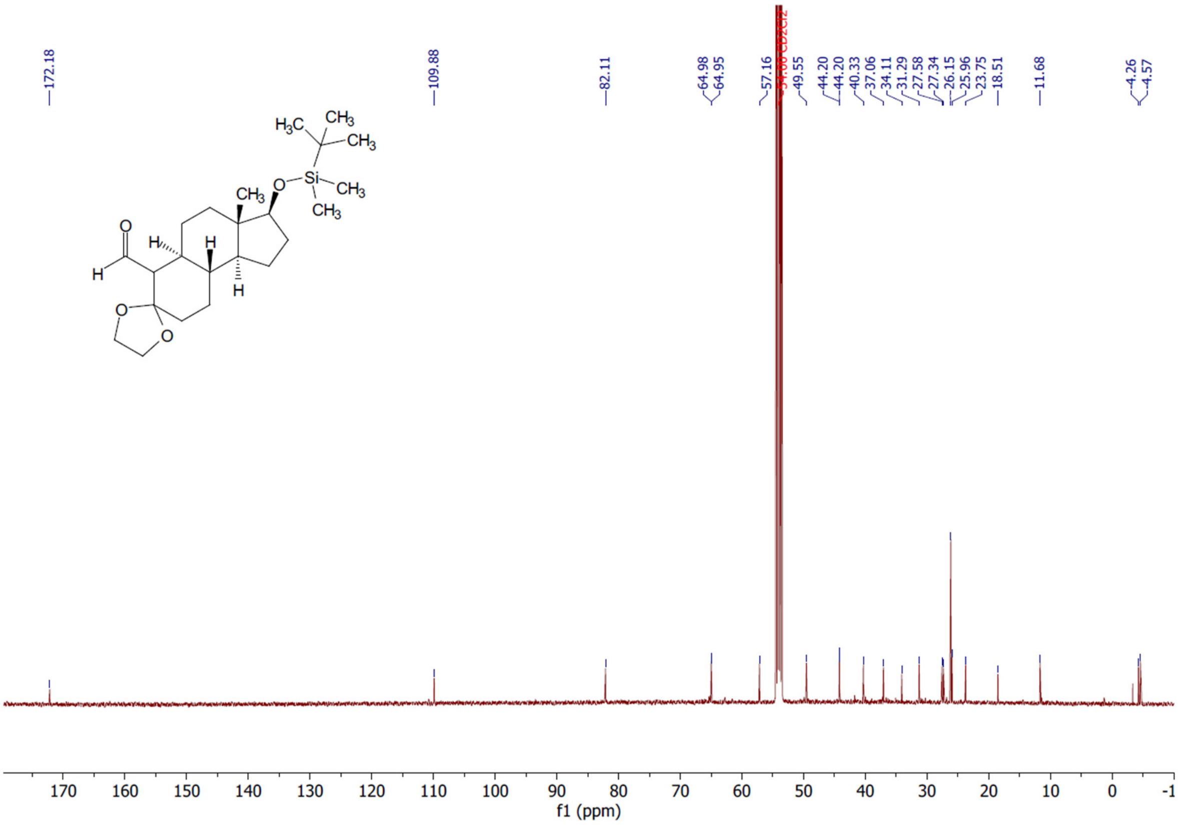
172.18
109.88
82.11
64.98
64.95
57.16
49.55
44.20
44.20
40.33
37.06
34.11
31.29
27.58
27.34
26.15
25.96
23.75
18.51
11.68
-4.26
-4.57
f1 (ppm)

Compound 17

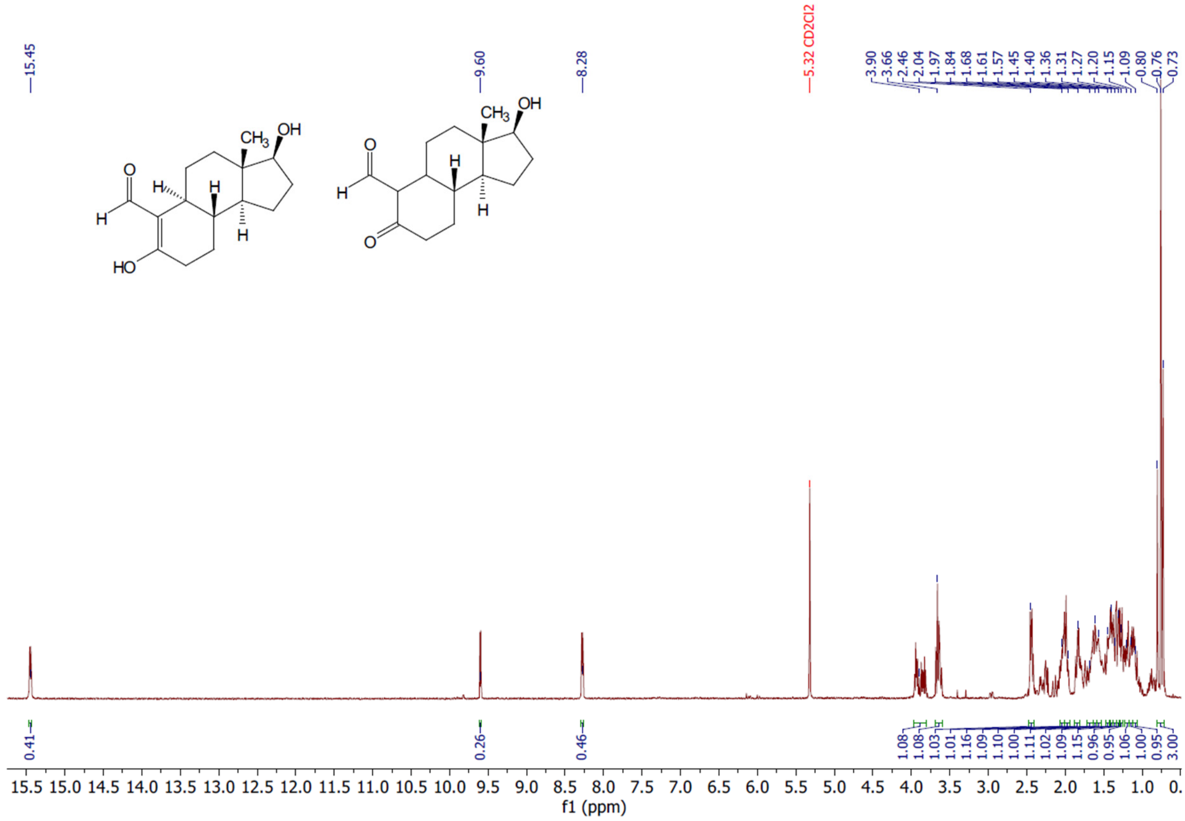

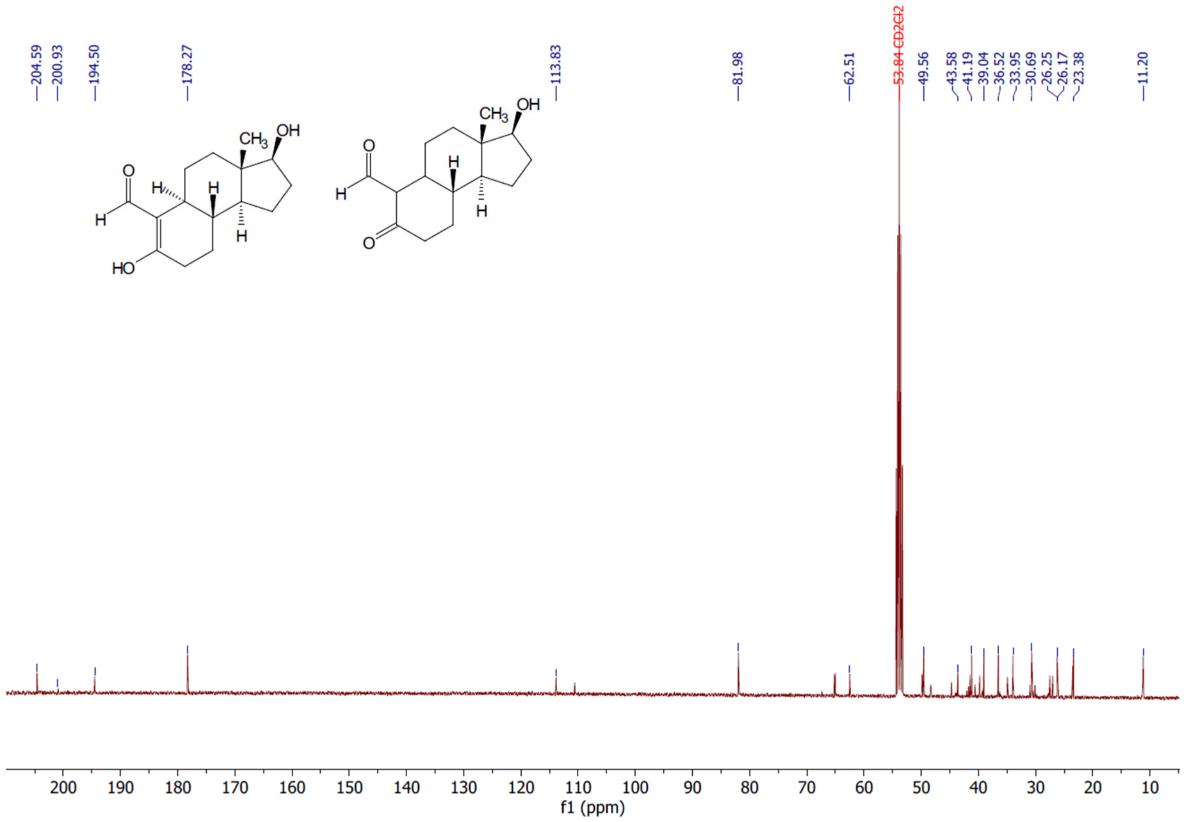

Compound 2

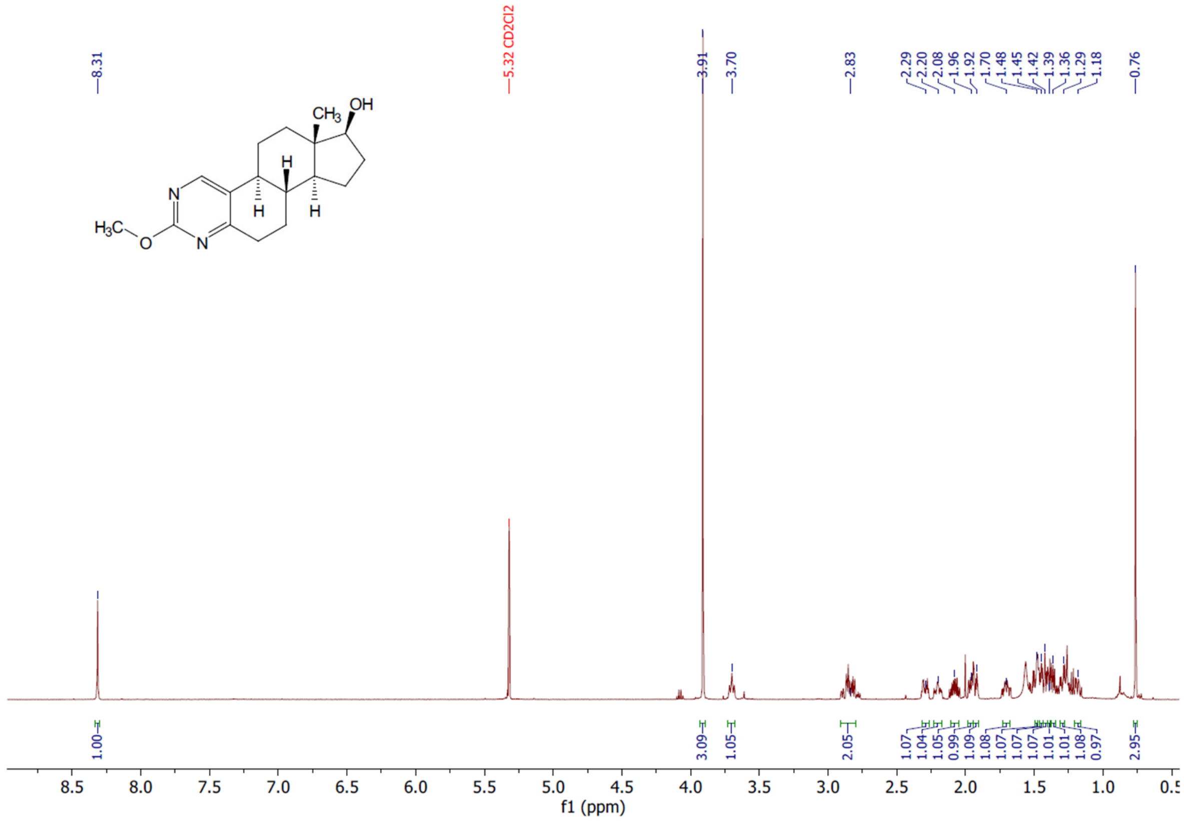
8.31
5.32 CD2Cl2
3.91
3.70
2.83
2.29
2.20
2.08
1.96
1.92
1.70
1.48
1.45
1.42
1.39
1.36
1.29
1.18
0.76
CH3
OH
H
H
H
N
N
H3C
O
1.00
3.09
1.05
2.05
1.07
1.04
1.05
0.99
1.09
1.08
1.07
1.07
1.07
1.01
1.08
0.97
2.95
f1 (ppm)

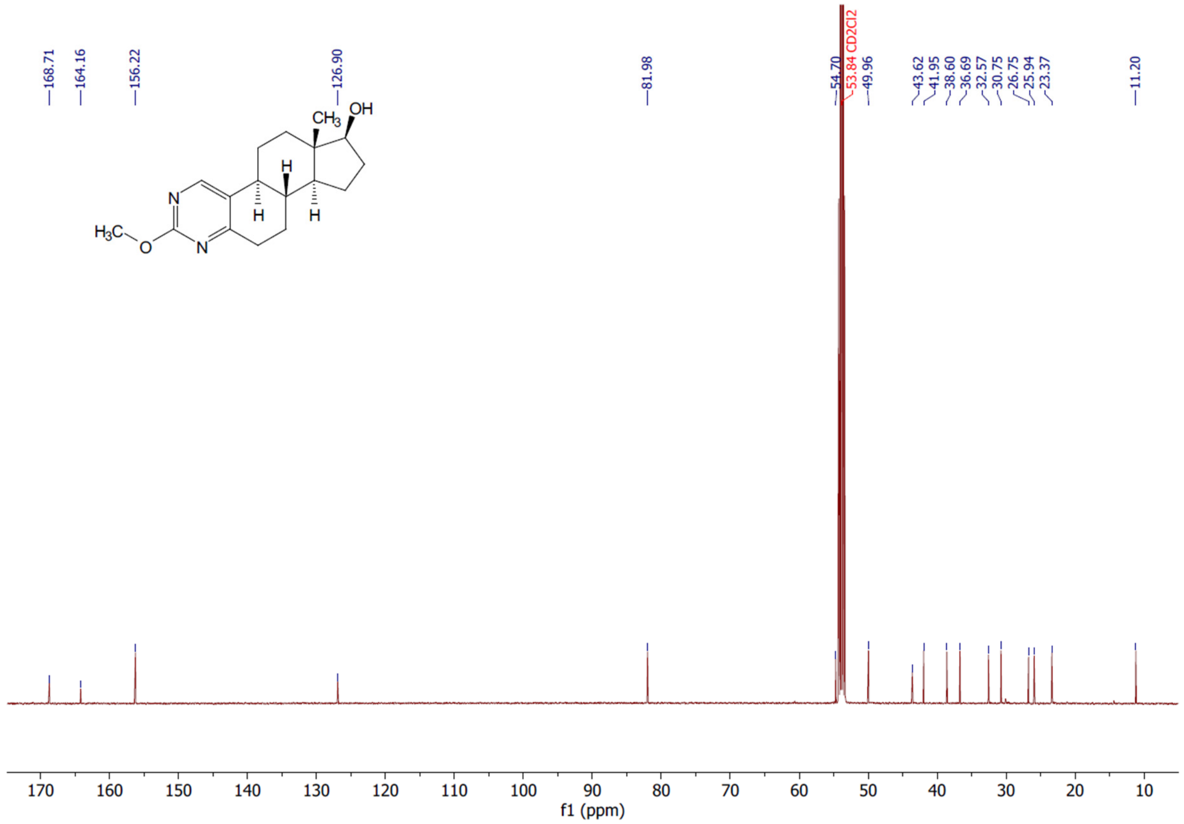
168.71
164.16
156.22
126.90
81.98
54.70
53.84 CD2Cl2
49.96
43.62
41.95
38.60
36.69
32.57
30.75
26.75
25.94
23.37
11.20
CH3
OH
H
H
H
N
N
H3C
O
f1 (ppm)

Compound S1

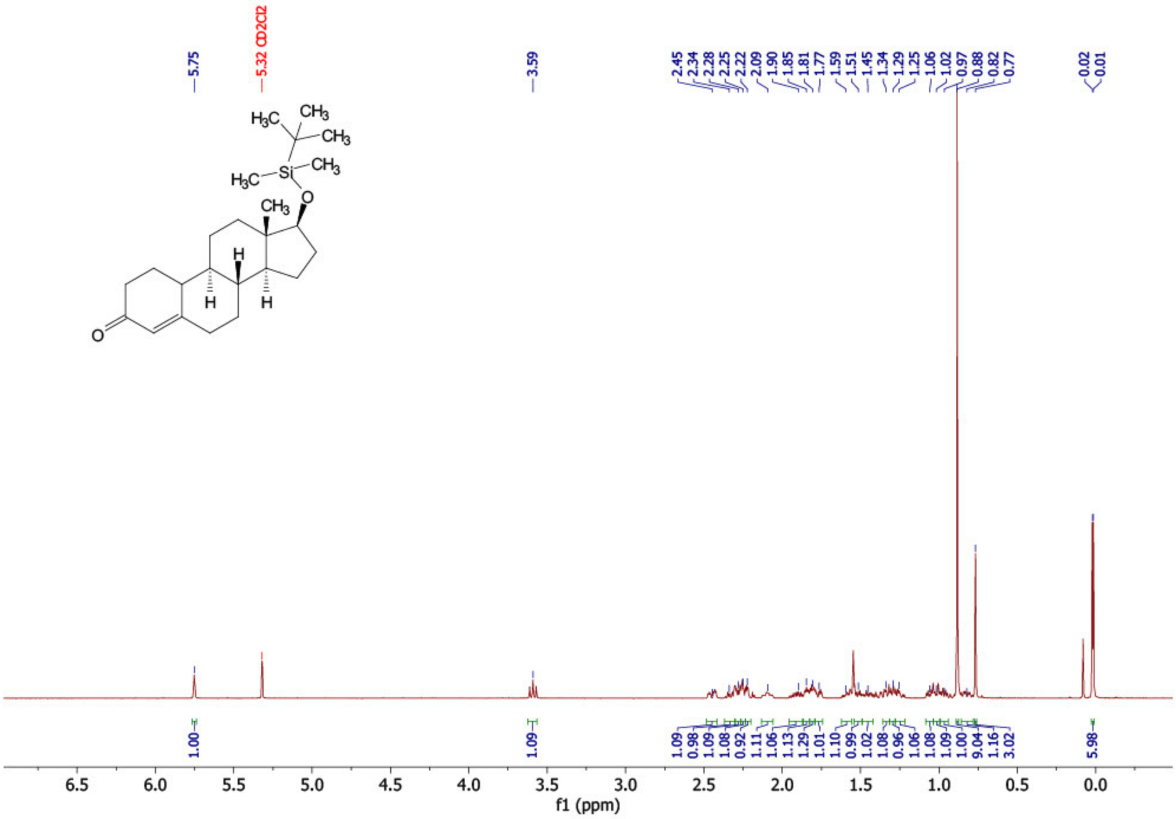

5.75
5.32 CD2Cl2
3.59
2.45
2.34
2.28
2.25
2.22
2.09
1.90
1.85
1.81
1.77
1.59
1.51
1.45
1.34
1.29
1.25
1.06
1.02
0.97
0.88
0.82
0.77
0.02
0.01
1.00
1.09
1.09
0.98
1.09
1.08
0.92
1.11
1.06
1.13
1.29
1.01
1.10
0.99
1.02
1.08
0.96
1.06
1.08
1.09
1.00
9.04
1.16
3.02
5.98
f1 (ppm)

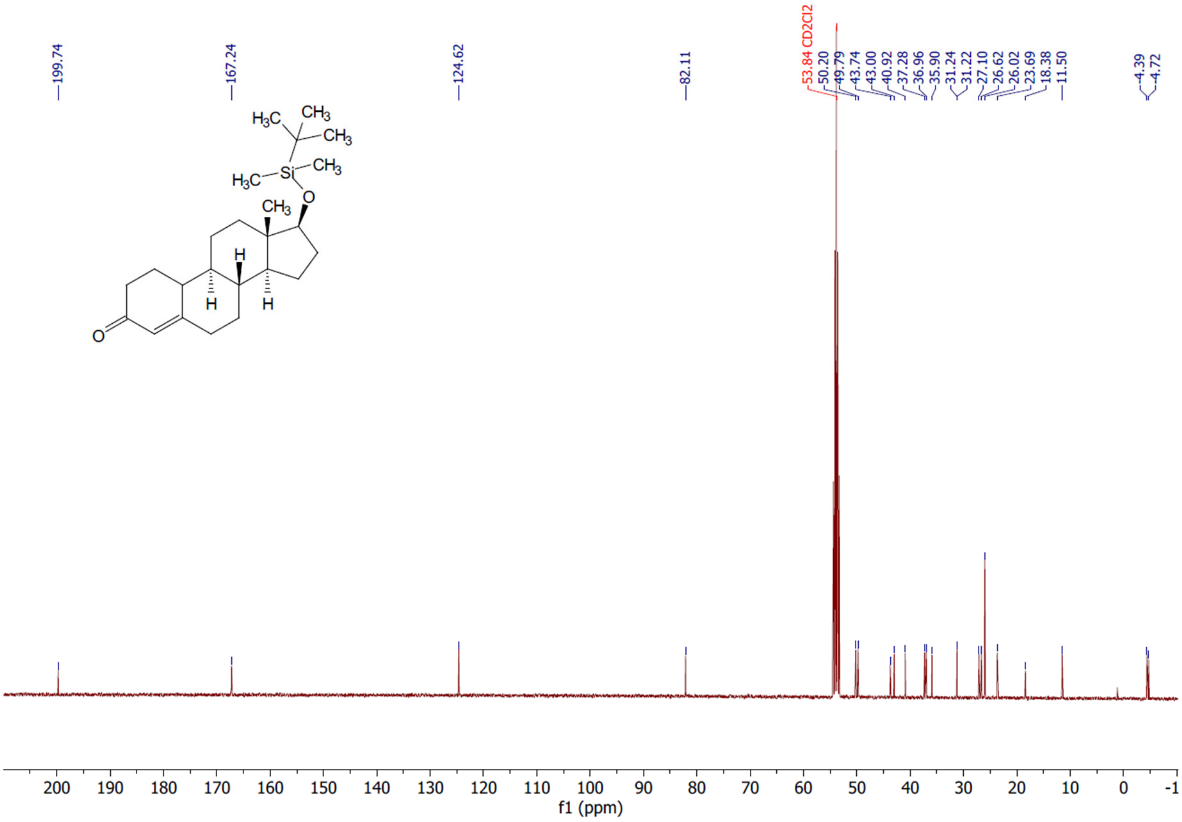

199.74
167.24
124.62
82.11
53.84 CD2Cl2
50.20
49.79
43.74
43.00
40.92
37.28
36.96
35.90
31.24
31.22
27.10
26.62
26.02
23.69
18.38
11.50
-4.39
-4.72
f1 (ppm)

Compound S2

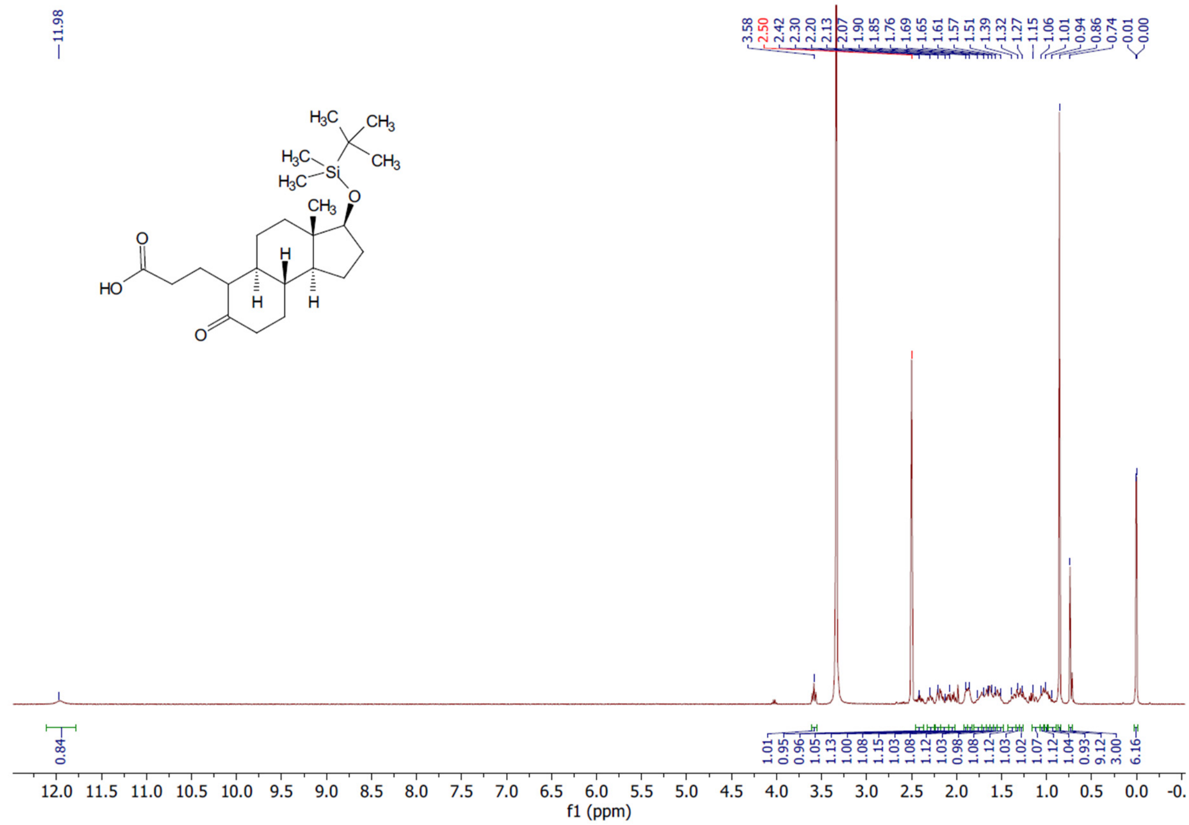

11.98
3.58
2.50
2.42
2.30
2.20
2.13
2.07
1.90
1.85
1.76
1.69
1.65
1.61
1.57
1.51
1.39
1.32
1.27
1.15
1.06
1.01
0.94
0.86
0.74
0.01
0.00
H3C
CH3
H3C
Si
CH3
H3C
O
CH3
O
H
HO
H
H
O
0.84
1.01
0.95
0.96
1.05
1.13
1.00
1.08
1.15
1.03
1.08
1.12
1.03
0.98
1.08
1.12
1.03
1.02
1.07
1.12
1.04
0.93
9.12
3.00
6.16
f1 (ppm)

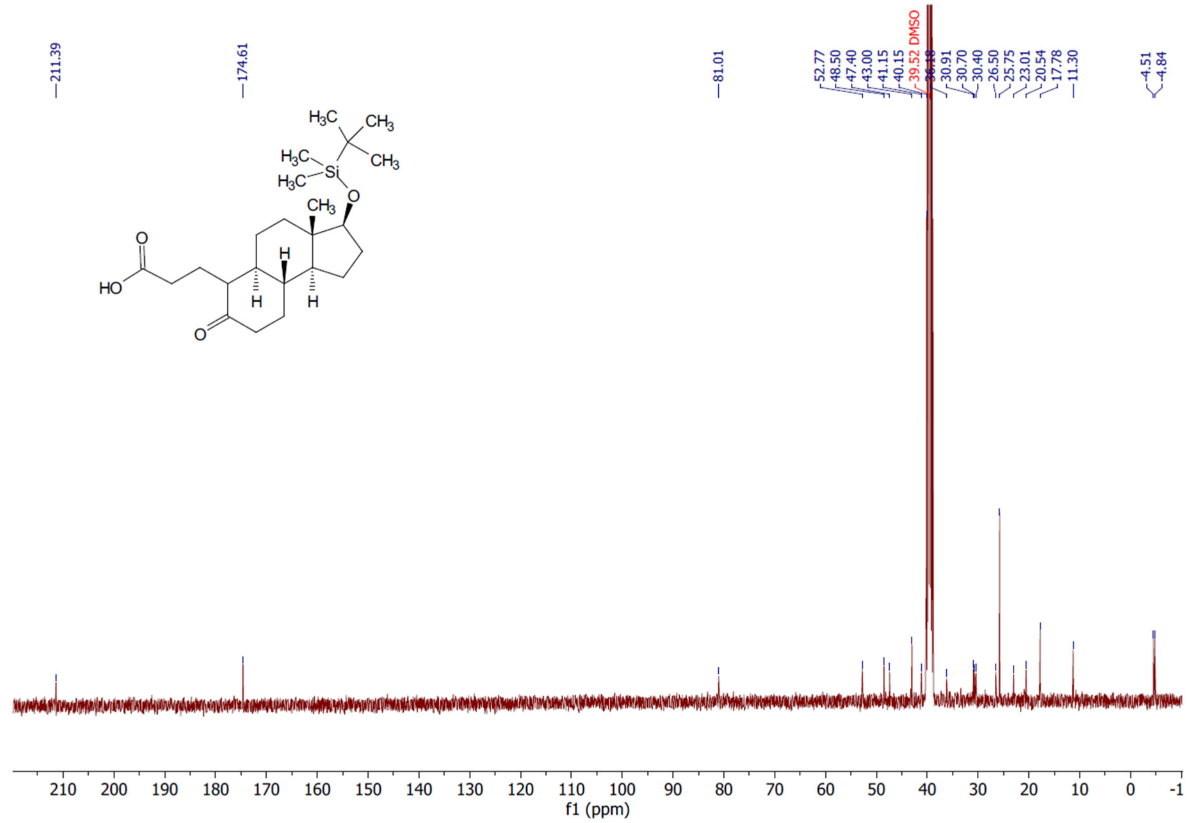

211.39
174.61
81.01
52.77
48.50
47.40
43.00
41.15
40.15
39.52 DMSO
36.18
30.91
30.70
30.40
26.50
25.75
23.01
20.54
17.78
11.30
-4.51
-4.84
H3C
CH3
H3C
Si
CH3
H3C
O
CH3
O
H
HO
H
H
O
f1 (ppm)

Compound S3a

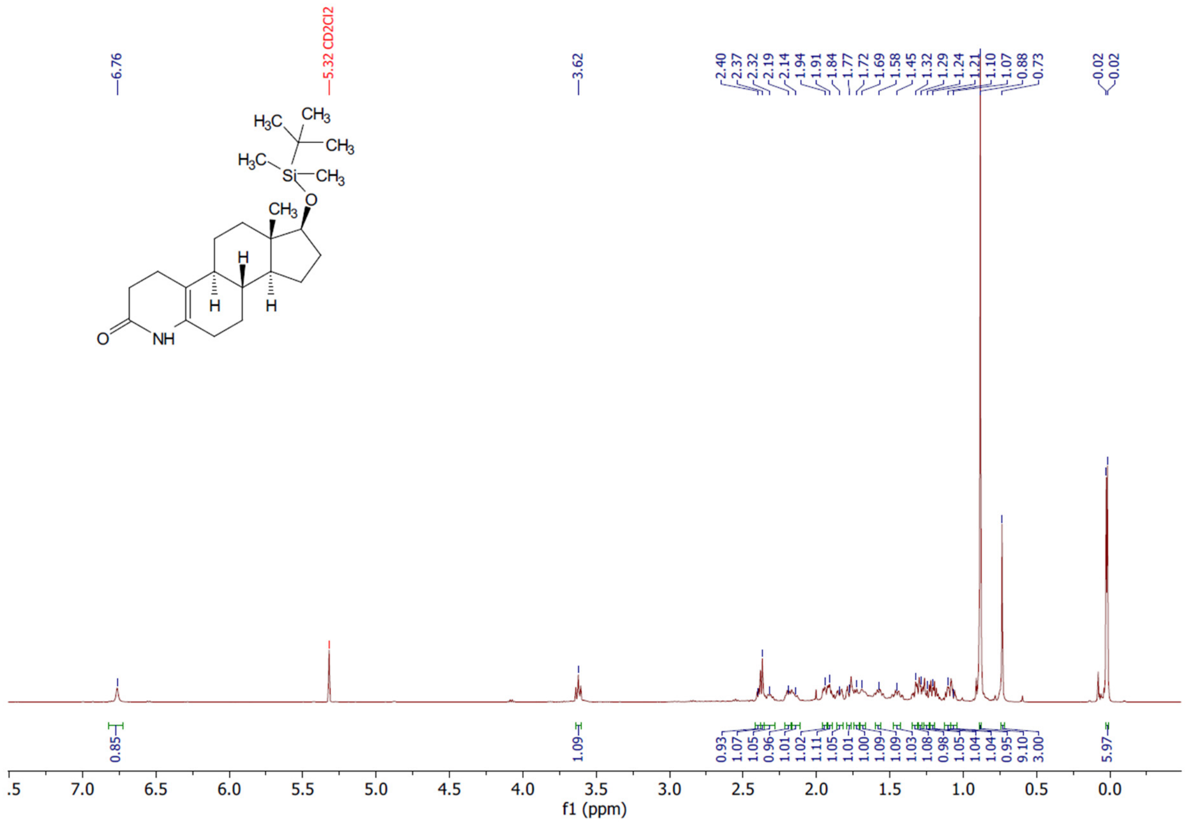

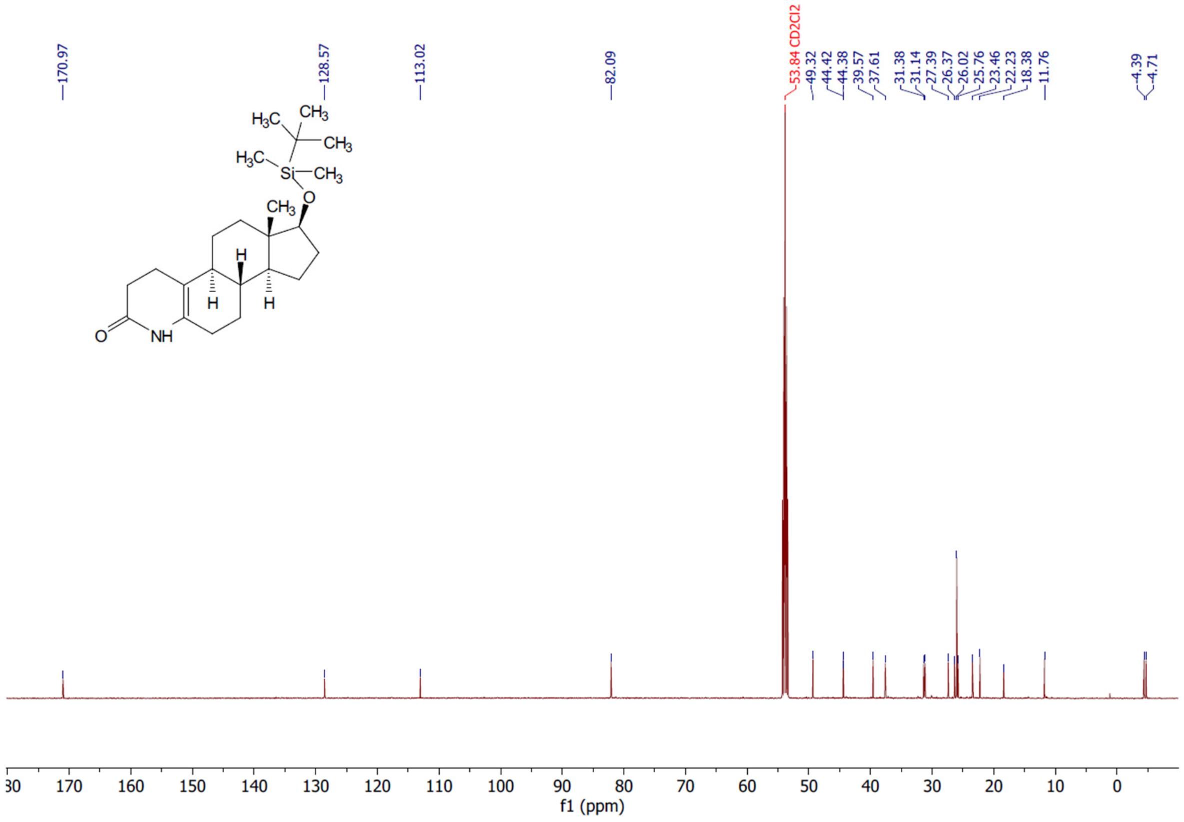

Compound S3b

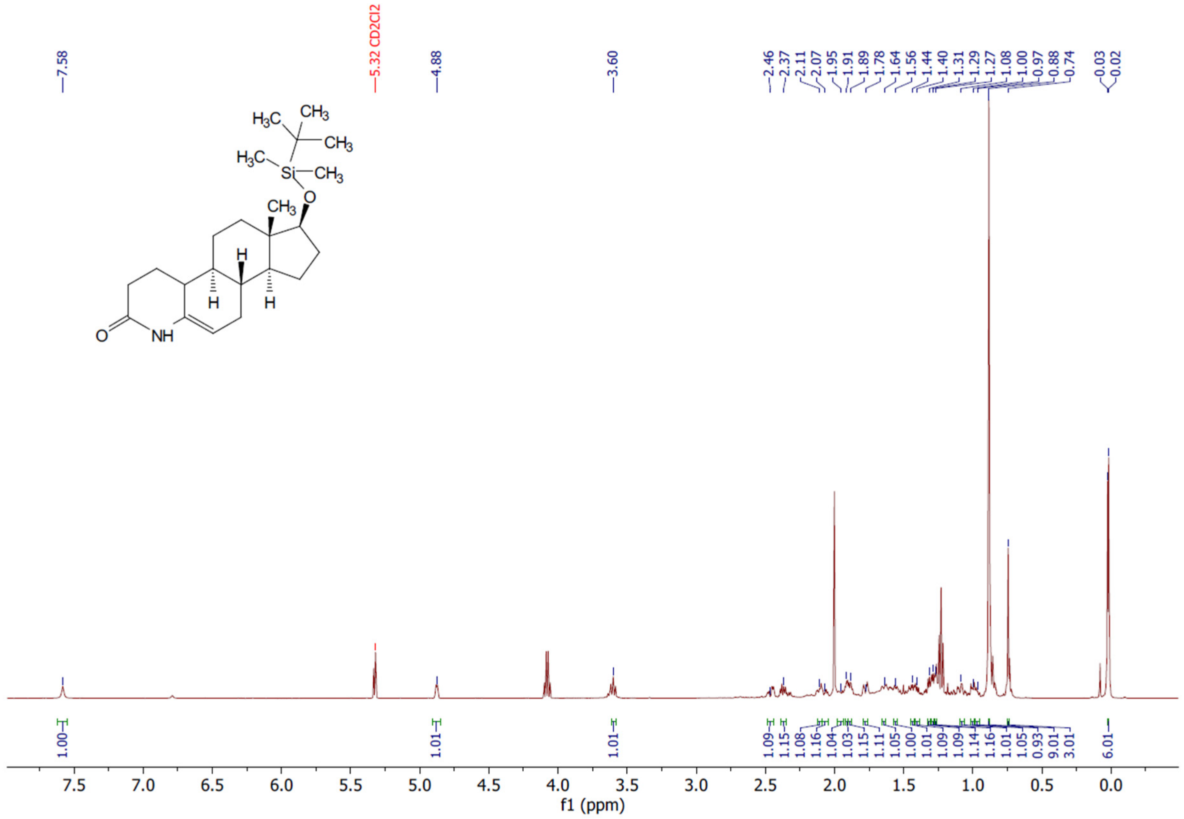

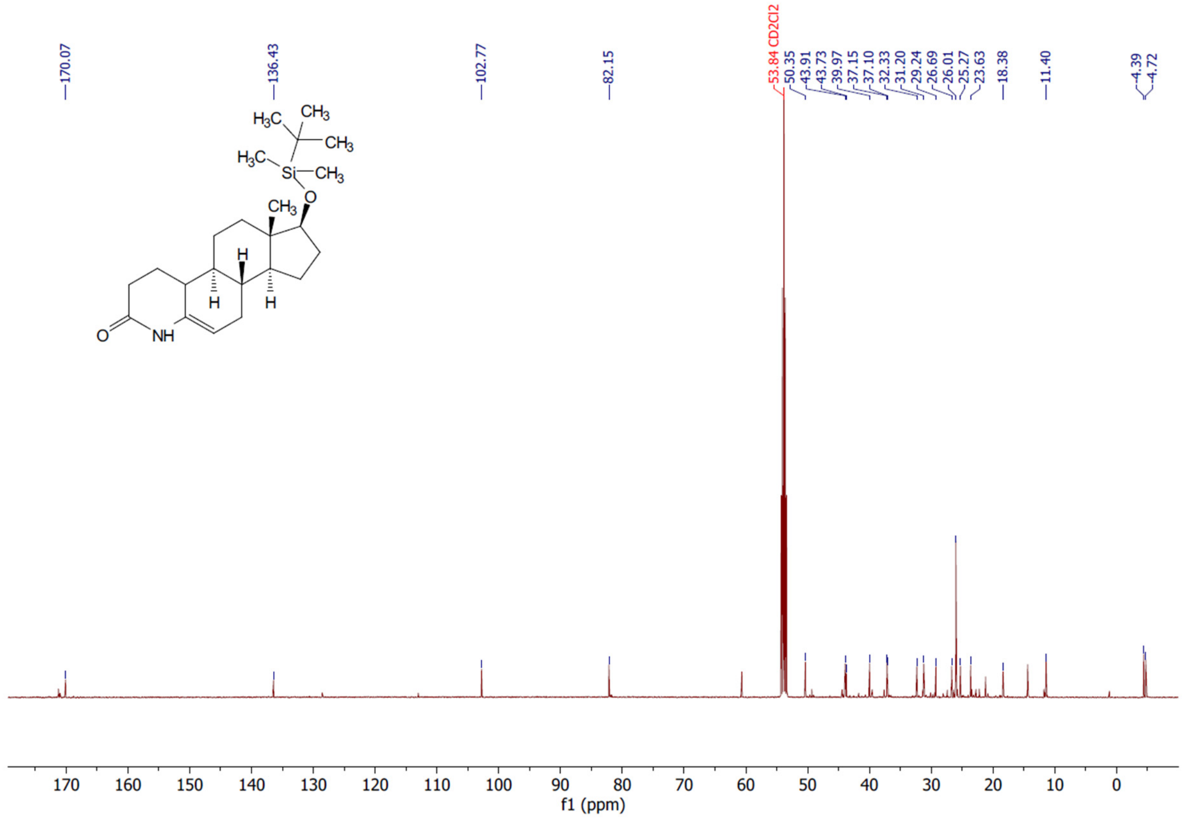

Supplement: Supplementary file 1 [file molecules-28-07428-s001.zip › molecules-2689966-supplementary.pdf]
